# A digitally controlled silicon quantum processing unit

**DOI:** 10.1038/s41586-026-10754-7

**Published:** 2026-07-29

**Authors:** Members of the HRL Quantum Team and Collaborators, Michael Abraham, Edwin Acuna, Tower S. Adams, Moonmoon Akmal, Matthew R. Alfaro, I. Alvarado, Jacob Amontree, Carter Andrews, Reed W. Andrews, Michael Antcliffe, André R. Asencio, Ryan M. Avila Batres, Cynthia D. Baringer, David W. Barnes, Katherine M. Beech, Russell G. Blakey, Zachery T. Bloom, Aaron J. Bluestone, Jacob Z. Blumoff, Matthew G. Borselli, Koel A. Bose, Brydon Boyd, Jacob T. Boyer, Teresa L. Brecht, Christopher C. Brough, Rex A. Brown, Steven L. Brown, Tyler A. Cain, John B. Carpenter, Stephen Carr, Faustin W. Carter, Mitchell Casanova, Jacob L. Chambers, Matthew D. Chambers, Khamsorn L. Chanthavong, James M. Chappell, Rhian Chavez, Kevin C. Chen, Peter S. Chen, Maxwell D. Choi, Krishna Choudhary, Matthew N. H. Chow, Justin E. Christensen, Aaron M. Chronister, Andrew M. Clapper, Abigail A. Coker, Michael D. Cornelius, Albert E. Cosand, Ian T. Counts, Edward T. Croke, Gregory M. Crosswhite, Adam Dally, Erik S. Daniel, Tuan A. Dao, Dominic Daprano, Tiffany Davis, Neha Deshpande, Rachel S. Dey, D. Scott Diamond, Claire E. Dickerson, J. P. Dodson, James B. Dragan, Marc Dvorak, Lisa F. Edge, Charles R. Elliott, Kenneth R. Elliott, Kevin Eng, Jacob Fast, Colin P. Feeney, David J. Fialkow, Dylan H. Finestone, Micha N. Fireman, Bryan H. Fong, Trevor M. Fowler, Sean Frazier, Kiera L. Fuller, Christina A. C. Garcia, Kacy L. Garstka, Kara C. Garvey, Zachary A. Geiger, Galen R. Gledhill, Caleigh M. Goodwin-Schoen, Joseph L. Goralka, Bradley W. Greene, Hrayr K. Gurgenian, Sieu D. Ha, Wonill Ha, Nathanial R. Hapeman, Brooke M. Hardesty, Jim W. Harrington, Patrick M. Harrington, Thomas R. B. Harris, Ben M. Harrison, Anthony T. Hatke, Robert R. Hayes, Kevin He, Raul Hernandez Garcia, Ryan M. Hickey, Jocelyn Hicks-Garner, Alex Hirman, Donald A. Hitko, David Ho, Holland Y. Ho, Vinh S. Ho, nathan holman, Adam Holmes, Nerys Huffman, Daniel R. Hulbert, Eric B. Isaacs, Clayton A. C. Jackson, Logan Jaeger, Ian Jenkins, Cameron Jennings, Paul C. Jerger, B. Johnson, Aaron M. Jones, Michael P. Jura, Adour V. Kabakian, Raj M. Katti, Tyler Keating, Joseph Kerckhoff, Joseph D. Kern, Isaac Khalaf, Aditya Kher, Jake J. Kim, Erich W. Kinder, Andrey A. Kiselev, William F. Koehl, Patrick W. Krantz, Thaddeus D. Ladd, Pierce G. Laing, Sanaaya Lakdawala, Nathan J. Lang, Robert Lanza, Elias Lawson-Fox, Dustin Le, Kangmu Lee, Nathan R. A. Lee, Jaime Lerma, Mark P. Levendorf, Alwina R. Liu, Henry Lizarraga, Aurelio Lopez, Hoa C. Ly, Torrey T. Lyons, Theodore K. Macioce, Matthew M. Mackey, John K. Maeda, Ryan M. Martin, Daniel S. Matic, Justine W. Matten, Gavin C. Mazur, Max S. McCready, Olivia Means, Kevin E. Millner, Ivan Milosavljevic, Matthew Morris, Susan L. Morton, Samuel Mumford, Bryce D. Murley, Robert G. Nagele, Taro A. Naoi, Cameron R. Nelson, Georgia A. Newman, David B. Nguyen, Tina Niknejad, Rebecca N. Nishide, Liam C. O’Brien, Colin B. E. O’Keefe, Riley P. O’Neil, Andrew E. Oriani, Anthony F. Ortiz, John J. Ottusch, Andrew Pan, Pamela R. Patterson, Uttam Paudel, Julius C. Perez, Christi A. Peterson, Vu T. Phan, Nickolas H. Pilgram, Clifford E. Plesha, Winston Pouse, Eric M. Prophet, Daniel R. Queen, Nicholas Quirk, Kate Raach, Matthew T. Rakher, Matthew D. Reed, Brandon D. Reynolds, Luke D. Robertson, Zechariah Rogers, Yakov Royter, Matthew J. Ruiz, Golam Sabbir, Roshan Sajjad, Christopher D. Sanborn, Rachel H. Sarmiento, Christian J. Schnaible, Cole Scott, Nicholas M. Sebastiani, Eric M. Segall, Alen Senanian, Adalberto Sicairos, Shariq Siddiqui, Kartik Singh, Aaron Smith, Daniel E. Smith, Robert S. Smith, Sarah F. Sontag, Emilio A. Sovero, Kevin C. Staley, Andrea Su, June Suh, Bo Sun, Danny Sun, Christopher M. Swank, Noah Swimmer, Mariano J. Taboada, Bryan J. Thomas, Yessica Torres, Jeremy W. Touve, Alan Tran, Ivan Tran, Chantang Tsen, Skylar Turner, Miguel Valencia, Irma Valles, James R. van Meter, Nicholas D. VanRensselaer, Franklin Vartanian, Daniel Volya, Zachary J. Vrba, Phuong Hong Vu, Annette L. Wagner, John Wallner, Michael P. Walsh, Shuoqin Wang, Tong Wang, Daniel R. Ward, Aaron J. Weinstein, Terry B. Welch, Thomas V. Westrick, Evan T. White, Randall M. White, Samuel J. Whiteley, Gananath Wijeratne, Parker Williams, Jack T. Wilson, Courtney P. Wilt, Deborah E. Winklea, Onnik Yaglioglu, Daniel Yap, Clifford S. YoungSciortino, Daniel Zehnder, Andrew Ziegler

**Affiliations:** 1 HRL Laboratories, LLC, Malibu, CA USA; 2 The Boeing Company, El Segundo, CA USA

**Keywords:** Quantum information, Electronic and spintronic devices, Qubits

## Abstract

Commercially relevant quantum computers will require large numbers of high-performing qubits that can be manufactured, integrated and controlled at scale. Silicon exchange-only qubits^1–10^ are a strong candidate modality owing to their control-signal simplicity and compatibility with advanced semiconductor manufacturing^11–13^, but questions remain around the achievability of sufficiently low noise and a scalable control and wiring solution^13–19^. Here we introduce a quantum processing unit composed of a custom-designed cryogenic complementary metal–oxide–semiconductor (CMOS) controller, a high-density superconducting ribbon cable and a low-noise exchange-only qubit device. The quantum chip features a 3-rail array of 54 exchange-coupled quantum dots, configurable to host up to 18 exchange-only qubits. We integrate and use these components to demonstrate qubit performance for both single-qubit and entangling operations that advances the exchange-only state of the art^7,8,10^ by an order of magnitude. We further validate this system by implementing a distance-5 repetition code^20^ and a distance-2 quantum error-detecting code^21–26^ and then make detailed comparisons with simulations. Our work facilitates the development of future utility-scale quantum computers with manageable operational and capital requirements.

## Abstract (web summary)

A silicon quantum processing unit executes high-fidelity multiqubit circuits, with all time-varying control signals generated by a digitally programmed cryogenic complementary metal–oxide–semiconductor controller and delivered to the low-noise, exchange-only qubit device through a high-density superconducting ribbon cable.

## Main

From transistors to integrated circuits to silicon (Si) CMOS, the dominant computing technologies of the modern era have been determined by advantage in cost-effective manufacturability. Building a commercially viable quantum computer will demand the same—efficient fabrication not just of the qubit chip but also of the systems responsible for control-signal generation and delivery. The rapid advancement of the scale and fidelity of Si spin qubits^10,12,26–31^, driven in part by their compatibility with advanced semiconductor processes, has outpaced that of other system components^13–19^. In resolving this disconnect, a system architect must choose between placing control-signal hardware at room temperature (burdened by the complexity of routing numerous high-fidelity analogue links)^10–12,26,27,30,31^, co-locating it with the millikelvin qubits (with tyrannical cooling requirements)^18,32^ and using an intermediate temperature^13–17,33,34^. This third option enjoys greater Carnot efficiency and avoids the room temperature ‘wiring bottleneck’^33^, but must transmit numerous signals to millikelvin qubits while maintaining thermal isolation.

**Fig. 1:**
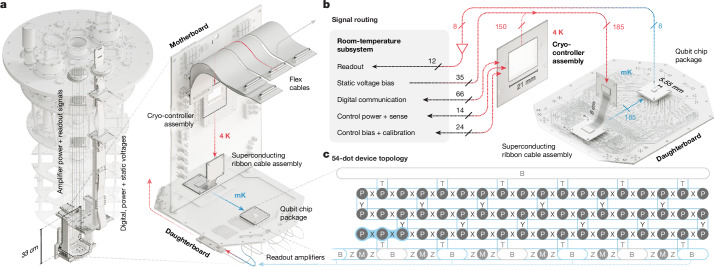
An integrated quantum processor. **a**, The QPU is a tightly integrated system consisting of a controller motherboard at 4 K, a qubit daughterboard at millikelvin and a superconducting ribbon cable bridging the two (Extended Data Fig. 3). It is installed at the bottom of a Bluefors XLD1000 dilution refrigerator and connected to room temperature with flex cables and coaxial lines (see ‘Flex cable’ in Supplementary Information). Custom-designed transimpedance amplifiers (see ‘4 K transimpedance amplifier’ in Supplementary Information) are thermalized to 4 K but are located below the daughterboard to minimize capacitance. **b**, The room-temperature subsystem provides digital signalling and static current and voltage biases. Qubit readout signals are also digitized at room temperature, although we anticipate that future systems will incorporate that function inside the cryostat^19^. The ribbon cable transmits all 150 time-varying qubit control signals and 35 static device biases from 4 K to millikelvin (mK) with minimal thermal load. **c**, Topology schematic of the quantum chip. The 54 dots are arrayed in 3 sparsely coupled rails and support up to 18 encoded qubits arrayed in a 3 × 6 square lattice with nearest-neighbour coupling. We flexibly partition connected trios of dots into qubits, motivated by yield, performance or desired connectivity^8^. ‘P’ gates control dot occupancy and adjacent ‘X’ and ‘Y’ gates control the tunnel coupling between dots. Dot loading, measurement and qubit initialization utilize reservoirs accumulated by 8 ‘B’ gates coupled to the array by 12 ‘T’ gates. ‘M’ and ‘Z’ gates form 6 dot charge sensors used for device tune-up and qubit-state readout^46^. Various field gates deplete charge under inactive regions. The biases applied to these electrodes are determined with a combination of manual and automated methods (see ‘Tune-up and calibration’ in Supplementary Information). Credit: **a**(left), Bluefors.

**Extended Data Fig. 1:**
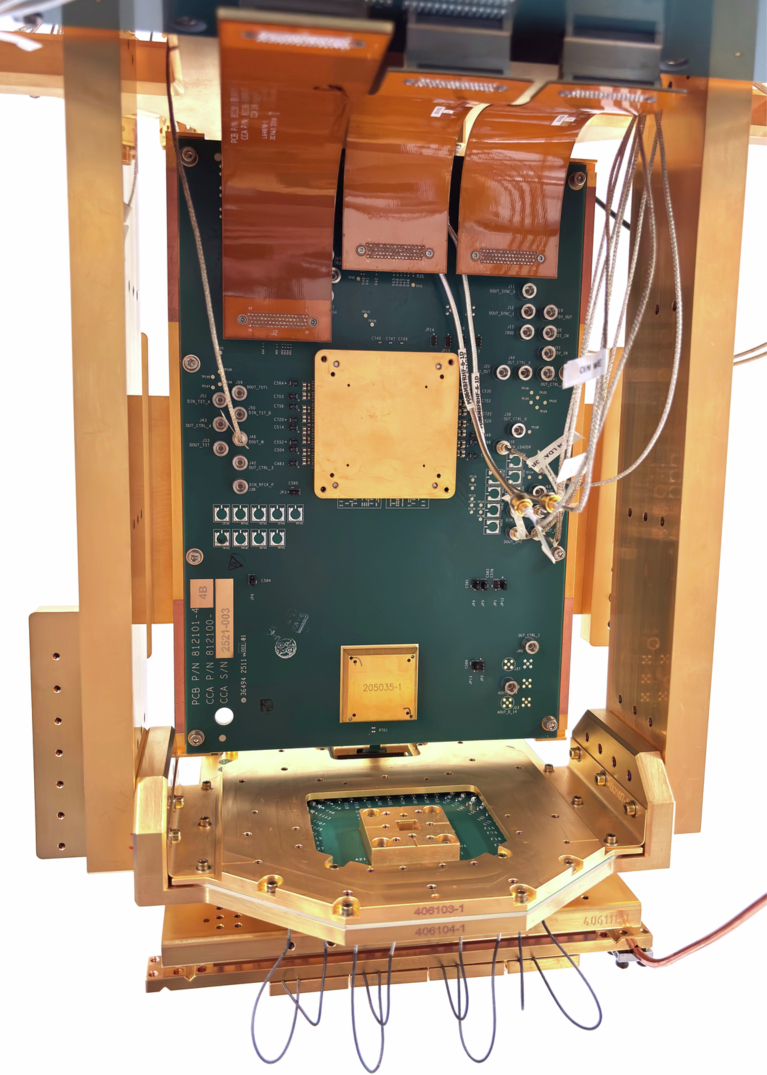
Integrated QPU. The motherboard (top) houses the cryo-controller and the daughterboard (bottom) hosts the qubit chip. A light-tight metal box encloses the daughterboard to shield the qubit chip from ambient radiation. The two are bridged by a superconducting ribbon cable (largely obscured here by the motherboard; the ribbon connects to the side opposite the camera’s perspective – see Extended Data Fig. 3). TIA amplifiers are located beneath the daughterboard to minimize capacitance but thermalized to 4 K.

Our system follows the third option, using a fully custom low-power CMOS chip operating at 4 K to generate all time-varying qubit control signals. We resolve the thermal isolation problem by routing those signals through a high-density, low-thermal-conductance superconducting ribbon cable to the quantum chip at millikelvin (Fig. 1a). As integrated, this ‘quantum processing unit’ (QPU; photograph in Extended Data Fig. 1) has modest requirements for its connections to room temperature: only digital communication, qubit readout and static biases (Fig. 1b). The superconducting interconnect maintains excellent signal integrity and bandwidth while also serving as a thermal standoff, preventing the 4-K controller from unduly heating the millikelvin stage. Exchange-only qubits also pair well with power-constrained CMOS signal generation as their required control waveforms (shape-insensitive baseband voltage pulses) are similar to standard digital signals. The qubit chip, ribbon and controller are each fabricated with semiconductor wafer processes, a strong indicator of manufacturability. The QPU is designed for the function that will most occupy any utility-scale quantum computer: error correction. Below, we verify this capability with repeated syndrome measurements in [3, 1, 3] and [5, 1, 5] repetition codes as well as the [[4, 2, 2]] quantum error-detecting code.

## Quantum chip

**Extended Data Fig. 2:**
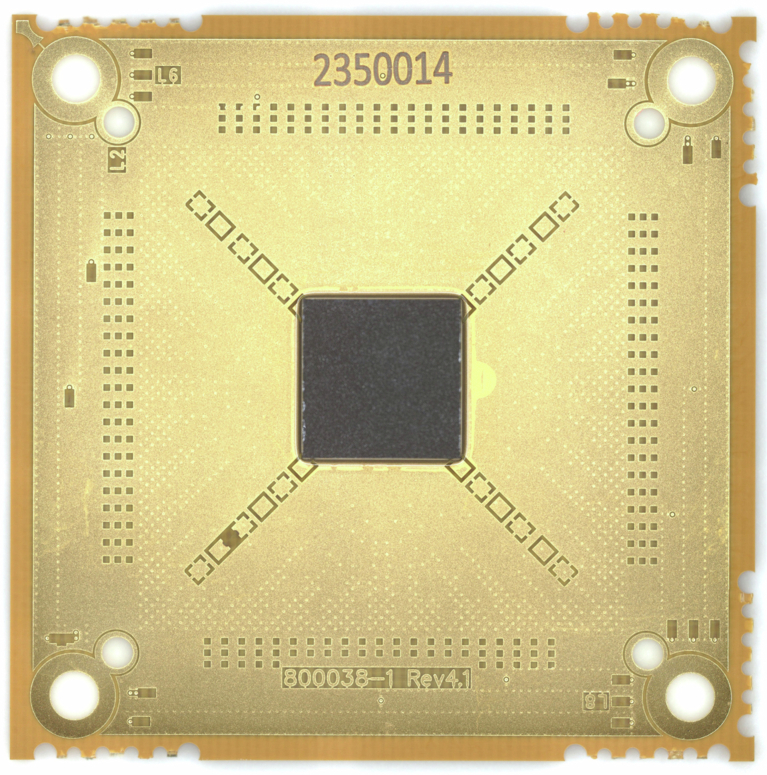
Photograph of a packaged qubit chip. The Si qubit chip die (black square) is bump bonded to a fine-pitch laminate LGA package (gold).

Our qubit devices each feature 54 low-noise exchange-coupled quantum dots distributed over three rails (Fig. 1c). Qubits are encoded in the joint spin state of three individual electrons in three dots (Methods). Electrons are vertically confined in a Si/SiGe quantum well, which is engineered to increase valley splitting. Si and germanium (Ge) in the heterostructure are isotopically enhanced to reduce magnetic noise. Lateral confinement is achieved using electrostatic gates patterned on top of the heterostructure in a proprietary 200-mm wafer foundry process. The intrinsic charge noise of these devices is more than an order of magnitude lower than our previous single-layer etch-defined gate electrode (SLEDGE) technology^6–8^. Additional device details can be found in the Methods. Following fabrication, wafers are probed to select high-yielding die then diced and bump bonded to a fine-pitch laminate land grid array (LGA) package (Extended Data Fig. 2). Devices are installed on a daughterboard thermalized to the mixing chamber of a dilution refrigerator. When fully integrated, we observe an average electron temperature of 150 mK (see ‘Environmental control’ in Supplementary Information).

## Superconducting interconnect

**Extended Data Fig. 4:**
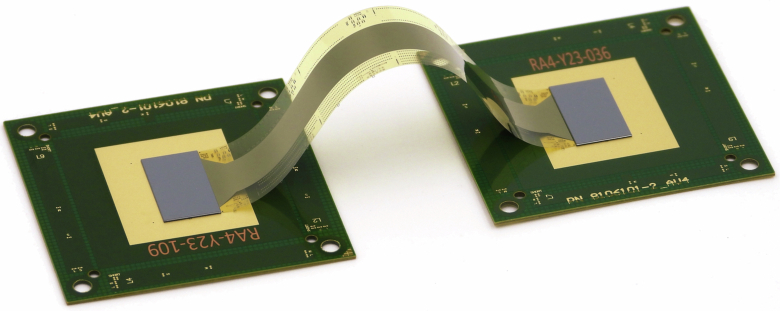
Photograph of ribbon cable assembly. The ribbon cable is indium-bump bonded to a 10-layer laminate board on each end that interfaces with printed circuit boards using a land-grid spring-probe array.

A high-density superconducting ribbon cable routes signals from the controller to the qubit chip. The cable is fabricated from niobium (Nb) on polyimide^35,36^ using standard semiconductor wafer processes. Although 296 coaxial signal lines are arranged in a single layer approximately 1 cm wide, crosstalk within the cable is under −80 dB up to 10 GHz. The ribbon maintains an effective thermal standoff owing to its use of superconducting metal and small feature sizes, conducting less than 10 μW from the 4-K stage to the mixing chamber. Laminate adapters at both ends allow the cable to be reversibly attached to both the daughterboard and the motherboard (Extended Data Fig. 4). The round-trip electrical length from controller to qubit is roughly 3 ns.

## Cryogenic CMOS controller

**Fig. 2:**
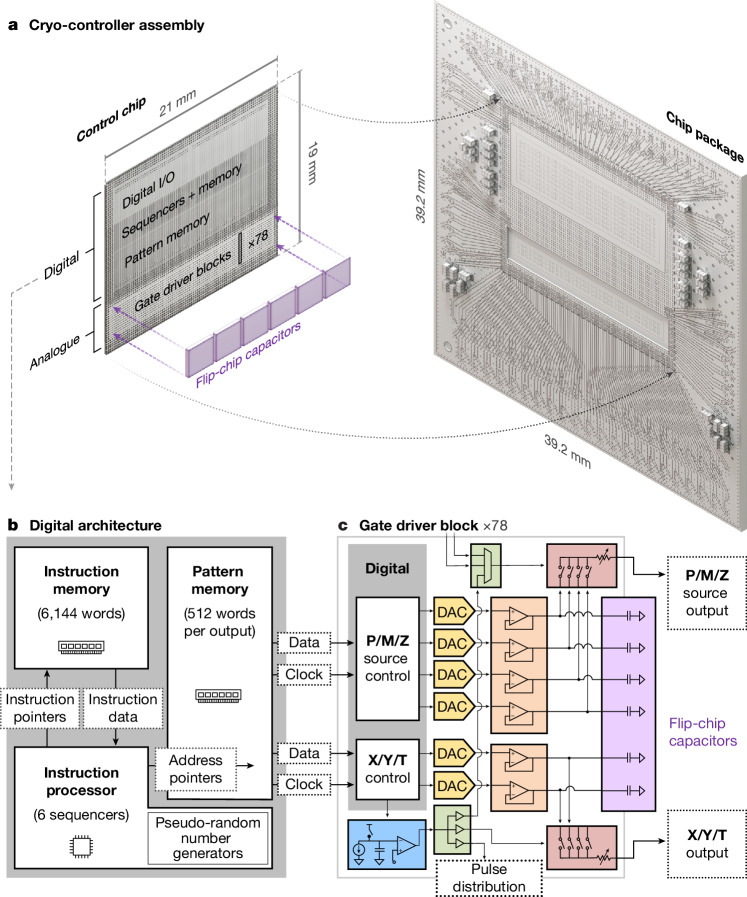
The cryogenic CMOS controller. **a**, The controller is a multi-component assembly: custom high-density capacitor arrays are bump bonded to the CMOS die, which is then integrated into a fine-pitch laminate LGA package (Extended Data Fig. 5). The CMOS die comprises roughly 70 million transistors and has 4 distinct functional areas. **b**, The digital architecture is responsible for interpreting instructions and supplying every analogue block with a pattern memory pointer every clock cycle. **c**, The gate driver layer is composed of an array of 78 nearly identical analogue blocks (one pictured) collectively comprising 156 output channels, 366 DACs and 78 pulse generators (see ‘Cryo-CMOS qubit controller’ in Supplementary Information). The DACs (yellow, 0 to 1 V, root-mean-square step size <10 μV) are buffered by amplifiers (orange) with output capacitance provided by the capacitor arrays (purple). Each exchange gate has a uniquely associated pulse generator (blue) that emits timing signals (typical duration 400 ps to 6 ns, with approximately picosecond resolution). To actuate symmetric exchange operation^47^, these signals are distributed (green) to switch drivers (red) for the X gate and its neighbouring P gates, generating synchronized voltage waveforms (the 10%-to-90% rise time *t*_10−90%_ ≈ 150 ps). Each gate driver block has a local digital module to store configurable parameters such as output impedance and filtering.

The cryo-controller (Fig. 2a) is a mixed-signal system-on-chip fabricated in a commercial 130-nm radio-frequency (RF) CMOS process. Circuit designs are optimized at the transistor level for performance at 4 K using a combination of design practices and circuit techniques specifically developed for cryogenic operation. The controller has three primary functions: device tune-up, qubit-state preparation and measurement (SPAM) and qubit-state manipulation. The first two functions are achieved by dynamically changing the digital-to-analogue converter (DAC) output voltages and the third with fast, time-modulated, gate-synchronized switching between DAC voltages that are held constant.

The controller’s digital architecture (Fig. 2b) features an autonomous, multi-sequencer engine that operates at up to 250 MHz and uses a custom instruction set architecture for qubit control. On-chip pseudo-random number generators enable memory-efficient characterization of qubit performance. The controller consumes ≤3.5 W in typical operation (see ‘Power consumption’ in Supplementary Information). Pattern memory associated with each output stores parameters for voltages and pulse widths. User programs are converted into machine code using a co-designed compiler and stored in a shared pool of 6,144 words of instruction memory. Every clock cycle, each of the sequencers interprets an instruction and updates a pattern memory pointer specifying analogue behaviour to user-assigned output blocks (Fig. 2c). Once initialized, the program runs without further communication with room-temperature electronics. The digital architecture uses a 52-line parallel bus and a serial peripheral interface for configuration, diagnostics and memory readback.

## Qubit performance

**Fig. 3:**
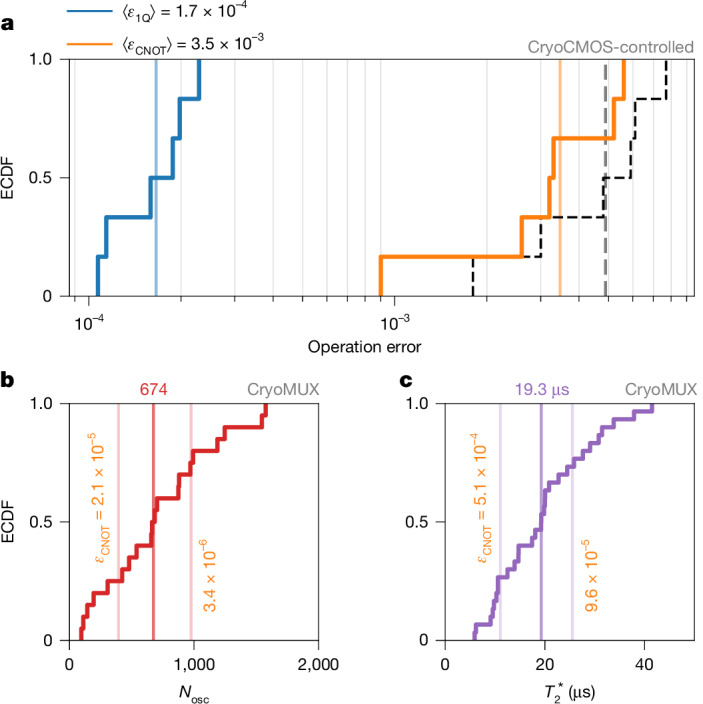
Qubit performance. **a**, Empirical cumulative distribution functions (ECDF) for gate error of cryo-controlled exchange-only qubits. Mean errors of 1.7 × 10^−4^ for single-qubit gates (*ε*_1Q_, blue) and 3.5 × 10^−3^ for CNOT (*ε*_CNOT_, orange) are indicated by vertical bars. Single-qubit gates, each composed of four exchange pulses, are characterized with blind randomized benchmarking (BRB)^5^. CNOT sequences^2,3,7^ are composed of 37 pulses in 45 time steps; some time steps intentionally omit pulses for purposes of optimizing magnetic decoupling or inter-symbol interference (see ‘Gate error predictions for ISI and crosstalk’ in Supplementary Information). The pulse cadence is varied between 12 ns and 28 ns, depending on the operation and qubit. The lowest reproducible CNOT error was 9 × 10^−4^. Sequence performance is characterized with an interleaved BRB variant (see ‘Blind randomized benchmarking (BRB)’ in Supplementary Information). The dashed line is the fidelity of CNOT gates with simultaneous dynamical decoupling applied to all other qubits; this degradation is consistent with their slightly slower pulse cadence. **b**, Representative charge noise data acquired using a cryoMUX system from a single device similar to that used in **a**. Vertical lines indicate the median (674) and quartile (394 and 977) number of exchange oscillations before 1/*e* amplitude decay, *N*_osc_, in an experiment sweeping evolution duration at constant exchange energy^47^. Orange vertical text along the quartiles represents the simulated error contribution to CNOT error due to those values of charge noise. **c**, Magnetic noise data taken from unique dot pairings on the same device in the same cryoMUX system. Vertical lines indicate median (19.3 μs) and quartile (11.1 μs and 25.6 μs) decay envelope of a singlet decay experiment, ${T}_{2}^{* }$ (ref. ^48^). Simulated contributions to CNOT error are again annotated at quartile values.

Verifying gate performance is a principal consideration as it strongly influences the overhead required for quantum error correction (QEC). As shown in Fig. 3a, the QPU used for the [[4, 2, 2]] experiment exhibits single-qubit and CNOT performance metrics that improve on the previous state of the art by an order of magnitude^7,8,10^. For budgeting purposes, we characterize noise intrinsic to the qubit chips using a purpose-designed ‘cryoMUX’ system. Those systems utilize a custom cryogenic demultiplexer (MUX), similar in functionality but different in operation to those in refs. ^37–42^, that is paired with room-temperature electronics and capable of controlling a configurable subset of nine dots. In Fig. 3b,c, we show charge and magnetic noise data from a qubit chip of the same type as those used for the multiqubit experiments. The metric *N*_osc_ characterizes error due to intrinsic charge noise for spins undergoing exchange, and the metric ${T}_{2}^{* }$ characterizes error due to magnetic noise for idling spins. The intrinsic charge noise reflected by these *N*_osc_ measurements shows improvement on the previous state of the art by a factor of ten^8,10^. Errors resulting from the mean values of *N*_osc_ and ${T}_{2}^{* }$ would collectively contribute only 0.02% to absolute CNOT error. Instead, modelling and additional system characterization indicate that ‘extrinsic’ terms such as static magnetic-field gradients and contextual pulse miscalibration dominate observed error.

## Repetition codes

**Fig. 4:**
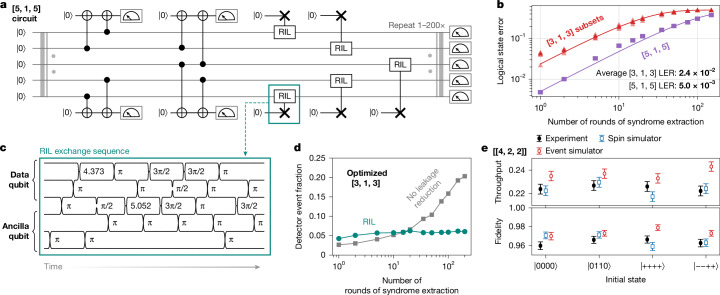
Multiqubit validation results. **a**, Simplified circuit for the distance-5 repetition code. Classical information is encoded in 5 data qubits that are repeatedly interrogated for bit-flips using 2 ancilla qubits. Each syndrome extraction round is broken into 2 waves and ancillae are re-used. A single instance of the 200-round experiment consists of 1,805 initializations, 805 measurements and 335,422 exchange pulses. **b**, We sweep the number of syndrome extraction rounds per shot in the [5, 1, 5] sequence quasi-logarithmically from 1 to 200 and repeat each 50,000 times. The syndrome data, interpreted with a naive parity check decoder, indicate an LER of 5.0 × 10^−3^ for the distance-5 code (purple) and 2.4 × 10^−2^ for the average (dark red triangles) of multiple distinct distance-3 subsets (light red triangles), yielding a distance improvement factor *Λ*_5/3_ = 4.7. **c**, The LRUs used here are composed of an ancilla initialization and a reset-if-leaked (RIL) control sequence^4^ consisting of 24 or 21 exchange pulses in 30 time steps (24-pulse variant depicted here). Each angle in the figure represents a partial swap between neighbouring spins that is actuated by corresponding controller-generated voltage pulses. **d**, The detector event fraction of a fidelity-optimized [3,1,3] experiment with (green) and without (grey) LRUs included in each syndrome extraction wave shows the effectiveness of LRUs at preventing the build-up of leakage. The LER of this instantiation of the distance-3 code was 3.2 × 10^−3^ when LRUs were included, and is lower than that extracted from the [5, 1, 5] experiment as the smaller qubit count enabled additional performance optimization. **e**, [[4, 2, 2]]-code logical fidelity (defined as the projection of the post-selected state onto the expected state) and throughput after 3 rounds of syndrome extraction are comparable for all 4 input states. Spin- and event-level simulations show relatively good model–hardware agreement (see ‘Multiqubit experiments’ in Supplementary Information). Error bars indicate $\sigma =\sqrt{p(1-p)/N}$ for *N* = 10,000 shots.

High-fidelity one- and two-qubit operations are necessary but not sufficient for effective computation—we must also show that performance does not unexpectedly degrade as circuit complexity grows. We first validate the multiqubit system performance by executing ‘classical’ repetition codes. Repetition codes are of interest because they enable qubit-efficient study of low-probability correlated errors^20^, which may unacceptably degrade QEC performance. We realize a distance-5 repetition code (simplified circuit illustrated in Fig. 4a; see ‘[5,1,5] demonstration’ in Supplementary Information) using seven exchange-only qubits. Critically, this circuit applies first-order ‘NZ1’ full-permutation dynamical decoupling^9^ to all idling qubits during all SPAM, two-qubit and leakage-reduction operations. The circuit also uses parallel state preparation, measurement and exchange operations^10^. Comparing the distance-5 logical performance (logical error rate (LER) ≈ 5 × 10^−3^) to the average performance of distance-3 subsets assessed from the same underlying data, we observe a scaling factor *Λ*_5/3_ = 4.7 (Fig. 4b). Data from this experiment show as-yet unexplained fluctuations in error rate that were not observed in either the distance-3 or [[4, 2, 2]] data discussed below (see ‘Spikes in detection events’ in Supplementary Information).

The three-spin encoding of this qubit minimizes any distinction between bit- or phase-flips in repetition codes but introduces the important factor of spin-state leakage out of the computational subspace^2–5,7^. We mitigate that concern in all multiqubit circuits shown here with the addition of leakage-reduction units (LRUs) (Fig. 4c). These gadgets conditionally reset data qubits if and only if they have leaked out of the computational space. We confirm the effectiveness of this mitigation with a distance-3 repetition code experiment using a selection of qubits optimized for [3, 1, 3] performance (see ‘[3,1,3] demonstration’ in Supplementary Information) to maximize sensitivity. As expected, we observe that the inclusion of LRUs in the syndrome extraction circuit leads to a stable detector event fraction, but their omission leads to leakage accumulation and a detector event fraction that grows with round (Fig. 4d). This stability is understood to be a prerequisite for effective QEC.

We improve confidence in our error model by exhaustively comparing the [3, 1, 3] results with theoretical predictions. In that case, a qubit-level event simulator (calibrated with measured gate error rates from blind interleaved randomized benchmarking (BIRB) experiments performed in situ and assignment fidelity measurements for SPAM) agreed with the experimental LER to 15% relative inaccuracy. We found similarly good agreement using a spin-level simulator incorporating dot-level noise measurements (*N*_osc_, ${T}_{2}^{* }$, static field gradients and so on), observed assignment fidelity and an ersatz miscalibration term. The miscalibration term was chosen to match simulated and measured CNOT BIRB error and was added to reflect errors arising from deterministic imperfections in signal generation and transmission. In this simulation, that term is responsible for approximately 80% of detection events. The agreement between these two simulators and experiment is an encouraging indication that our system exhibits emergent Markovianity and is well described by a simple and scalable performance model. More discussion of that measurement, the associated simulations and error attribution can be found in ‘Simulation’ and ‘Multiqubit experiments’ in Supplementary Information.

## Quantum error detection

We perform a final system validation by implementing a quantum error-detecting code that is potentially sensitive to error types not detected by classical codes. The [[4, 2, 2]] code, previously demonstrated in trapped ion qubits^21,22^, superconducting qubits^23^ and neutral atom qubits^25^, embeds two logical qubits into four physical qubits and enables the detection of any single-qubit (weight-1) error. Similar syndrome measurements^26^ and logical operation^24^ were recently demonstrated in silicon qubits. Each round of syndrome extraction entails two weight-4 measurements (*XXXX* and *ZZZZ*), interleaved flag qubit measurements and leakage reduction.

We implement this code with six physical qubits on a system similar to, but slightly improved from, that of the [5, 1, 5] experiment (see ‘[[4, 2, 2]] demonstration’ in Supplementary Information). During syndrome extraction, we compile all single-qubit gates into composite two-qubit operations. We prepare the data qubits into each of four different initial states $| 0000\rangle ,| 0110\rangle ,| ++++\rangle$ and ∣−−++⟩ and perform three rounds of syndrome extraction before measuring their final states. The first round of syndrome measurements predictably projects these into different logical states ($| \bar{0}\bar{0}\rangle ,| \bar{1}\bar{1}\rangle ,| \bar{+}\bar{+}\rangle$ and $| \bar{\mathrm{-}}\bar{\mathrm{+}}\rangle$, respectively). When preparing in the *X* basis, we switch the ordering of two stabilizer measurements to project into a logical state more quickly.

We assess code performance by post-selecting on all error-detecting measurements: syndrome results, flag measurements and a last syndrome computed from the final data qubit measurements. This process ultimately rejects roughly 77% of the 10,000 experimental shots acquired per preparation basis. We then analyse the final measurements and assess a two-logical-qubit-state fidelity *F*_L_ after three rounds, finding *F*_L_ = 0.95 largely independent of the initial state (Fig. 4e). If we instead ignore the error-detecting measurements, we find an average *F*_L_ = 0.59, indicating the effectiveness of quantum error detection.

To examine correlations between error events, we turn to detector-error-model analysis^43–45^. As shown in ‘[[4,2,2]] DEM analysis’ in Supplementary Information, the detector-error-model analysis of the empirical data and of spin simulations show no statistically relevant unexpected events in the [[4, 2, 2]] experiment after appropriate grouping of events with the same weight. This indication of ‘well behaved’ errors is encouraging for larger-scale QEC.

## Towards commercial relevance

Our demonstration marks a milestone in the technological maturity of semiconductor spin qubits. Relative to previous reporting on these qubits^7,10^, we have shown transformative improvements in operational fidelity and power- and space-efficient cryogenic control. Conventional concerns such as charge noise and leakage out of the computational space are no longer limiting, nor do we see signs of valley splitting inhibiting performance. Rather, the quality of our demonstration is set by how well each of the system subcomponents are made to work together as a whole. Development now shifts to integration improvements such as magnetic hygiene, power delivery, internal signal integrity and device calibration, all having highly feasible engineering solutions. Our ability to predict system-level performance based only on local measures such as gate error and noise parametrics also indicates that the fundamental design decisions of the technology are sound.

We believe that a system design integrating 4-K cryogenic control, low-noise millikelvin exchange-only qubits, and superconducting interconnection provides a practical foundation for a utility-scale fault-tolerant quantum computer in a single, commercial cryostat. To get there, work still remains in maturing both the components and their integration. Necessary advances include a manufacturable interconnect and qubit back-end routing solution, controllers with lower power consumption per qubit, and improved device uniformity to reduce tune-up overhead. A system-level quantum architecture tailored to the connectivity, noise environment and other properties of exchange-only qubits must also be elaborated. Taken together, the scale and cost of a system anticipated by the prototype reported here position semiconductor spin qubits as a leading technology to realize commercially relevant quantum computing.

## Methods

### Qubit chip

SiGe heterostructures for the qubit chip were grown on 200-mm Si wafers by chemical vapour deposition (CVD). A strain-relaxed SiGe buffer layer was grown on top of a Si wafer^49^, terminating with a SiGe layer that matches the Ge composition of the SiGe barriers to the quantum well. The SiGe surface of the buffer received chemical mechanical polishing to planarize the surface before CVD growth of the quantum well and barriers. The barrier layers are Si_1−*x*_Ge_*x*_ grown using SiH_4_ and GeH_4_ with wafer-to-wafer alloy compositions ranging from *x* = 0.25 to *x* = 0.35. The SiGe heterostructure, enriched with ^28^Si and depleted of ^73^Ge, was engineered to increase valley splitting energy^50–53^.

Qubit chips were fabricated on the SiGe heterostructure wafers described above, leveraging CMOS-compatible process integration. This combines the proprietary, qubit-specific front-end-of-line integration with industry-standard back-end-of-line interconnect integration. Ohmic contacts and electrically inactive regions were defined by optical lithography and ion implantation of phosphorus and argon, respectively^6^; no mesa etch was utilized. The qubit gate design, with a minimum feature pitch of 70 nm, was patterned with electron-beam lithography. Gate contacts and routing were formed using a self-aligned dual damascene process with a total of three minimum-pitch routing levels. At each level, designs for vias and for routing were pattern-transferred into a hard mask using electron-beam lithography. Dry etching opened vias and routing trenches within a SiO_2_ interlayer dielectric. CVD tungsten (W) metallization, with a TiN liner, filled the vias and trenches, which were subsequently isolated using chemical mechanical planarization.

Next, three additional larger-pitch metal layers were formed to contact the W layers and fan out the routing lines to the pad pitch. These three layers were formed in a co-planar waveguide stack, with each layer patterned by optical lithography. Each layer was formed with a single damascene via made of W followed by a routing or ground plane layer composed of Nb using a subtractive integration. Aluminium bond pads were formed to contact the Nb routing and allow for wafer probing of the qubit devices. Following wafer probing, indium bumps were added to the aluminium pads and the wafers were diced to singulate each qubit chip for later flip-chip packaging.

### Exchange-only qubits

‘Exchange only’ ^1–10^ means that the only physical interactions used on electrons trapped in quantum dots are (1) initialization into antisymmetric spin-singlet states, that is, $|\uparrow \downarrow \rangle -|\downarrow \uparrow \rangle$, (2) execution of partial spin swaps $U(\theta )=\cos (\theta /2)-i\sin (\theta /2)\times \,{\rm{S}}{\rm{W}}{\rm{A}}{\rm{P}}$, where SWAP acts between a pair of spins, and (3) pairwise measurement of singlet versus triplet (that is, $| \uparrow \uparrow \rangle ,| \uparrow \downarrow \rangle +| \downarrow \uparrow \rangle ,| \downarrow \downarrow \rangle$). All such interactions are available from direct-current or ‘baseband’ pulsing using the Pauli-spin blockade mechanism, which uses electrode voltages that push electrons closer to or farther from another. The resulting ‘exchange interaction’ reduces the energy of the singlet state relative to all triplet states owing to a combination of Coulomb repulsion and the Pauli-exclusion principle. No magnetic fields of any kind are required, and the entire spin system, barring errors, would maintain total spherical symmetry.

Qubits are formed from three spins in three dots using the symmetry-respecting encoding of a decoherence free subsystem^1–4^. The eight resultant spin states may be described in the basis denoted as $| {S}_{12},{S};{m}\rangle$ where *S*_12_ encodes the total spin angular momentum of the first two spins (*S*_12_ = 0, singlet; *S*_12 _= 1, triplet), *S* encodes the total spin of all three spins, and *m* encodes the spin projection. Ideal exchange-only initialization, logic and readout address only on the *S*_12_ degree of freedom, and *S* would remain, for all encoded qubits, with constant value *S* = 1/2. The projection *m* = ±1/2 is a random and inconsequential gauge degree of freedom. Global magnetic fields *B* impart global phases that are also inconsequential. The angles *θ* for each exchange ‘gate’ *U*(*θ*), which we call exchange angles or ‘exchangles’, are determined by the time integral of the exchange energy, and as such are calibrated against direct-current voltage pulses with no direct dependence on pulse shape. Accordingly, the control of this qubit can be realized by precise switching between pairs of voltage levels—highly reminiscent of digital logic—and so is amenable to the energy-efficient cryo-control system we have described.

The dominant qubit errors can be grouped into two categories: (1) miscalibration of exchangles *θ*, or (2) dot-level gradients of the magnetic field. In multiqubit operation, both error types cause some fraction of encoded error (that is, bit-flips and phase-flips on the *S*_12_ number) and some fraction of leakage error (that is, flips from *S *= 1/2 to *S* = 3/2). Quantitative modelling of these contributions, as well as the translation of physical processes into exchangle miscalibrations and magnetic gradients, is discussed in ‘Simulation’ in Supplementary Information. Exchange miscalibration can be incoherent (that is, dynamically fluctuating charge noise from the qubit chip, interconnect or controller) or coherent (that is, static or contextual miscalibration from imperfect signal generation or transmission); the last of these dominates in the demonstrations presented. Similarly, gradients of the magnetic field can arise from fluctuating hyperfine interactions with ^29^Si and ^73^Ge nuclear spins or from static magnetic screening and flux-trapping arising from local superconductivity. The former source of gradients is mitigated using isotopic enhancement, the limitations of which determine the ${T}_{2}^{* }$ values of approximately 20 μs (Fig. 3c). The latter source is mitigated by limiting the magnetic field of the device to be close to zero, for which we use current coils external to the fridge to vector-cancel Earth’s magnetic field. As such, all experiments described in this paper are at approximately zero magnetic field. Dephasing due to residual magnetic gradients of both types is reduced for idling qubits in our demonstrations using permutational dynamical decoupling. This permutes sets of three spins using SWAP operations *U*(π) in a way that causes local gradients across triple-dots to average to global fields impacting only the irrelevant gauge number *m* (ref. ^9^).

## Online content

Any methods, additional references, Nature Portfolio reporting summaries, source data, extended data, supplementary information, acknowledgements, peer review information; details of author contributions and competing interests; and statements of data and code availability are available at 10.1038/s41586-026-10754-7.

## Supplementary information

Supplementary Information Supplementary Sections 1–8, including Supplementary Figs. 1–53 and Supplementary Table 1 – see Contents for details.

Peer Review File

## Figures and Tables

**Extended Data Fig. 3:**
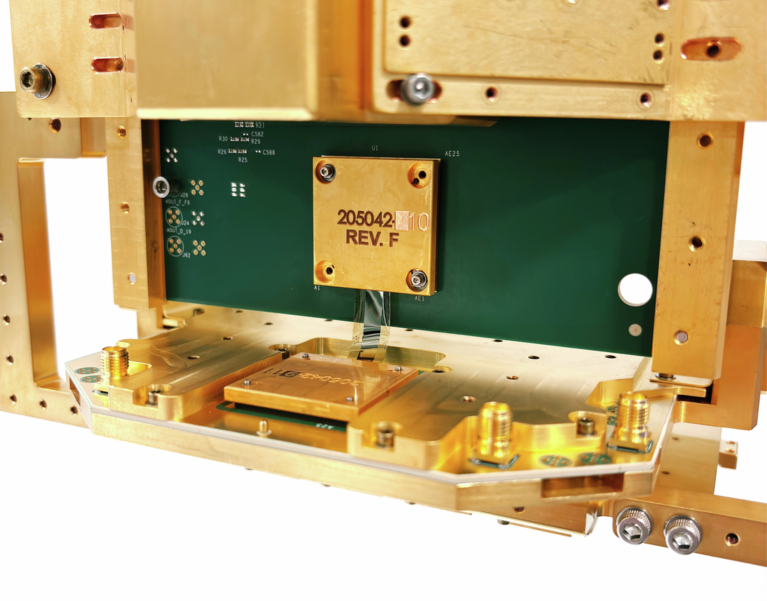
Back of Integrated QPU. The ribbon cable connects from the back of the motherboard at 4 K to the daughterboard at mK.

**Extended Data Fig. 5:**
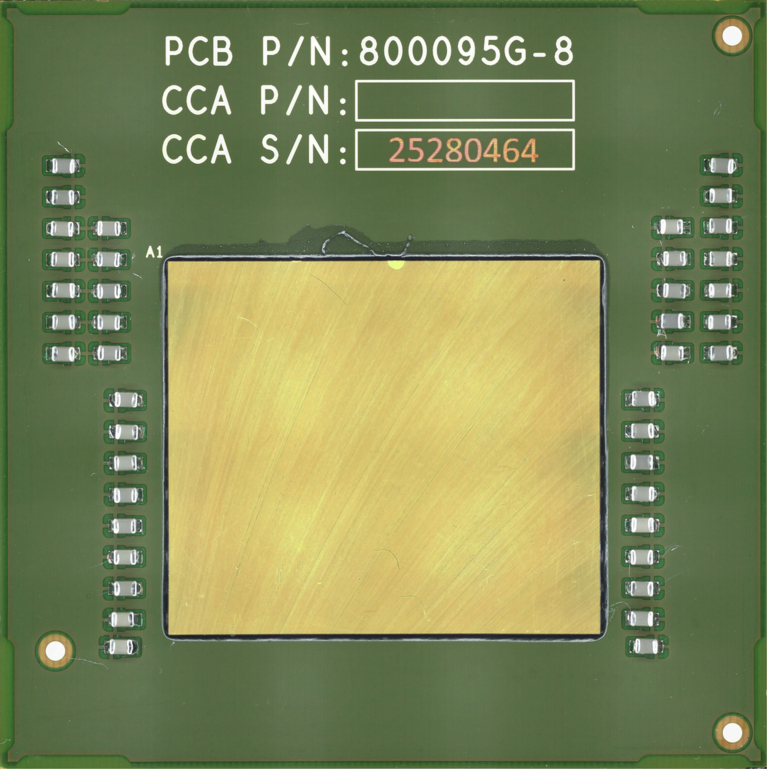
Photograph of a cryo-controller assembly. The bonded combination of CMOS die and high-density capacitor arrays is attached to a fine-pitch laminate LGA package. Additional surface-mount capacitors on the package improve the performance of the power distribution network.

## Data Availability

The data that support the findings of this study are available via Zenodo at 10.5281/zenodo.20358148 (ref. ^54^). The core algorithms and analysis methods are described in the Supplementary Information to enable independent reanalysis.
