# Supplementary material for: A digitally controlled silicon quantum processing unit
Source: Nature. 2026 Jul 29;655(8125):1154–9. doi: 10.1038/s41586-026-10754-7 (PMC13421337; doi:10.1038/s41586-026-10754-7)
Supplement: Supplementary file 1 — Supplementary Sections 1–8, including Supplementary Figs. 1–53 and Supplementary Table 1 – see Contents for details. [file 41586_2026_10754_MOESM1_ESM.pdf]

nature portfolio

https://doi.org/10.1038/s41586-026-10754-7

Supplementary information

A digitally controlled silicon quantum processing unit

In the format provided by the authors and unedited

A digitally controlled silicon quantum processing unit: Supplementary Information

Members of the HRL Quantum Team and Collaborators

CONTENTS

S1. Cryo-CMOS qubit controller 1
A. Digital architecture 3
B. Analog gate driver layer 3
C. Analog performance 5
D. Power consumption 6

S2. Superconducting interconnect 6
A. Fabrication 6
B. Packaging 7
C. Electrical performance 7
D. Thermal performance 7

S3. Environmental control 8
A. Thermal management 8
B. Qubit temperatures 8
C. Flex cable 8
D. 4 K transimpedance amplifier 10

S4. Software 11
A. Pulse sequence compilation 11
B. Circuit compilation 11
C. Constructing qubit operations 11
D. GAQQMap 13

S5. Tune-up and calibration 13
A. Parallelism 13
B. Neural nets for charge stability inference 15
C. Automation 15

S6. Qubit characterization 15
A. Initialization window widths 15
B. Device voltage state 15
C. N_{osc} methodology 15
D. Fingerprint compilation 17
E. Exchange calibration uniformity 18
F. Yield of devices 18
G. Valley splitting 18
H. Blind randomized benchmarking (BRB) 19
BRB implementation and analysis 20
Interleaved BRB 21
Random Clifford sampling 21

S7. Simulation 22
A. Coherent spin simulation 22
Spin state vector (SSV) 23
Matrix product state (MPS) 24
B. Interconnect simulation 25
Interconnect circuit model 25
V-J device model 25
Gate error predictions for ISI and crosstalk 26
C. Event simulation 28
D. Detector error models (DEMs) 28
E. Quasi-static miscalibration model 28

S8. Multiqubit experiments 30
A. Device layout and qubit orientation 30
Alternative readout configuration 30
Qubit orientations 30
B. [3,1,3] demonstration 30
[3,1,3] noise characterization 30
[3,1,3] error attribution 30
[3,1,3] DEM analysis 31
C. [5,1,5] demonstration 32
[5,1,5] noise characterization 33
Spikes in detection events 33
[5,1,5] DEM analysis 34
D. [[4,2,2]] demonstration 34
[[4,2,2]] noise characterization 35
[[4,2,2]] initialization and post-selection 38
[[4,2,2]] DEM analysis 39

References 40

S1. CRYO-CMOS QUBIT CONTROLLER

The cryo-CMOS qubit controller is a high-performance, mixed-signal system on chip fabricated in a commercial 130-nm RF CMOS technology. Circuit designs are optimized at the transistor level for cryogenic performance. The controller is designed to control an array of exchange-only silicon spin qubits from the 4 K stage of a commercial dilution refrigerator. It generates all time-varying voltage signals required to perform tune-up, state preparation and measurement (SPAM), and qubit state manipulation. The controller die contains 70 million transistors, measures 21×19 mm^2, and has 3054 C4-style pins arranged in a ground-signal/power-ground pattern with a minimum pad pitch of 250 μm. Figure S1 depicts a photograph of a die with the main sections of the design annotated and Fig. S2 shows a top-level block diagram of the controller.

The cryo-controller can source signals for up to 54 quantum dot ("P") gates, 66 exchange ("X" and "Y") gates, 12 tunnel ("T") gates, and 24 gates used for initialization and readout ("Z", "M", and "S"). There is a 1:1 mapping of 150 of 156 outputs to the physical qubit gates they control which are naturally arranged into "sectors" labeled A-F. A sector is the set of gates used to form 3 physical qubits (PQB) vertically aligned

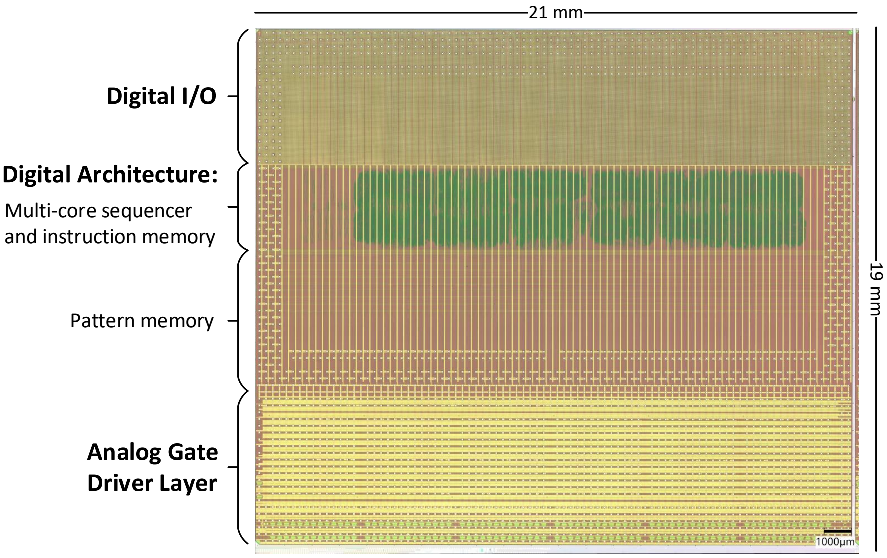

Supplementary Fig. S1. **False-color die photo of the cryo-CMOS qubit controller.**

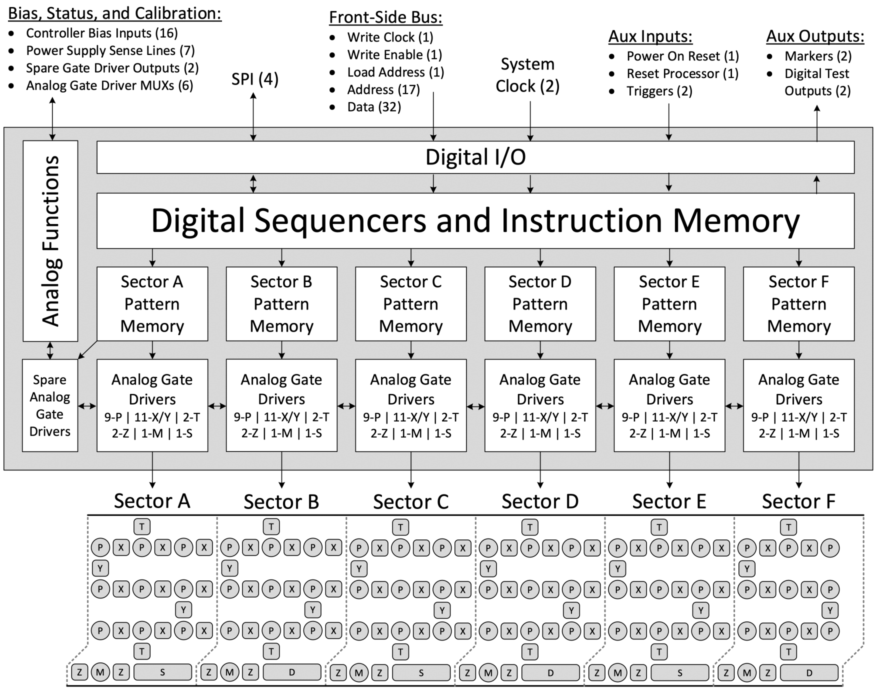

Supplementary Fig. S2. **Top-level block diagram of the cryo-CMOS qubit controller.**

in 3 rails of the device (9 P-gates, 11 X/Y-gates, 2 T-gates, and 4 SPAM gates per sector). To bias quiescent gates such as bath and field gates (not shown in Fig. S2), low noise voltages are routed directly to the qubit device from room temperature electronics. Three exchange gate outputs in Sector F (XF3, XF6, and XF9) are unused by the qubit and routed to diagnostic connectors on the motherboard, and 3 of 6 available S-gate outputs are not connected. The chip also includes two spare gate drivers routed out of the system for diagnostics or characterization. The controller only requires power, a handful of DC analog biases, and a manageable number of intermediate-speed lines for digital communication to be provided from room temperature.

A. Digital architecture

The cryo-controller's digital architecture consists of six independent instruction sequencers, a shared bank of instruction memory (6144 words, each 32 bits wide), and, for each gate driver output, a custom "pattern memory" register that stores up to 512 commands that are each 24 bits wide. The controller's digital interface consists of 66 signals: a 2-wire differential system clock, a 52-line front-side parallel bus (including write clock, address, and data), an industry-standard 4-wire serial peripheral interface (SPI), and 8 auxiliary inputs and outputs. The front-side bus transmits program data to instruction and pattern memories using the write clock and executes at the operating frequency of the system clock. The SPI is used for reading and writing a variety of configuration registers within the digital architecture and analog gate driver layer. The auxiliary I/O includes signals that may be used to interface with external readout electronics, inputs for real-time user-selectable instruction branching ("Triggers"), outputs for general purpose use ("Markers"), and specific digital diagnostic outputs ("Digital Test Outputs"). Any of the digital input signals can be multiplexed to these diagnostic outputs to be routed to room temperature. This provides the capability to perform in-situ loop-back signal connectivity testing within the dilution refrigerator.

The multi-sequencer core is responsible for providing a memory address to each pattern memory cell every clock cycle. The addresses sent to pattern memory are computed according to a customized qubit-control instruction set architecture (ISA). The ISA includes instructions for stepping, pausing, jumping, looping, branching, memory stack manipulation, toggling of external outputs, and more. Each sequencer has integrated pseudorandom number generators that can be accessed with specialized instructions for memory-efficient randomized benchmarking (see Sec. S6 H). There is also the capability to enforce inter-sequencer coordination using real-time interrupts and interrupt resolution logic.

Addresses computed by the sequencers are used to retrieve pattern memory words which are routed to each analog driver every clock cycle. Pattern memory words specify information such as DAC voltage, pulse duration, and other analog functionality that is decoded and executed by local digital modules within the gate driver blocks themselves. It is possible to configure the mapping of any pattern memory cell (and therefore any analog driver) to receive its address pointer from any sequencer using the SPI. This functionality provides an enormous degree of flexibility in assigning independent or coordinated control to arbitrary parts of the qubit device. This may be advantageous in particular for performing experiments on the qubit array in the presence of physical defects. Collectively, the cryo-controller's digital architecture is capable of autonomously executing effectively arbitrary-length randomized benchmarking sequences and complex multi-qubit experiments.

The cryo-controller contains several on-chip pseudorandom number generators. The output of each is a deterministic function of a user-defined seed. Each random number generator is a maximum length linear feedback shift register (LFSR) modified to shift out M pseudorandom bits in a single clock cycle. On-chip logic divides the set of 2^M possible bit-vector values into K equal sized subdivisions and selects the subdivision R that contains the random bit-vector. The resulting random integer R is uniformly distributed between 0 and K-1.

We implement randomized benchmarking (RB) with on-chip instructions that jump to an address + offset where the offset is the value of an on-chip random number. For 1-qubit RB we compile each of the 24 Clifford gates into subroutines with the first instruction of each subroutine located in a single software-defined jump table. The first instruction of each subroutine jumps out of the table, and the final instruction of each subroutine is the instruction to jump to address + offset where the address is the start of the jump table and the offset is a random number between 0 and 23. Additionally, we load an on-chip register with the desired RB sequence length and decrement this register on completion of each Clifford gate. When this register is decremented to zero, the jump to address + offset behavior is modified to jump to the first address past the jump table where we load the first instruction for the inverse Clifford gate subroutine. Calculation of the inverse Clifford gate is determined during program compilation by simulating the pseudorandom number generation process using the same seed value. We execute more complex RB protocols (2-qubit RB, interleaved RB, etc.) with a similar procedure utilizing multiple different sized random numbers and software-defined jump tables (see Sec. S6 H).

B. Analog gate driver layer

The "sector" is a natural way to organize both the qubit and the cryo-controller's analog gate driver layer. A block diagram showing this organization for the controller in detail is shown in Fig. S3. The fundamental analog driver building block is called the "DNX" dual gate driver which controls one P, M, Z, or S-gate and one X, Y, or T-gate. There are 3 variants of this building block ("D6X", "D5X", and "D3X") where "N" equals the number of DACs in the block, determined by the number of unique high-precision voltage levels required to control the gate pair. The "X" in the name denotes an exchange pulse generator. To control a sector of qubit gates, 13 DNX blocks are integrated together along with driver biasing circuitry. The gate driver sector is replicated 6 times to form the cryo-controller's full analog layer consisting of 78 DNX blocks containing a total of 366 DACs and 78 pulse generators.

Device tune-up and SPAM are performed by changing DAC output voltages. The default DAC bandwidth is about 10 MHz, which balances SPAM switching speed ($T_{10\%\text{-}90\%} \approx 35$ ns) and output noise for exchange. DACs can dynamically change their switching speed in order to generate both accelerated ($T_{10\%\text{-}90\%} \approx 10$ ns) transitions as well as slow, pseudo-linear ramps with tightly-controlled slew rates intended for traversing

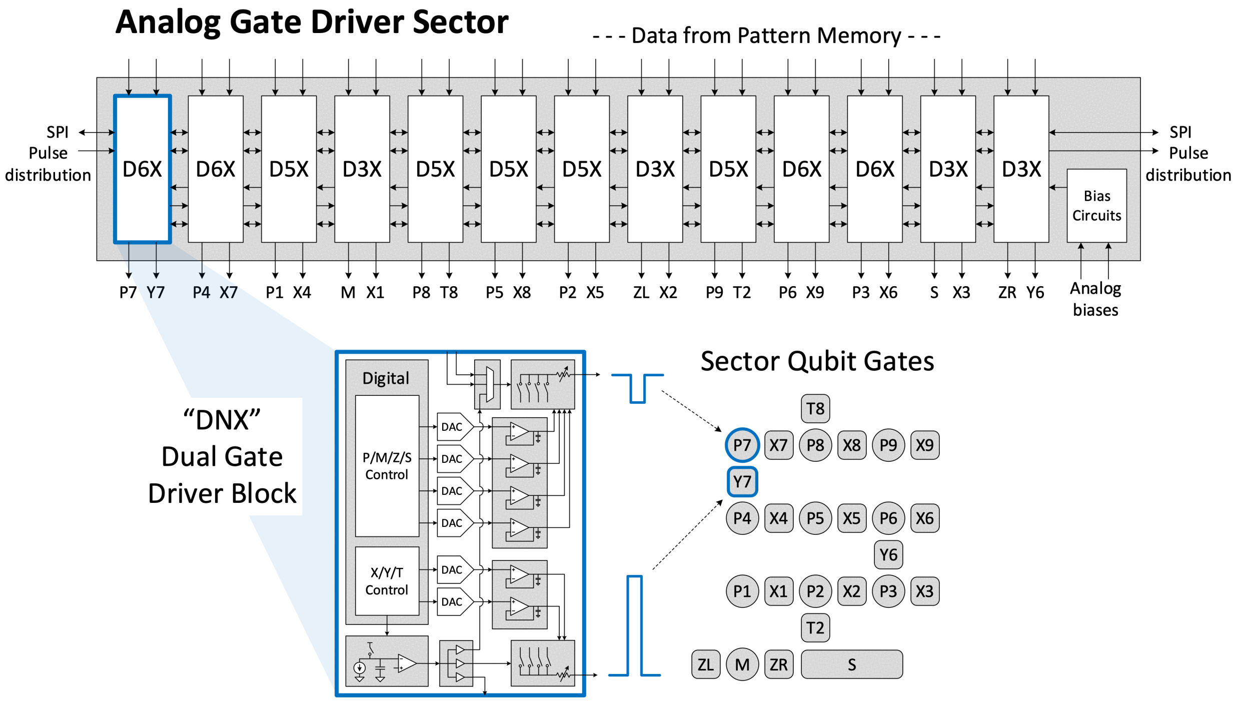

Supplementary Fig. S3. **Block diagram of an analog gate driver sector and the variations of gate driver blocks within it.** The gate driver layer contains six sectors.

charge transitions (the "tie bar") during qubit initialization.

GHz-bandwidth, time-modulated exchange gate pulses are generated for qubit state manipulation by switching between fixed, buffered DAC values. The precise timing information comes from the pulse generator, a high-resolution digital-to-time converter (DTC). The exchange-gate driver's local digital module receives a digital input word from pattern memory and converts a portion of it into a variable-width, logic-level pulse by the DTC. The logic-level pulse is translated into an output pulse between the appropriate voltage levels with a high-speed output switch and associated switch driver. Each exchange gate driver's pulse generator output is distributed to neighboring dot gate switch drivers with low timing skew to form time-aligned, triplicate pulse waveforms for symmetric exchange operation [1].

Qubit gates act like open circuits (i.e. are high impedance), so any pulse sent from the cryo-controller is sent back as a near-perfect reflection that must be sufficiently attenuated before the next pulse is launched. Failure to do so results in inter-symbol interference (ISI) error. To this end, the gate driver's output impedance is designed to match the nominal 50 Ω characteristic impedance of the interconnect in the system. The output switch features configurable resistance control to tune the termination to 50 ± 1 Ω using the SPI. The parasitic capacitance of the output is minimized to provide at least 20 dB return loss up to the 500 MHz analog bandwidth of instrumentation. The output switch also features a configurable switched-capacitor output pulse filter to help mitigate reflections. An adjustable capacitor is connected to the output for the duration of a pulse to add to its rise and fall times by up to 400 ps, and it is disconnected before the reflected pulse arrives to avoid degrading the quality of the output termination.

DACs are buffered with unity-gain voltage amplifiers that provide approximately 60 MHz bandwidth for restoring the exchange and compensation pulse voltages to their calibrated values in between pulses in a sequence. The buffer amplifier is designed in conjunction with a sizable off-chip load capacitor that provides the initial charge needed to form high-speed pulse rising and falling edges. The buffer limits voltage droop on the pulse amplitude for the duration of the pulse and restores the charge on the output capacitor for the next pulse. These charge reservoir and stabilization capacitors are fabricated as custom, high-density flip-chip capacitor arrays that are bonded directly to the CMOS die to minimize parasitic inductance. Flip-chip capacitors that are not mapped to buffer amplifier outputs are connected to the analog power supply rails to lower the impedance and increase the effectiveness of the power delivery network (PDN).

Figure S4 summarizes the analog theory of operation and illustrates how the gate driver circuitry executes tune-up, SPAM, and exchange pulsing operations. Each of the six gate driver sectors also features a multiplexing network to connect any constituent gate driver output to a dedicated output pin. These six outputs in the analog layer are routed to room temperature for in-situ functionality screening and DAC code-to-voltage calibration. Pulse generator codes are calibrated to specific

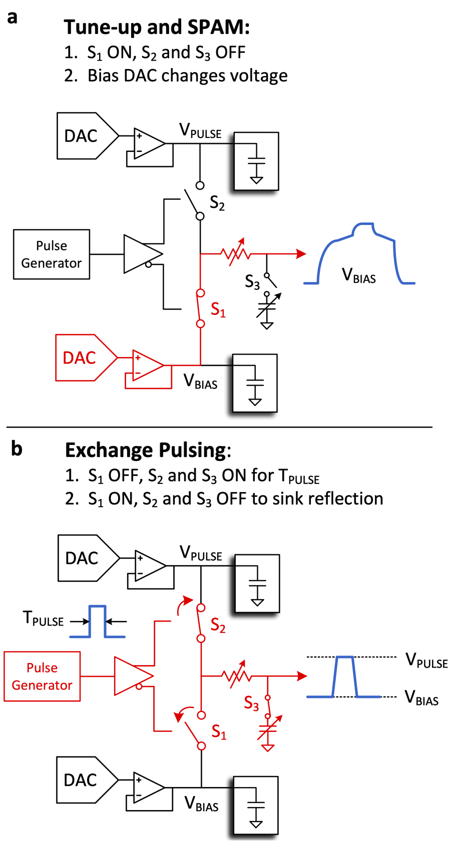

Supplementary Fig. S4. **Illustrations of how the analog gate driver executes: a,** tune-up and SPAM, and **b,** exchange pulsing operations.

qubit rotation angles. This is accomplished by first acquiring a high-resolution code-to-pulse width transfer function from one of the three available exchange gate outputs in sector F not connected to the qubit device. That transfer function is then used to generate finely-tuned code-to-angle calibrations for all other pulse generators on the chip. Example code-to-voltage and code-to-time curves and how they are related to voltage pulse waveforms are shown in Fig. S5.

C. Analog performance

DACs are programmable from 0 to 1 V with an RMS step size less than 10 μV and are designed together with the buffer amplifiers to be low noise. Low frequency "flicker" noise is typically a significant contributor in baseband circuits and becomes a significant contributor to qubit error if it is comparable to the intrinsic low frequency charge noise of the qubits. This section presents representative performance characterization data at 4 K from a typical gate driver output routed to room temperature instrumentation. Figure S6 plots the step-size from a 200,000-point, sorted DAC-code-to-voltage transfer function spanning the full output voltage range. Figure S7 plots the noise density at 1 Hz as a function of set voltage and the results show it is less than 1.4 $\mu V/\sqrt{Hz}$.

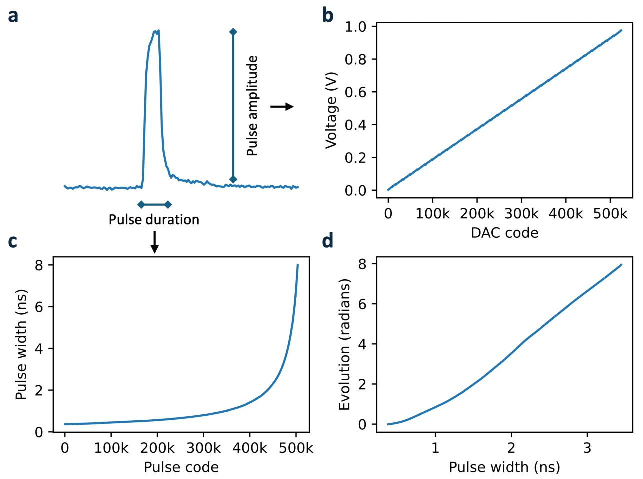

Supplementary Fig. S5. **Pulse waveform and calibration curves. a,** An example voltage pulse waveform. The duration of this pulse is set by a code-to-time word and the amplitude by the difference of two DACs (low and high voltage) configured with code-to-voltage words. **b,** DAC code-to-voltage transfer function. **c,** Pulse code-to-time transfer function. **d,** Typical pulse duration to exchange angle qubit calibration curve.

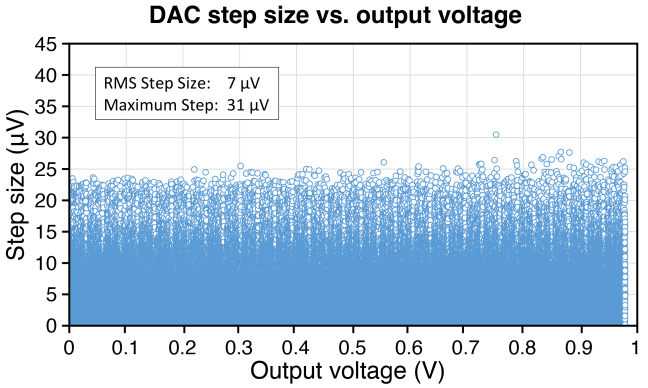

Supplementary Fig. S6. **Data showing DACs are programmable from 0 to 1 V with an RMS step size less than 10 μV.**

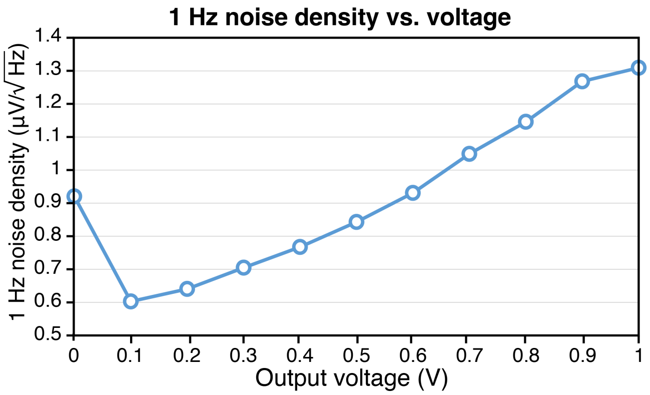

Supplementary Fig. S7. **Data showing the typical driver's output noise density at 1 Hz is less than 1.4** $\mu V/\sqrt{Hz}$**.**

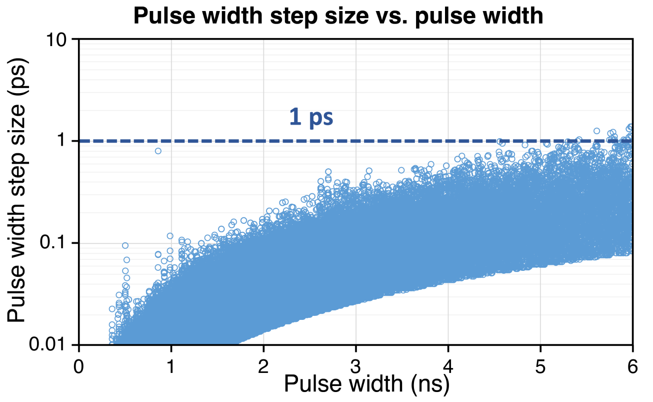

Supplementary Fig. S8. **Data showing the pulse generator step size is mostly under 1 ps from 0.4 to 6 ns.**

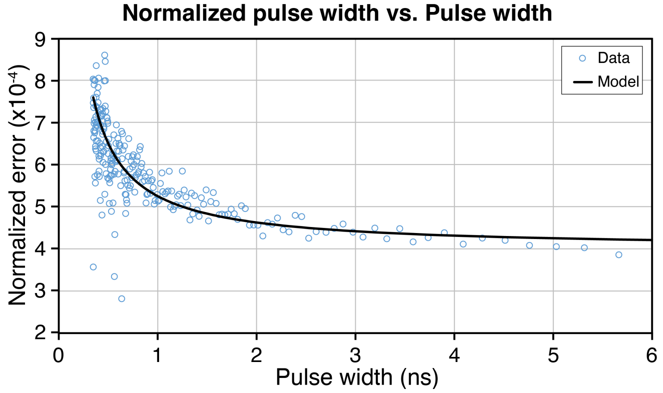

Supplementary Fig. S9. **Measurements and model fit of normalized pulse width (PW) jitter from 0.4 to 6 ns.** The model fit function is 125 fs/PW + $4{\times}10^{-4}$.

The pulse generator is designed to produce performant pulses at a maximum pulse repetition frequency of 41.67 MHz. Pulse width is tunable from 0.4 to 6 ns with approximately picosecond resolution across the full range and with sub-picosecond resolution below 4 ns. Figure S8 plots measurements of the output pulse width step-size as a function of set pulse width. Figure S9 plots the pulse width jitter expressed as a normalized error (jitter divided by pulse width) across the full pulse width range. Jitter error is $7.5{\times}10^{-4}$ for the shortest pulses and decreases to $4{\times}10^{-4}$ for longer pulses. These datasets were taken through 10 feet of high-bandwidth coaxial cabling into a 256 Gsps oscilloscope.

D. Power consumption

The cryo-controller requires one digital power supply and six analog power supplies from room temperature. Power consumed by the digital architecture is dynamic power and is therefore a strong function of the operating frequency and the complexity of the program. The analog gate driver layer

Cryogenic Controller Power: Single-core, single-qubit RB at 250 MHz	
**Component**	**Power (W)**
Digital	0.788
Pulse Generators	0.575
DACs	0.760
Buffers	0.523
Switches	0.175
**Total**	**2.821**

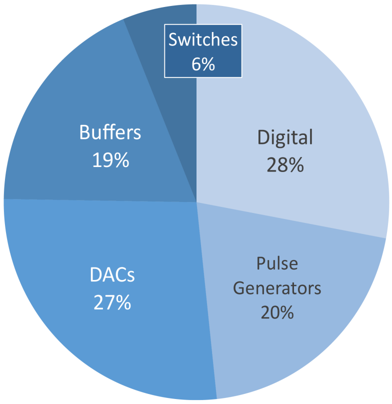

Supplementary Fig. S10. **Detailed breakdown of cryo-controller power consumption during an optimized single-sequencer, single-qubit RB experiment at 250 MHz.**

power consumption is mostly static because the typical duty cycle for pulsed outputs during an experiment is less than 10%. Empirical data suggests randomized benchmarking consumes more digital power than the other kinds of experiments discussed in this publication. For an optimized single-sequencer, single-qubit RB experiment at 250 MHz, the chip consumes approximately 2.8 W. A detailed power breakdown of this scenario is shown in Fig. S10. It is estimated that power consumption would increase up to 3.5 W if all six sequencers were independently running randomized benchmarking.

The total power consumption is within the typically-available thermalization budget of the 4 K stage in commercial dilution refrigerators. The controller features granular power control such that most digital assets and analog sub-circuits can be powered down when not in use. Approaches to substantially reducing the power-per-qubit through architecture and scaling have been identified, but were not required for this work.

S2. SUPERCONDUCTING INTERCONNECT

A. Fabrication

The superconducting ribbon cables are fabricated on 150 mm silicon wafers using Nb for the superconducting layers and polyimide for the dielectric. Polyimide layers serve as both low-k interlayer dielectric (ILD) and in providing structural support for the ribbon cable. Three levels of Nb metal realize a lower ground plane, a signal layer, and an upper ground plane to create a fully-enclosed stripline environment. Via trenches

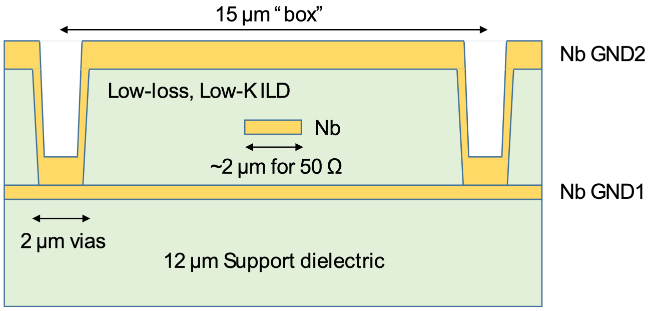

Supplementary Fig. S11. **Schematic cross-section of a single trace in the ribbon cable.** All metal layers are Nb and the ILDs are polyimide.

are formed between each signal trace, electrically connecting the upper and lower ground planes. The resulting structure (Fig. S11) is a rectangular coaxial structure with micron-scale signal wires on a 15 μm pitch. Pads are finished with Au to support assembly operations. Typical cable designs are 6 cm long, approximately 1 cm wide, and implement 200 to 500 independent signal wires. High-yielding wafer-scale processes are able to routinely realize devices with 100% interconnect yield for these 6 cm^2 devices.

B. Packaging

The ribbon cables are indium bonded to ten-layer laminate package substrates with length matching within related wire bundles. The interface ends of the ribbon are capillary-underfilled to enhance the mechanical strength of the bond and provide environmental protection to the joints. The package mates to the printed circuit board (PCB) using a land-grid spring-probe array with an 800 μm pitch. The pads on the package and PCB are gold plated. The package-to-PCB interface has undergone over 10 remate cycles on multiple test systems with no loss of functionality. A photograph of the assembly is in Extended Data Fig. 3.

C. Electrical performance

The RF performance of the ribbon cable was measured at 4 K to determine insertion loss, impedance, and cross-talk. Figure S12 shows a typical insertion loss (S_{21}) measurement for a single transmission line in the cable. The de-embedded cable loss is approximately −1.0 dB/GHz/m. Figure S13 shows a single transmission line impedance with 50 ± 5 Ω being typical for all wires within a cable. Cross-talk between nearest neighbor wires within the cable is < −90 dB at 1 GHz and < −80 dB at 10 GHz. Wire critical currents are approximately 150 mA. All cables are screened electrically inline on the wafer and at 300 K and 4 K after packaging.

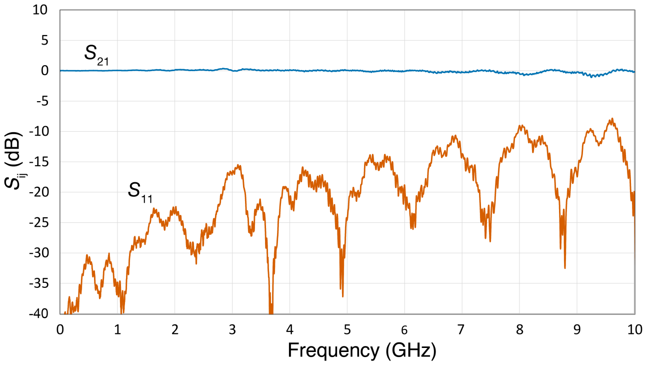

Supplementary Fig. S12. **6 cm ribbon cable insertion loss at 4 K.** Actual ribbon cable insertion loss (S_{21}) and return loss (S_{11}) are de-embedded from the non-superconducting test fixturing using *in situ* cryo calibration standards measured at the end of the cryo test fixturing. Imperfections in the fixturing and de-embedding process leave some residual ringing which causes minor artificial non-passivity at some frequencies. A fit of the de-embedded data yields a loss of approximately −1.0 dB/GHz/m.

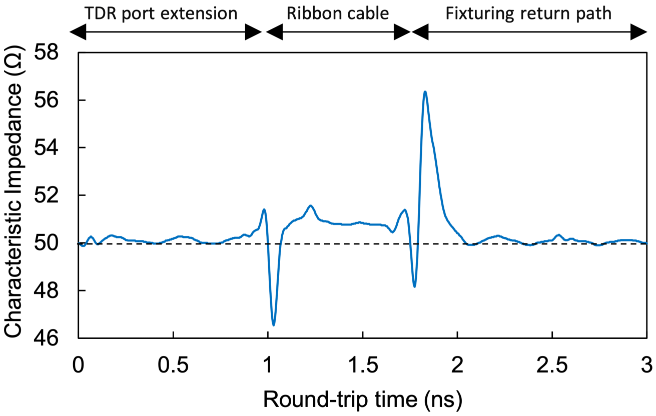

Supplementary Fig. S13. **Time-domain reflection response of 6 cm ribbon cable at 4 K.** De-embedded S_{11} data has been transformed to yield a characteristic impedance profile for the ribbon cable assuming a 50 ps rising edge. Between the capacitive drops at the interfaces on either end of the cable associated with the bond pads (at 1.03 and 1.76 ns), the characteristic impedance averages 50.8 Ω. The initial 1 ns is a time-domain reflectometer (TDR) port extension, an artificially calculated response prior to the device under test, and is associated with the time-domain transformation technique.

D. Thermal performance

The heat leak through the cable from 4 K to about 50 mK is approximately 10 μW. The cables have survived over 20 thermal cycles across multiple test systems with no signs of fatigue or degradation.

S3. ENVIRONMENTAL CONTROL

A. Thermal management

High-fidelity state preparation and measurement of our exchange-only spin qubit processor requires operation at ultra-low cryogenic temperatures. To accomplish this, we utilize a commercial Bluefors XLD1000 dilution refrigerator, capable of rejecting 1 mW of heat at 100 mK. Cooling power at 4 K is provided by three Cryomech PT-425 pulse tube coolers: one dedicated for pre-cooling the circulating ^{3}He/^{4}He mixture and two providing a nominal 5 W of cooling power to the 4 K plate of the cryostat. This 4-Kelvin cooling capacity supports the heat loads from cabling as well as the cryo-controller (Sec. S1 D). The CMOS-generated control signals are routed to the qubit chip via a 296-trace superconducting Nb/Polyimide ribbon cable (Sec. S2) that launches off a printed circuit board (PCB) which houses the cryo-controller at the 4 K stage and lands on a separate PCB that houses the qubit chip at the mK stage.

The signals to support the qubit chip and cryo-controller are binned into several functional roles, outlined in Fig. 1 of the main text, each having their own electrical requirements. Digital and analog power rails for the cryo-controller, as well as readout amplifier power, are delivered with individual coaxial cables to minimize IR drops and quiescent power dissipation to the 4 K stage. The signals supporting the cryo-controller digital I/O and reference voltages (along with a select set of DC qubit chip biases) are routed from room temperature to the 4 K stage of the cryostat via two chains of custom, 100-trace flexible cryogenic cables (see Sec. S3 C). The thermal loads at the 50 K and 4 K stages of the dilution refrigerator are minimized by appropriate choice of material according to the requisite signal bandwidth: DC references and qubit chip static biases are routed through a higher resistance CuNi/CuNi (signal/ground) cable chain with 10 MHz of bandwidth, while the high-speed digital I/O is routed through a hybrid Cu/CuNi-Cu/Cu cable chain with a combined bandwidth of 450 MHz. We estimate the total conductive heat load to 4 K from all room temperature signals to be less than 200 mW, consistent with measured data shown in Fig. S14a.

The analog and digital power dissipation of the cryo-controller itself dominates the parasitic loads discussed above, and is estimated at less than 3.5 W (Sec. S1 D). Close proximity of the cryo-controller to the qubit chip in our architecture improves signal integrity but also necessitates effective thermal isolation of the two components. In order to avoid thermal gradients across the motherboard exceeding the superconducting critical temperature (8.5 K) of the mK interconnect, the chip die is directly heat sunk to the 4 K plate through a copper framework. Based on sweeps of directly applied 4 K heat while monitoring interconnect trace resistance, we estimate approximately 6 W of cooling power available for cryo-control at 4 K in the current design. The combined heat leak to the qubit chip from amplifier cabling and the Nb interconnect is measured to be less than 20 μW, as shown in Fig. S14a, enabling mixing chamber temperatures as low as 20 mK or less in a fully integrated system.

B. Qubit temperatures

In general, the phonon temperature of the cryostat represents only a lower bound on the effective qubit temperature relevant to a quantum circuit, which may be significantly higher due to thermal interface resistances and electrical noise contributions. We directly verify low qubit temperatures by measuring the width of a quantum dot charge number transition as a function of applied plunger gate voltage and fit the data to a characteristic form as in Ref. [2]. With this method we find an average electron temperature of $\overline{T}_{\mathrm{e}} = 150$ mK across an entire device based on the $N = 0$ to $N = 1$ transition of the quantum dot closest to the 2DEG bath gate in each sector, as shown in Fig. S14b. This compares favorably to electron temperatures reported from our previous system architecture utilizing all-room-temperature control [3].

We also perform low-frequency charge noise spectroscopy on the same qubit chip by measuring the power spectral density of current fluctuations through a readout dot-charge-sensor (DCS) while biased to maximum conductance (peak), minimum conductance (tail), and maximum transconductance (side). Taking linear combinations of the resulting spectral densities S_i, we can extract an "effective plunger gate voltage noise" S_{g}:

$$S_{\mathrm{g}} = \frac{1}{G^2_{m,\mathrm{side}}}\left(S_{\mathrm{side}} - S_{\mathrm{tail}} - \left(\frac{G^2_{\mathrm{side}}}{G^2_{\mathrm{peak}}}\right)\left\{S_{\mathrm{peak}} - S_{\mathrm{tail}}\right\}\right), \quad \text{(S1)}$$

where G_x are the DCS conductances, and G_m is the transconductance. The experimentally measured plunger-gate-referred RMS fluctuation, shown in Fig. S14c, is found to be under 10 μV in the frequency range of 20 Hz to 7 kHz, demonstrating minimal coupling of 60 Hz mains or other low-frequency noise sources through the cryo-controller/qubit chip interconnect, though this width is still believed to be dominated by system noise rather than intrinsic noise.

C. Flex cable

We implement our cryogenic interconnect from room temperature to 4 K using a series of flexible cables (Fig. S15) manufactured using standard flex PCB processes. The cables are comprised of six metal layers designed in a stacked stripline configuration, with the two stripline layers joined by an adhesive. Signal traces are etched from a metal layer and vias are used to form electrical connections to the signal traces, as well as to join the GND planes together to prevent unwanted resonant modes from forming during the transmission of high-speed electrical signals.

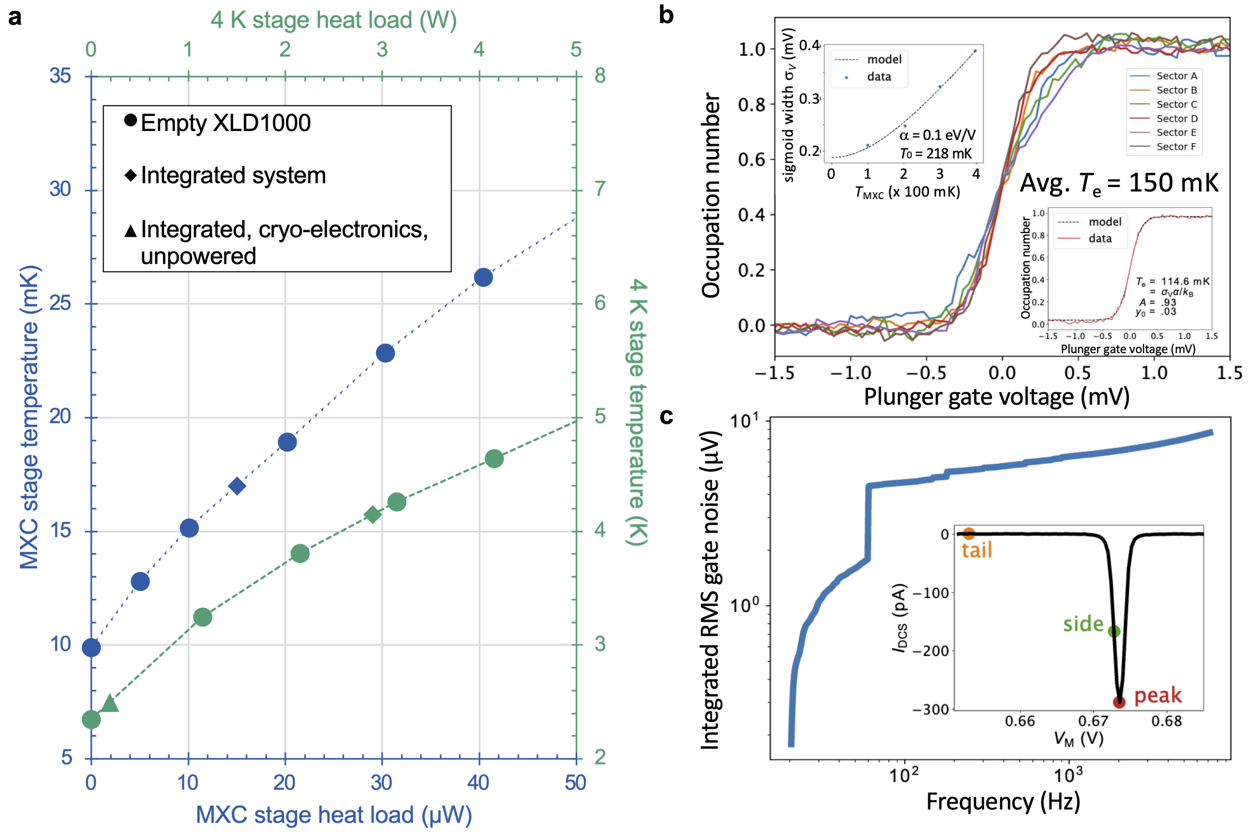

Supplementary Fig. S14. **QPU thermal and electrical environment. a,** Cooling power data (circles) measured in two bare XLD1000 dilution refrigerator cryostats before integrating cabling and control electronics. Markers (diamonds) denote the steady-state temperature of both stages after each cryostat is fully integrated with control hardware, and power is supplied to cryogenic electronics. An additional marker (triangle) shows the 4K stage temperature of an integrated system with cryogenic electronics deliberately unpowered. Referring these temperatures to the cooling power data of the base cryostats implies less than 200 mW of conductive cabling heat load, and less than 3 W of cryo-control power dissipation at the 4 K stage. **b,** Charge loading measurements for one quantum dot in each of the 6 sectors across a 54-dot qubit chip indicating an average electron temperature of 150 mK, assuming a nominal gate lever arm α of 0.1 eV/V. All curves are normalized according to the DCS current measured deep in the $(N = 0)$ and $(N = 1)$ charge cells respectively. A voltage width σ_V is extracted from each curve by first subtracting the linear baseline offset from capacitive coupling of the plunger gate to the DCS, and then fitting to a characteristic sigmoid form {model: $y_0 + A[1 + \exp(V_\mathrm{p} - V_\mathrm{p}^0)/\sigma_V]$}, illustrated for one curve in the lower right inset. The nominal lever arm in this architecture is estimated independently via measurements of σ_V at high mixing chamber temperatures for select devices, with an example measurement from a separate device in the same lot shown in the upper left inset [model: $\sigma_V = (k_\mathrm{B}/\alpha)(T_\mathrm{MXC}^2 + T_\mathrm{e}^2)^{1/2}$]. **c,** The effective RMS gate voltage fluctuation, defined as the square root of the integral of the gate voltage noise spectral density $S_\mathrm{g}(f)$, measured with the same qubit chip as in **b**. The RMS fluctuation is found to be under 10 μV over the integration range of 20 Hz to 7 kHz. The inset shows the measured current through the DCS as a function of the M-dot plunger bias, as well as the three biases used to calculate $S_\mathrm{g}(f)$ as discussed in the text.

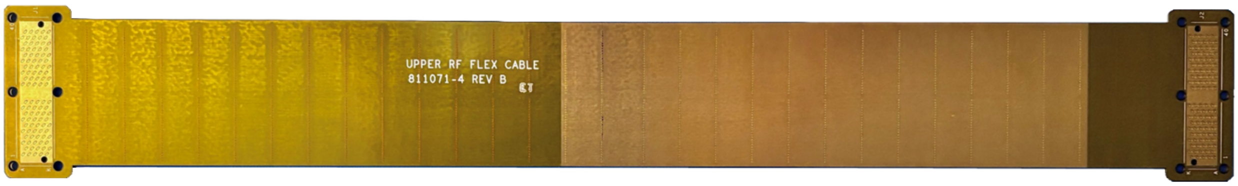

Supplementary Fig. S15. **One of the flex cables that together carry signals between room temperature and 4 K.**

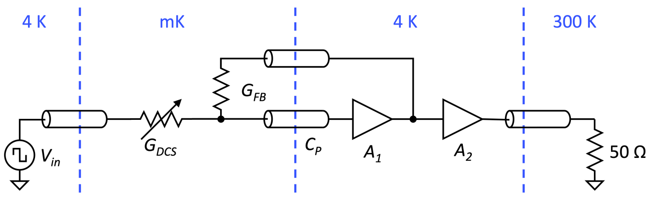

Supplementary Fig. S16. **Simplified electrical circuit of the custom 4 K TIA.** Note this design requires two connections between mK and 4 K per channel.

Using a combination of CuNi for ground planes and either Cu (for high speed) or CuNi (for low speed) for signal traces maximizes the signal bandwidth while minimizing the passive thermal load. We form a cable-to-board connection using Samtec ZRAY spring interposer arrays to form a mezzanine connection between an array of pads on the ends of the flex cables and a matching array of pads on the corresponding PCB. Cable thermalization to 50 K and 4 K stage plates are accomplished by means of clamping the cables with a flat plate onto areas of the cables with exposed GND metal. These are then clamped to an Au plated Cu cold finger attached to a stage plate within the dilution refrigerator.

For the vacuum feedthrough at the top of the fridge, we epoxy a PCB into a vacuum flange with an appropriately milled channel. The PCB has a ZRAY connection internal to the cryostat and uses Samtec HLCD microcoaxial cable on the external side to connect to instruments at room temperature by means of an appropriate breakout PCB. We externally verify the resistance and isolation of each trace of each cable using an automated wire harness tester. Once installed into the cryostat, we again verify the electrical continuity and isolation of the composite signal chain using an automated wire harness tester while the cryostat is both warm/vented and cold/evacuated using a loopback PCB at the end of the signal chain.

D. 4 K transimpedance amplifier

Measurement is accomplished by applying a square wave voltage to a variable conductance dot-charge-sensor G_{DCS}, as in Ref. [3], and measuring the associated current into a custom low-noise transimpedance amplifier (TIA) based on the model ATF-38143 high-electron-mobility transistor (HEMT). A simplified effective circuit of the TIA is shown in Fig. S16. The transimpedance architecture creates an amplifier input admittance for which $A_1 G_{\mathrm{FB}}$ in parallel with $j\omega C_p$ is much greater than G_{DCS}, where (per Fig. S16) A_1 is the gain of the first stage amplifier, G_{FB} is the conductance of the feedback resistor, and C_p is the total parasitic capacitance to ground between the DCS and HEMT gate. The resulting signal bandwidth and associated settle time is approximately 10 MHz. We model the amplifier input current noise by including the effects of HEMT voltage noise, thermal Johnson noise, and shot noise

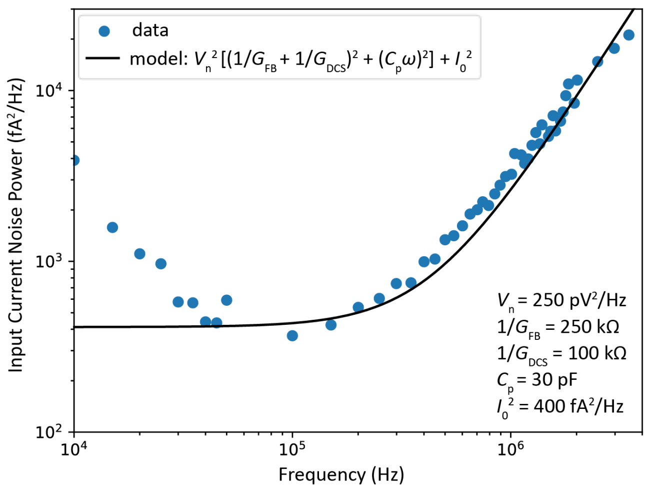

Supplementary Fig. S17. **Transimpedance amplifier input current noise power versus frequency.** At high frequencies f the f^2 term proportional to the parasitic input capacitance dominates the modeled noise spectrum (black line) and describes experimental data well (blue circles). Independent estimates of V_{n}, G_{FB}, G_{DCS}, and C_{p} are shown in the bottom right, while the white noise term I_0, set by the approximately 100 pA measured HEMT gate-current shot noise, resistor thermal noise, and potentially uninvestigated noise channels, was left a free parameter. At low frequencies, higher-order noise channels not included in this model begin to contribute, e.g. series capacitances multiplying the HEMT gate current. Maximum readout signal-to-noise ratio (SNR) is achieved by centering the fundamental modulation frequency around 100 to 200 kHz, where it matches the observed input noise minimum, though this is slower than our typical measurement cadence which must respond to other requirements.

according to

$$I_n^2 \approx V_n^2\left((G_{\mathrm{DCS}} + G_{\mathrm{FB}})^2 + \omega^2 C_p^2\right) + 4k_{\mathrm{B}}T_{\mathrm{mK}}\left(G_{\mathrm{DCS}} + G_{\mathrm{FB}}\right) + 2qI_G, \quad \text{(S2)}$$

where $V_n \approx 250\ \mathrm{pV}/\sqrt{\mathrm{Hz}}$ is the input referred voltage noise of the HEMT and I_G is the gate current of the HEMT. Figure S17 shows a comparison of this model to measured amplifier current noise data. Further noise reduction and increased bandwidth can be accomplished by using a lower noise HEMT and reducing C_p.

Overall qubit measurement performance of the integrated QPU is somewhat inferior to that reported in Ref. [3], with a full measurement duration of roughly 25 µs for the [5,1,5] measurements. This duration is "complete" in that it includes integration time as well as the transitions into and out of Pauli spin blockade and dynamical decoupling on spectator qubits, and no reference measurement is used. The performance is somewhat degraded by elevated parasitic capacitance and in-line resistance in the 18 qubit system, both of which have straightforward mitigations for future hardware revisions. It is additionally degraded by elevated low-frequency noise which we have traced to non-idealities in the system-wide power delivery network. The measurement SNR is minimally impacted by parallelism. The duration used for initialization in

the [5,1,5] measurements was roughly 5 μs, also inferior to that reported in Ref. [3]. The measurement was not as optimized as in Ref. [3], and we believe that similar optimization could have reduced the measurement duration to below 1 μs without significant change of fidelity, inclusive of charge-state transitions and dynamical decoupling.

S4. SOFTWARE

All qubit experiments in this work were performed using our custom "Quiver" control software. Quiver has numerous features and integrations to support device operation including, but not limited to, data collection and pulse sequence compilation.

A. Pulse sequence compilation

Qubit experiments require pulse sequences that are too complex for a human to generate manually. We use our custom domain-specific language, "PulseScript," along with associated software tools to define, manipulate, and compile voltage-time waveforms, i.e. pulse sequences. PulseScript contains several high-level language features for programming pulse sequences, such as subroutines, loops, parallelism, synchronization, and native support for sweeping parameters at runtime. While PulseScript programs can be written directly, we typically use Python bindings for programmatically generating PulseScript programs for device tune-up, calibration, benchmarking, etc.

The PulseScript compiler targets multiple backends while maintaining strict timing of the input program. The primary backend targets the cryo-controller, with additional backends for the cryoMUX systems (see Sec. S6 C) and to generate metadata for qubit simulation and debugging. Several optimization passes are performed to minimize the size of the compiled program, which is particularly important given the memory constraints of the cryo-controller (Sec. S1 A).

B. Circuit compilation

We use a custom structured format called "Isthmus," along with associated software tools, for defining and manipulating quantum circuit programs. Isthmus contains several semantic constructs for programming quantum circuits such as qubits, gates, exchange angles ("exchangles"), initializations, measurements, subroutines, loops, etc. We programmatically generate parameterized circuit programs for the specific qubit layout which has been tuned up on the array, ensuring operations are parallelized when possible. Generated programs are then translated by expanding qubit gates into sequences of exchange pulses using a database of predefined constructions, preserving parallelism and SPAM operations. Dynamical decoupling exchange pulses are woven into gate definitions on

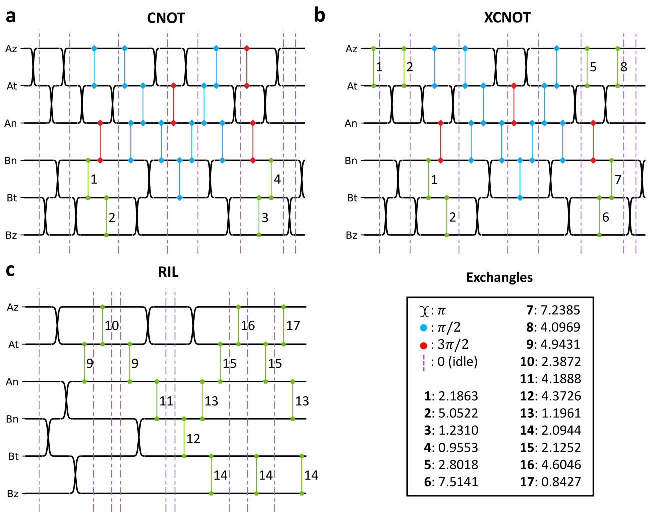

Supplementary Fig. S18. **Exchange-angle ("exchangle") implementations of two-qubit operations.** Each horizontal line represents a spin, with vertical connections representing exchange pulses. π-pulses are denoted as crossing lines, to emphasize their equivalence to spin-level swaps. "Ax" and "Bx" labels represent the spins in encoded qubits. Within each qubit, x can signify "z", the outermost spin in the initialization pair, "t" the center spin, or "n" the third spin. Exchanges of $\pi/2$ (blue) and $3\pi/2$ (red) are also common in entangling gates. Other exchanges are shown in green, and their numeric values, in radians, are given in the key. To mitigate calibration issues at small exchanges θ_j, pulses number 6 and 7 use exchanges $2\pi+\theta_j$. Idle timesteps (purple dashed lines) do not affect the functionality of the operation, but they enforce a regular cadence that mitigates some types of error. These implementations of **a,** CNOT and **b,** XCNOT differ in only six places, because they were derived from the same Fong-Wandzura CZ [4]; other sequences, such as **c,** RIL can show larger differences. These implementations are for an n-n coupling between encoded qubits. Slightly modified versions are used for z-z coupling.

any qubits not undergoing exchange rotations or SPAM. Care is taken with respect to spacing between pulses on same and adjacent axes to reduce the effects of inter-symbol interference. The resulting exchange pulse and SPAM program is translated to PulseScript by querying exchange and SPAM calibration parameters from the target system. Compilation continues from here utilizing the previously described pulse-sequence compilation process.

C. Constructing qubit operations

The above compilation process relies on a pre-defined database of operations, mapping qubit-level instructions such as H or CNOT into their implementations as sequences of exchange pulses. Some sequences, such as SWAP or single-qubit Cliffords, are simple enough for implementations to be analytically derived. Other two-qubit operations are generally more complicated, and we employ a variety of analytic and numeric techniques to add new sequences to the library.

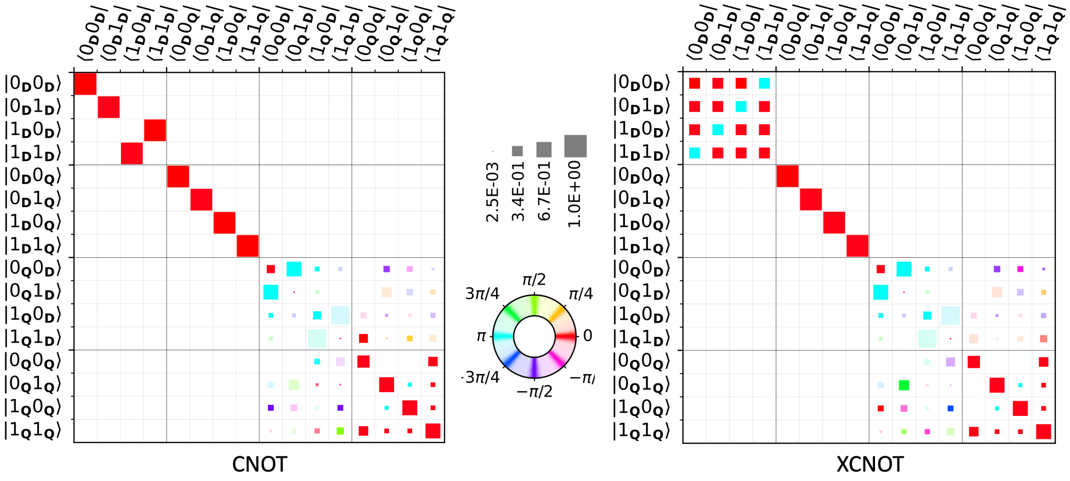

Supplementary Fig. S19. **Matrix representations of CNOT and XCNOT sequences in Fig. S18.** After gauge-averaging we obtain GAQQMap (section Sec. S4 D), which we represent in the Kraus operator-sum representation: $\mathcal{E}(\rho) = \sum_i K_i \rho K_i^\dagger$ acting on a ququart state ρ with Kraus operators K_i. Here we show the average of the Kraus operators $\bar{K}_i$ that summarizes the sequence dynamics. The matrix is organized by the encoded (D) and leaked (Q) property of each of the two DFS qubits. For example, the block "DQ" represents "first qubit encoded, second qubit leaked." The size of each square represents the magnitude of the matrix element, while the color represents the complex phase. The top left of each plot shows the action of the sequence on the encoded state, showing either the familiar structure of CNOT (left) or the less-familiar structure of the XCNOT (right). Both the CNOT and the XCNOT are only directionally leakage-controlled, as evidenced by the large block of non-zero elements in the lower right of both process matrices. This observation, contrasting the leakage-controlled CNOT sequence demonstrated in Ref. [5], highlights the fact that if the first qubit is in the leaked (Q) state, the second qubit is not guaranteed to remain encoded under either sequence.

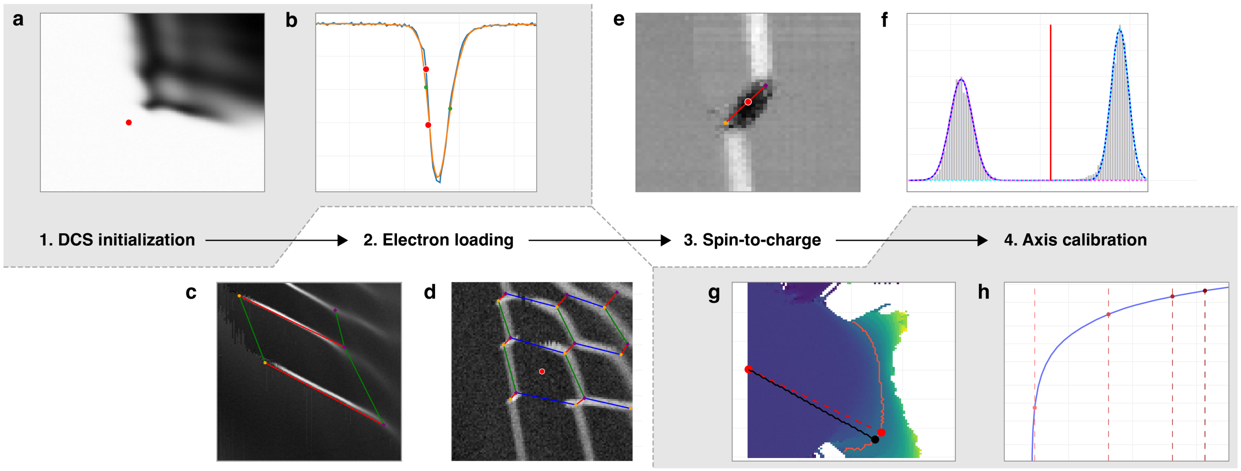

Supplementary Fig. S20. **Tune-up flow for an exchange-only spin qubit which proceeds via dot charge sensor (DCS) initialization, electron loading, spin-to-charge conversion, and coherent axis calibration.** Selected steps of each phase are shown in subfigures. **a,** A Z-gate pinch-off experiment identifies Z-gate voltages where current between the source and drain is pinched off. **b,** A tune-DCS experiment sets M-gate voltage to the point of peak sensitivity. **c,** A plunger-versus-tunnel experiment (PvT) displays electron loading lines as identified by a CNN. **d,** A plunger-versus-plunger experiment (PvP) reveals charge stability cells as identified by a CNN. The idle operating voltage setpoint is automatically updated to the center of the (1,1) charge cell. **e,** A "tiebar" PvP evaluated with a CNN provides a rough measure of interdot tunnel coupling and an initial guess of the spin-to-charge measurement coordinates. **f,** A histogram of measured currents from a spin-to-charge experiment reveals Gaussian peaks associated with the singlet and triplet spin states, a threshold current value, and measurement SNR. **g,** For a fixed exchange pulse duration, the mapping of phase as a function of pulse voltages identifies the symmetric axis and maximizes charge-noise insensitivity [6]. For time-domain control we then choose a single bias point along that axis at the target exchange frequency J. **h,** The exchange pulse duration is swept and a fit provides an invertible pulse duration to exchange angle mapping.

The entangling operations used in this work were derived from the Fong-Wandzura CZ sequence [4]. Adding Hadamards before and after the CZ (Z-controlled-Z), either to the target qubit or to both qubits, gives us a CNOT (Z-controlled-X) or an XCNOT (X-controlled-X), respectively. From here, the sequence can be manipulated in several ways: inserting pairs of π pulses that resolve to identity, commuting Z-rotations with the entire CZ, or permuting pulses allowed by the commutation or Coxeter relations of the exchange algebra [e.g. $E_{ij}(\pi)E_{jk}(\phi)E_{ij}(\pi) = E_{jk}(\pi)E_{ij}(\phi)E_{jk}(\pi)$, for exchange unitary $E_{jk}(\theta)$ executing exchangle θ between spins j and k]. Using these techniques, we manipulate the sequences into a more structured cadence, where pulses on any given spin pair are evenly spaced in time and no spin is ever touched by two consecutive pulses. This cadence roughly doubles the number of pulses required per sequence, but the added structure suppresses error due to pulse-to-pulse crosstalk and imparts partial magnetic noise decoupling. The end result is a set of 45-timestep CNOT and XCNOT implementations (Fig. S18a and b) for all encoded qubit pair layouts on our devices.

Reset-if-leaked (RIL) operations were found using a custom exchange sequence search tool, dubbed SpEQuLator (Spin Exchange Quantum Logic Gate Cooker). SpEQuLator uses a replica-exchange-Monte-Carlo algorithm [7] to search for pulse sequences that approximately implement target unitary operations, then switches to a GRAPE (GRadient Ascent Pulse Engineering) algorithm [8] to refine the sequences to high precision. For RIL specifically, the optimization target is a unitary that (1) acts as an identity when the ancilla is in $|0\rangle$ and the data qubit is unleaked, and (2) deterministically maps a $|0\rangle$ ancilla with a leaked data qubit to a $|1\rangle$ ancilla with the data qubit in some unleaked computational state. The precise state of the data qubit after leakage reduction, or the gate action with an input ancilla in the $|1\rangle$ state, are unspecified. Once we had a SpEQuLator-derived RIL sequence, we applied the same transformation techniques as above to produce a 30-timestep RIL with a regular cadence (Fig. S18c), which we used in the experiments presented here. Other unitary constraints have allowed SpEQuLator to generate operations including CS, iSWAP, CCZ, and leakage-controlled variants of CZ and CNOT.

D. GAQQMap

Considering the notation $|S_{12}, S; m\rangle$ described in the Methods section, recall that encoded qubits live in the manifold $S = 1/2$ and $m = \pm 1/2$. We refer to these four states (two gauge states for each of two qubit states) as D, referring to the gauge "Doublet." However, errors cause leakage into S=3/2 and its Quadruplet of m values, a manifold we call Q. In the presence of arbitrary errors, relative populations and coherences of all eight states could in principle be important, but the lack of spin polarization at small magnetic field renders a symmetry enabling us to neglect the sign of m. We therefore model a gate as acting on states maximally mixed in sign(m), and (in simulation as well as in physical measurement) ignore (trace over) this degree of freedom after each operation. With only this binary degree of freedom traced, our resulting state space for each triple-dot is the ququart of states $|S_{12}, S; |m|\rangle = |0, 1/2; 1/2\rangle, |1, 1/2; 1/2\rangle, |1, 3/2; 1/2\rangle$, and $|1, 3/2; 3/2\rangle$, abbreviated as $|0_D\rangle, |1_D\rangle, |0_Q\rangle$, and $|1_Q\rangle$, respectively. Hence this is a two-dimensional encoded (D) qubit and a two-dimensional leakage (Q) space. This averaged manifold is referred to as a Gauge-Averaged QuQuart (GAQQ), and logical gates, with or without error, can be described by a map between double-Pauli operators on these states, which we call a GAQQMap. We may represent the map in the Kraus operator-sum representation, $\mathcal{E}(\rho) = \sum_i K_i \rho K_i^\dagger$ where K_i are the Kraus operators corresponding to each traced gauge state sign(m). The Kraus operators then intuitively represent the dependence on sign(m). While non-physical, for visualization of our generated sequences, we compute the mean of the Kraus operators of the GAQQMap and organize them by the encoded and leakage spaces of each qubit. See Fig. S19 for examples of this technique applied to CNOT and XCNOT two-qubit gates. For modeling gate error via simulation, which we discuss further in Sec. S6, GAQQMaps including simulated errors from full spin dynamics are created and used to populate event-based simulations.

S5. TUNE-UP AND CALIBRATION

Our Quiver control software includes a large suite of available measurements, analyses, and automated routines that enables users to efficiently tune-up, calibrate, and characterize the qubit device and controller with a high degree of flexibility.

Due to material and fabrication non-uniformity, both within and between devices, device tune-up is exploratory and nondeterministic. Figure S20 shows an overview of the basic tune up workflow divided in to four phases. Effective optimization within and between phases often requires iterative trial, evaluation, and adjustment. Dead ends require returning to known-good checkpoints where alternative paths can subsequently be explored. The software is designed to be flexible for users with loops, backtracking, conditional branching, and adaptive heuristics supported as first-class concepts. In addition to providing all the components required to enable tune-up, we have begun capturing proven workflows that combine the components into automated routines such that tune-up can proceed with increasingly less human interaction.

A. Parallelism

Our software can take multiple routines for tune-up on separate device locations and merge experimental steps to create a routine that executes the multi-location experiment in parallel. Subsequent analysis and automated control-flow logic then occur sequentially. This parallelization technique en-

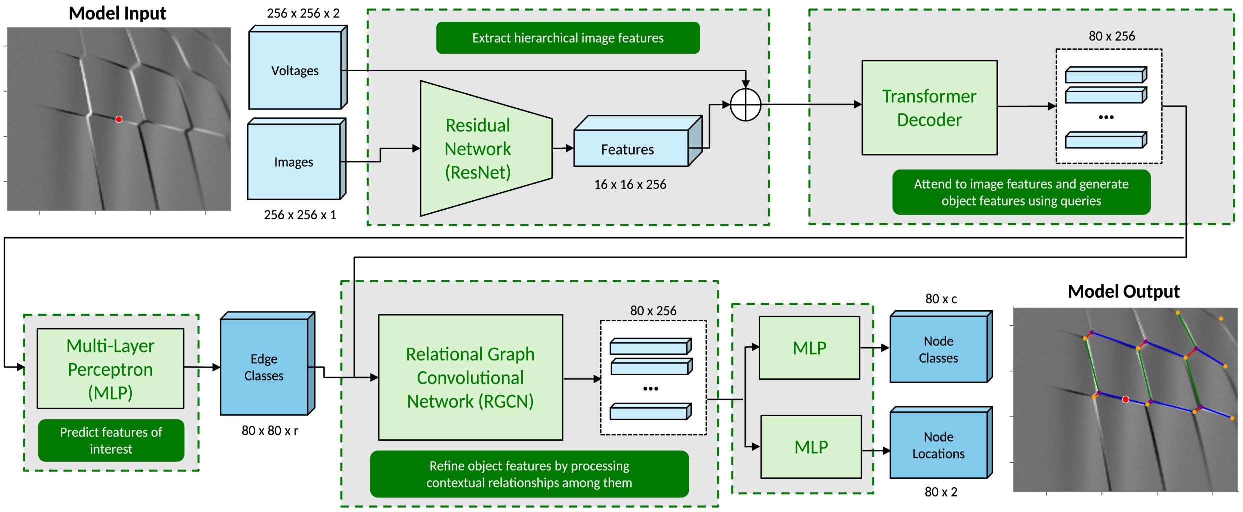

Supplementary Fig. S21. **Baseline CNN model architecture**. A relational network generates high-dimensional embeddings of the information in the image, to which voltage coordinate information is added so the network is aware of voltage space distributions of the features. An attention transformer decoder refines the embeddings which are used by multi-layer perceptrons (MLPs) and a relational graph convolutional network (RGCN) together to predict "keypoint" locations in voltage space as well as the links/relationships between them, which respectively form the nodes and edges of a graph.

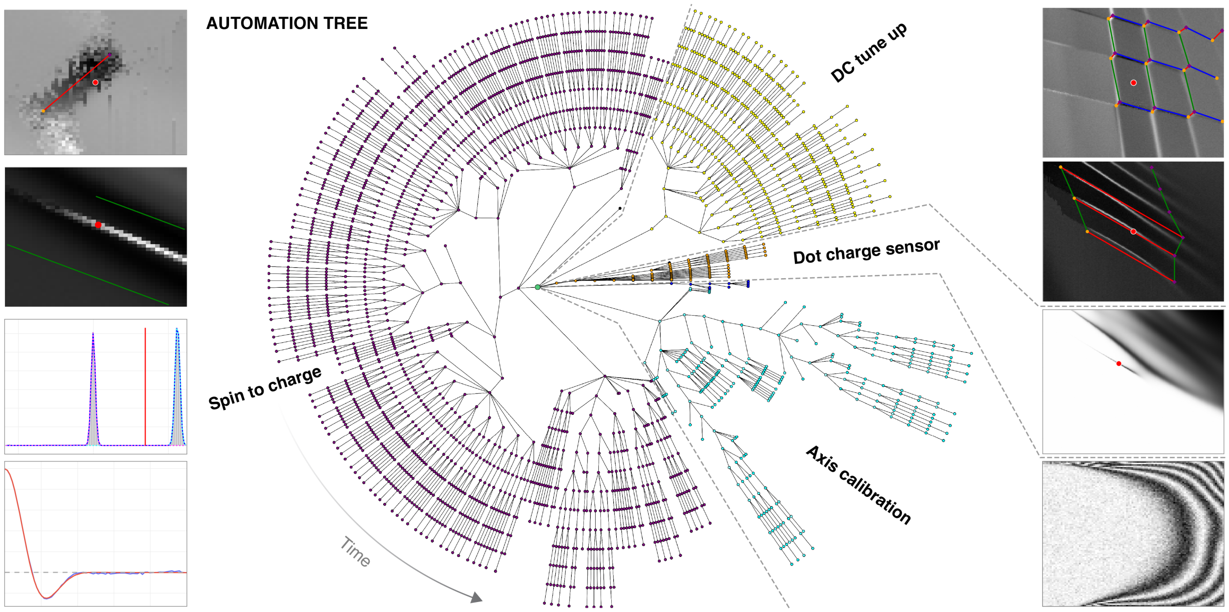

Supplementary Fig. S22. **An example automation tree capturing the sequence of fully automated actions taken during tune up of a single qubit.** The green central node represents the start, and a depth-first traversal indicates the total ordering. The sequence of actions is dynamic, with each action having the ability to insert additional actions (child nodes) or yielding to the parent node. An example of the dynamic nature is when analysis of an experiment (e.g. PvP) indicates unsuccessful determination of the desired operational parameters and further data collection is inserted into the tree.

ables a speedup of up to a factor of 4 to 5 when each DCS is used in the multi-location experiment. We primarily use this technique during the electron loading phase of tune-up, where many charge stability experiments are run at each location to converge on the target location in the high dimensional voltage space. Tune-up and calibration of larger devices is expected to be embarrassingly parallel.

B. Neural nets for charge stability inference

We use custom machine learning models to analyze and extract features from charge-stability data, enabling automated loading of one electron in each plunger gate and the tuning of tunnel couplings [9–17]. We have separate convolutional neural net (CNN) models for PvT, PvP and "tiebar" experiments (tiebar is a PvP experiment zoomed-in on the (1,1)-(2,0) charge transition). Each CNN model is trained on thousands of human-labeled data sets. The CNN models produce a graph of "keypoints" which an automated routine can use to adjust gate voltages to values of physical significance for the state of the device, e.g. electron occupancies or tunneling rates. The baseline model architecture is shown in Fig. S21.

C. Automation

While a simplified tune-up workflow is illustrated in Fig. S21, in practice a tree data structure records the complete sequence of actions performed during a fully automated tune-up run. Each node in this automation tree is an action taken (i.e. data collection, analysis, decision logic, etc.) and a depth-first traversal indicates the total ordering of actions. Each node is automatically evaluated at the time it occurs and given a grade that enables quick assessment of status and later post-hoc interpretability. Such a record is critical after long unattended operation over several hours or days. Furthermore, we leverage trees from multiple runs to perform systematic analysis of success rates and failure modes, enabling iterative improvement to automated routines. Figure S22 is an example of an automation tree for a successful tune-up of a single qubit.

S6. QUBIT CHARACTERIZATION

We use a variety of techniques to characterize the qubit chip and its performance. We highlight some of them here.

A. Initialization window widths

Across nine devices, we estimate two-electron excited-state splittings from the width of the initialization bias and find a median energy splitting of roughly 200 μeV (Fig. S23). This is consistent with Full-Configuration-Interaction (FCI) simulations of orbital-limited excitations in these devices. At

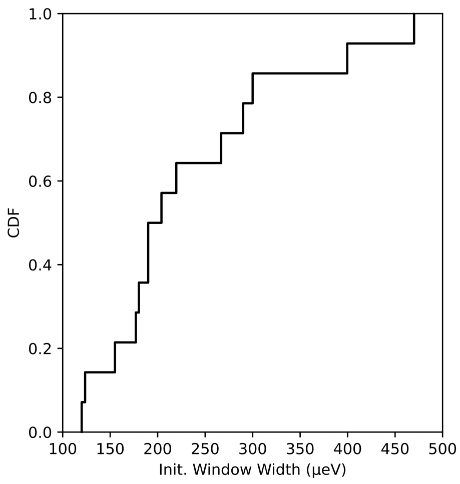

Supplementary Fig. S23. **Measured width of the (2,0)-(3,0) initialization window,** scaled to μeV using a lever arm, sampled from nine different 54-dot devices.

150 mK effective temperature in the device, using this energy splitting in a Boltzmann distribution [3] yields an initialization infidelity of approximately 5×10^{-9}, a negligible contribution to SPAM infidelity and consistent with our observations of it typically being challenging to differentiate from measurement error.

B. Device voltage state

The precise biases applied to each electrode in the configuration employed for the distance-5 repetition code is shown in Fig. S24. For this configuration, all dots that are used as a part of a DFS qubit (e.g. excepting measure dots) are loaded with one electron. This "1^N" occupation is used for all qubits used in this manuscript. All biases applied to each gate electrode fall below 1.0 V, a requirement of the cryo-controller. A few gate electrodes have 0 V applied due to issues in the specific cryo-controller assembly used here that shorted some of the voltages, prohibiting them from being utilized. As noted in Fig 1(b) of the main text, 35 of the necessary static device biases are provided directly from room temperature. These are 24 inner field gates (IFG), 8 bath gates (B), 1 supply gate (SG), 1 outer field gate (OFG), and 1 depleting field gate (DFG). They are not presently sourced from the controller for simplicity, but we anticipate changing that choice in a future system revision.

C. N_{osc} methodology

To evaluate intrinsic charge noise, exchange oscillations are typically measured using room temperature electronics, with or without a cryogenic demultiplexing circuit (referred to here as cryoMUX, or simply MUX). The latter is an in-house, custom cryogenic demultiplexing chip, more similar in concept to

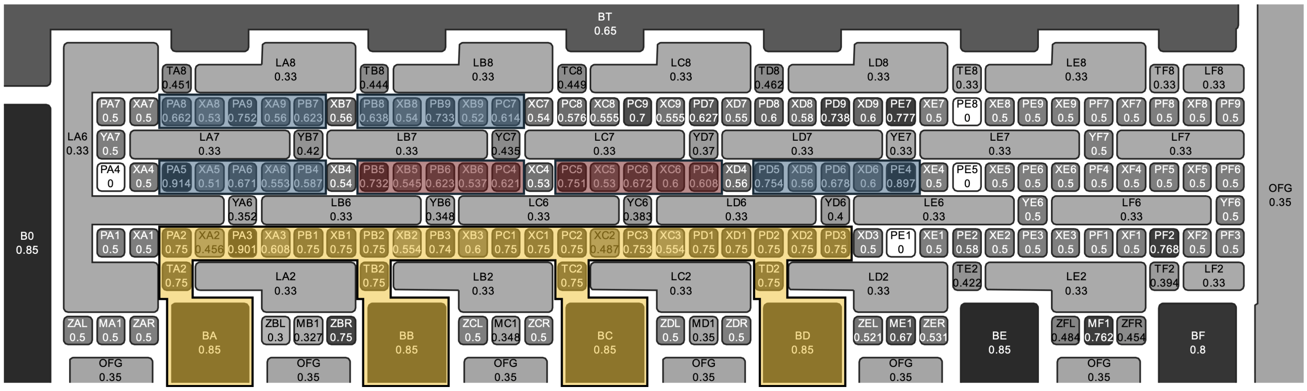

Supplementary Fig. S24. **Biases applied to each gate electrode, in the configuration utilized for the distance-5 repetition code.** Data qubits are shaded blue, ancilla qubits are shaded red, and regions that were "flooded" are shaded yellow (see Sec. S8 A).

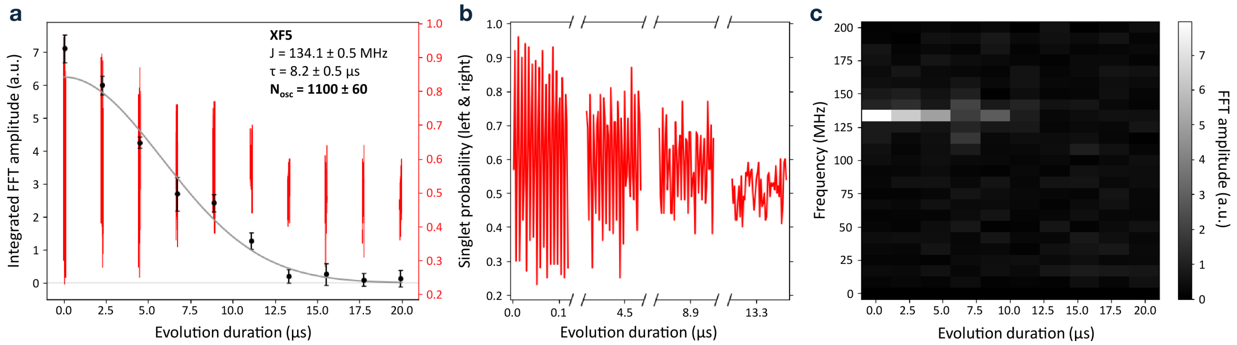

Supplementary Fig. S25. **Stroboscopic N_{osc} methodology.** **a,** Example of stroboscopic exchange oscillation dataset from a sister 54-dot device, showing segmented singlet probability oscillations (red) with corresponding integrated FFT amplitudes (black datapoints) and fitted gaussian decay curve (black line). The extracted decay time of 8.2 µs and measured exchange rate of 134 MHz yield a N_{osc} of 1100. **b,** Expanded view of coherent oscillations in selected segments of the dataset in the left panel, showing stronger coherence at earlier evolution durations. **c,** Grayscale intensity map of the FFT amplitude vs. FFT frequency and evolution duration.

those in [18, 19] than to on-chip [20–24], enabling measurement from a single qubit following control-signals delivered to every qubit in the array. Whether cryoMUX is employed or not, we measure exchange oscillations as a function of evolution duration at a frequency $J/h \approx 100$ MHz, where oscillations can be fully resolved and charge noise is typically the dominant source of decoherence. The observed $1/e$ time τ of the Gaussian decay envelope can be used to calculate the effective number of exchange oscillations $N_{\mathrm{osc}} = J\tau/h$. In the high-$N_{\mathrm{osc}}$ regime, hardware memory constraints can limit the maximum program length uploaded to a room temperature arbitary wave generator (AWG), preventing rapid sampling of the entire decay curve with full time resolution. While portions of the sweep could be compiled and measured sequentially, each portion would sample the local charge noise at different times with inconsistent averaging. When measuring N_{osc} with a cryo-controller we increment evolution duration with sweep pulse repetition count rather than pulse duration, however the stroboscopic sampling technique remains applicable.

We address these limitations by using a stroboscopic time-domain exchange oscillation measurement, sampling the oscillations at full time resolution in multiple equal-length segments along the decay curve. An FFT transform is applied to each segment, where a Gaussian fit is used to extract instantaneous frequency J/h and integrated amplitude. The FFT amplitude of each segment is then fit to a Gaussian decay profile to determine the remaining decoherence parameters τ and N_{osc}.

Figure S25 shows N_{osc} measurement from a 54-dot qubit chip using the stroboscopic methodology. The left panel shows raw data acquired in 10 "chunks." Within each chunk, the results are sampled on a 2.5 ns timescale to fully resolve coherent oscillations. Black data shows the magnitude of the FFT of each chunk, which is a metric for the degree of coherence within that sampling time. A Gaussian fit to the black data yields a decay timescale of 8.2 µs. This quantity multiplied by the exchange frequency 134 MHz, gives a coherence Q-factor N_{osc} of 1100.

The middle panel of Fig. S25 shows sampling of chunks from the first panel, highlighting the coherent oscillations present at early dephasing times, and decohered noisy variation at the latest times sampled. The right panel shows the FFT of each chunk of acquired data, indicating the operational frequency of about 134 MHz and the decay in coherence with

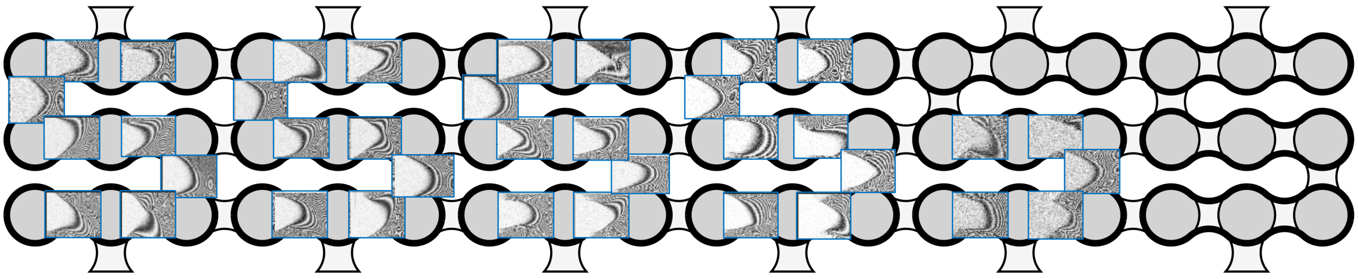

Supplementary Fig. S26. **Representative device fingerprints.** Overlay of exchange axis "fingerprints" characterized (in a MUX system) on a qubit chip of the same generation as that used for the data in the main text. Missing fingerprints in the tested sectors are not because of finite yield, but rather are a detail of how MUX switching works, and were not attempted due to time constraints.

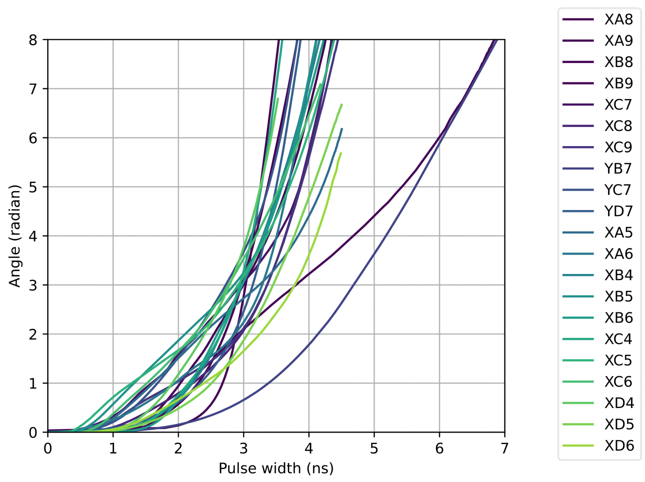

Supplementary Fig. S27. **Rotation-angle-to-pulse-width mapping for all exchange axes utilized in the distance-5 repetition code**. Some axes (e.g. YB7, XA9) suffer from non-idealities in the daughterboard, and consequently targeted a lower exchange rate leading their calibration curves to fall outside the distribution here. Nonetheless, all utilized rotation angles fall within the timing range between 0.3 ns and 7 ns.

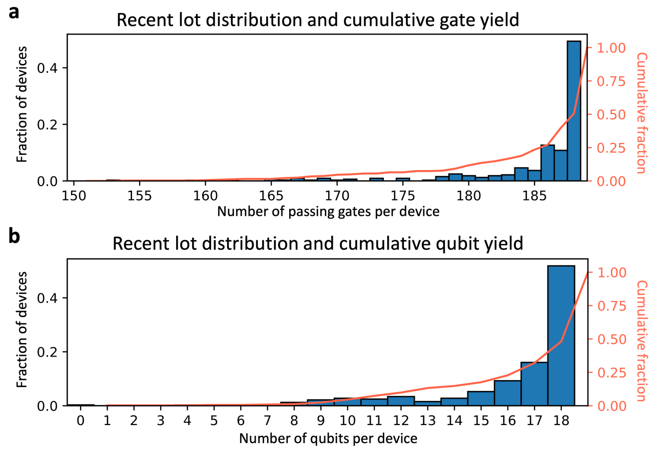

Supplementary Fig. S28. **Wire functional yield from a single device lot, obtained from room temperature wafer probe prior to die singulation.** This representative lot was fabricated more recently than the devices utilized in the main text. **a,** Fraction of devices exhibiting the indicated number of lines passing wafer probing, out of a total possible number of 188 lines (blue, left axis) and corresponding cumulative fraction of devices (orange, right axis). **b,** Fraction of devices exhibiting the listed number of triple-dot qubits with all lines passing wafer probing, as defined from an assumed rectilinear layout of qubits on the device (blue) and corresponding cumulative fraction of devices (orange).

increasing evolution duration.

Note that the N_{osc} data presented in Fig. 3 of the main text was taken for $J/h \approx 100$ MHz. For reasons described in Ref. [1], insensitivity and therefore N_{osc} typically increases with J. Based on the calibration curves in Fig. S27 and accounting for finite pulse rise times, J/h is likely significantly higher than 100 MHz during the QPU demonstrations shown in the main text. Though insensitivity was not characterized in this regime, the reported values of N_{osc} are therefore likely an underestimate.

D. Fingerprint compilation

To illustrate the "quantum functional yield" of the 54-dot devices, we examined the extent to which a "fingerprint" of coherent exchange could be attained on every available exchange axis [6], shown in Fig. S26. This work was pursued in a cryoMUX system with room temperature DACs and AWGs and a cryogenic demultiplexer. This system is limited to individual controllability to nine dots or three qubits worth of channels (each qubit must also be on a distinct row of the device) at a time. This work commenced on the left-most side of the device and progressed to the right, but was halted during tune-up of the penultimate device sector for a non-technical reason. Additionally, the labeled X gate electrodes between sectors that do not have fingerprints superimposed on them are missing here due to the specifics of which device segments were chosen when switching the MUX state, and were not yet attempted at the time this experiment ended. We note that the connectivity of the device layout also allows any individual sub-performing fingerprints to be routed around for coherent operations.

E. Exchange calibration uniformity

The spread of calibration times necessary for all exchange axes utilized in the distance-5 repetition code are shown in Fig. S27. There we plot the mapping of rotation angle (in radians) to calibrate voltage pulse width for each calibrated axis. Two mappings stand out from the more clustered group of calibrations: those for exchange gates YB7 and XA9. Both of these exchange axes utilize gate electrode PB7, whose voltage impulse response suffered from non-idealities of the daughterboard in the system. This subsequently motivated utilizing a lower exchange rate to partially mitigate the effect, reflected in their calibrations. The rest of the curves lie in a relatively tight distribution.

F. Yield of devices

To capture the manufacturability of the 54-dot qubit chips, we present in Fig. S28 the yield statistics of all such devices from a recent fabrication lot. The figure shows the distribution of yield of individual lines on the qubit chip leading up to the gate electrodes (top) and the corresponding number of anticipated operable qubits expected assuming a rectilinear layout of qubits on the device (bottom). This lot exhibits higher yield rates than the lots that sourced the devices reported in the main text, but is typical of recent runs.

G. Valley splitting

Large valley splitting (VS) across an array is a crucial ingredient for most scalable Si spin-qubit technologies. Values of VS can be probed in several ways, most directly through measurements of the single-electron energy spectrum using spectroscopic methods such as detuning axis pulsed spectroscopy (DAPS) [25]. Operationally, signatures of small VS include limited initialization and readout windows [26] and the appearance of dual exchange frequencies in fingerprints due to poor initialization [1]. Previously reported values of VS in Si/SiGe quantum wells range between tens to a few hundred μeV with large dot-to-dot variability (over distances spanning tens of nm to microns), as valley mixing is very sensitive to the local microscopic structure [25, 27–29].

Our devices use an internally-conceived and optimized quantum well design to improve VS and show several operational characteristics indicative of large VS. Fifty-four-dot devices with this design consistently yield well-behaved exchange fingerprints (see Fig. S26) without signs of dual frequencies that often appear in devices with known low VS. Similarly, initialization and measurement operating windows are robust (Sec. S6 A) with large singlet-triplet splittings that range up to several hundred μeV, setting a lower bound for the local VS, and which furthermore change predictably with neighboring gate biases. Those bias trends agree with expectations from FCI simulations which suggest the lowest energy triplet state is typically orbital- rather than valley-limited, since VS is expected to change randomly in response to spatial deformation and displacement caused by changes in bias [30].

In addition to these functional observations of device performance, we use multiple techniques to probe quantum well energy structure, with results which independently indicate a VS range significantly higher than previously demonstrated in Si/SiGe quantum dots. In Fig. S29, we show representative examples of DAPS measurements of the single-electron excitation spectrum. To assign orbital and valley states, we track excited states as we change neighboring gate biases to alter the lateral confinement. While these assignments are not absolute due to valley-orbit mixing, typically we observe differing dependencies in bias for the lowest two excited states. Energies that change significantly with bias are therefore assumed to be orbital-like, whereas the bias-invariant states are assumed to be valley. This is corroborated by the fact that the observed energy changes agree with expectations for orbital states from simulations and previous experiments, as increasing neighboring gate bias reduces the lateral confinement and lowers the orbital excitation energy. While VS can also change due to bias-induced electron displacement, the direction of these changes are again expected to be uncorrelated from dot to dot due to disorder [28, 30]. Using these guidelines, for the instances shown in Fig. S29, we extract valley splittings of 1.04 meV and 1.81 meV. Even if our assignments of orbital vs. valley states are incorrect, the lowest visible excited states are greater than 1 meV, setting a lower bound on the valley splitting. An alternative explanation that VS is too small to be resolved by DAPS (e.g., of order 10 μeV or below, comparable to electron temperature) is inconsistent with the absence of valley signatures in initialization windows and fingerprints.

For further evidence of large VS in our quantum design, we also perform transport measurements of Hall structures in the quantum Hall regime [31, 32]. By measuring the dependence of longitudinal resistivity at low filling factors ν as a function of temperature, the mobility gaps of the Zeeman, valley, and Landau levels can be extracted and identified based on their magnetic field dependences (Fig. S30a). As illustrated in Fig. S30c, we identify the valley splitting of about 2 meV with the summed mobility gap in the form $E_v = \Delta_2 + \Delta_3 + 2\Gamma$. (The higher Zeeman split-level Δ_3 was utilized to extract E_Z in lieu of Δ_1 due to magnetic-field constraints.) The large energy splittings in our structures make it possible to perform these measurements above 1.6 K as both E_Z and E_v are well above the Landau level broadening ($\Gamma \approx 0.36$ meV) at relevant Landau orbital energy values ($E_{orb} \approx 1.5$ meV). VS measured in the quantum Hall regime can differ in important respects from that of few-electron quantum dots, owing to the different electric and magnetic fields involved, averaging over disorder scales, and energy renormalization effects due to electron-electron interactions in an accumulated quantum well [33]. It is nonetheless noteworthy that the magnitude of extracted splittings and the temperature range over which we observe them are significantly higher than previous results in the literature, which find VS between 200-400 μeV at comparable

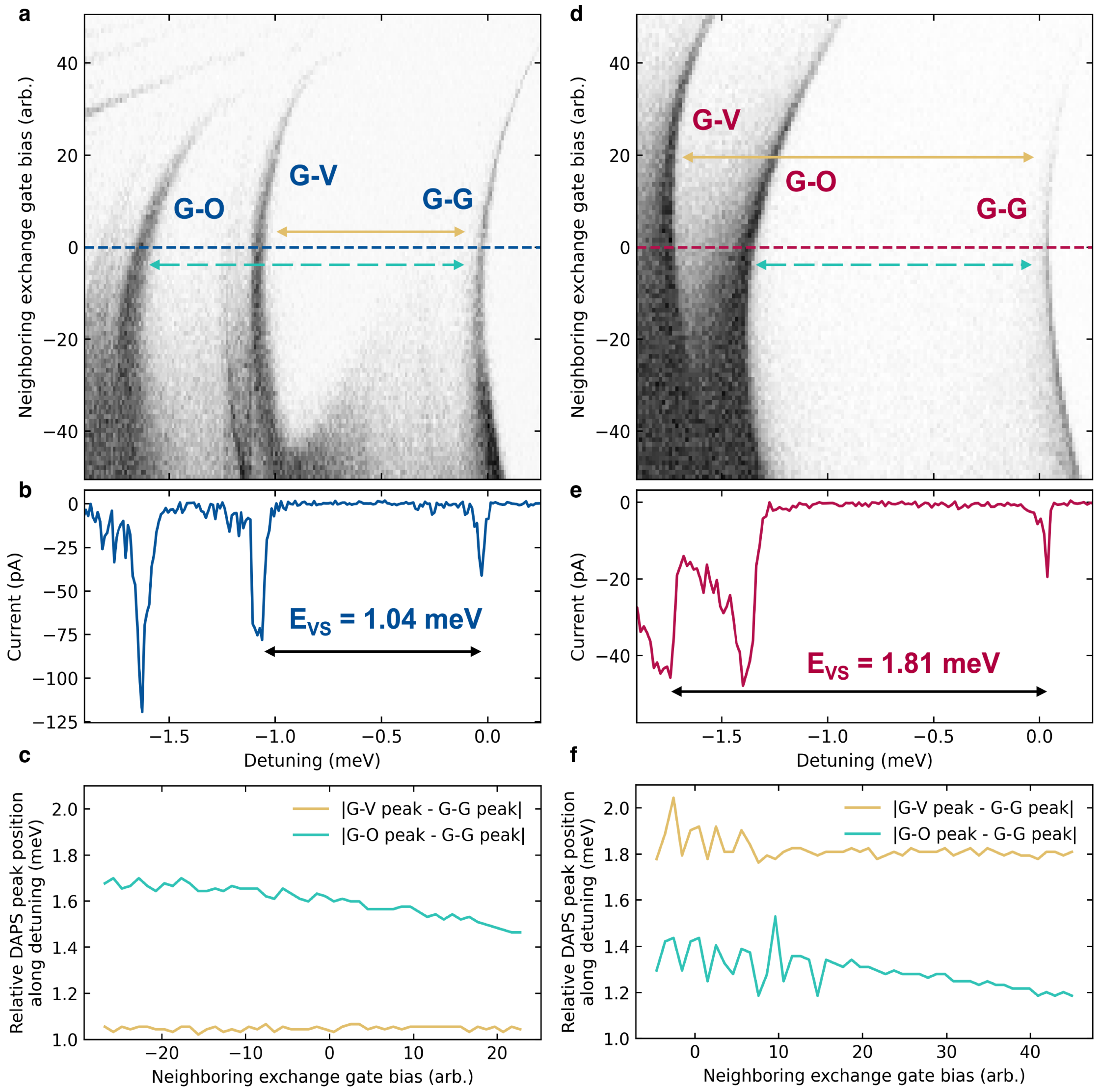

Supplementary Fig. S29. **DAPS measurement of valley splitting and orbital energies**. **a,d**, DAPS differential current measurement vs. detuning energy and neighboring exchange gate bias. Varying the neighboring exchange gate bias changes the orbital confinement. It is therefore expected that the orbital energy will change more strongly than the VS, which allows us to identify each. **b,e**, 1-D line cuts of a,d along the dashed line at zero exchange gate bias. **c,f**, Relative peak position along detuning tracked vs. the y-axis of a,d. Items a-c show an example measurement where the ground orbital excited valley peak (G-V) is easily distinguishable and lower in energy from the first excited orbital ground valley peak (G-O). Items c-f show an example where the valley splitting is larger in energy than the orbital energy, identified as such by DAPS peak response to orbital confinement changes.

fields with visibility below or up to 1 K [31, 32]. Overall, both functional observations and spectroscopic measurements are consistent with the presence of large VS across these devices.

H. Blind randomized benchmarking (BRB)

To characterize the performance of our encoded qubit operations, we relied on blind randomized benchmarking (BRB), as presented in Ref. [34] but generalized to multiple qubits. Like traditional randomized benchmarking (RB), BRB homogenizes many possible error channels into a simple metric of error-per-operation that can be fit to an exponential decay without knowledge of the underlying noise model. The key difference is that BRB does so in a leakage-aware way, so instead of one error metric it produces two: a total error rate ε and a leakage rate Γ.

As with traditional RB, BRB admits an "interleaved" variant (BIRB), where an operation of interest is interleaved into the BRB sequence to characterize its performance specifically. We used BIRB to characterize CNOT, XCNOT, and RIL performance on connected qubit pairs as relevant to the experiment. Figure S31 shows one such experiment, measuring CNOT error on a representative qubit pair. We also show in Fig. S32 an

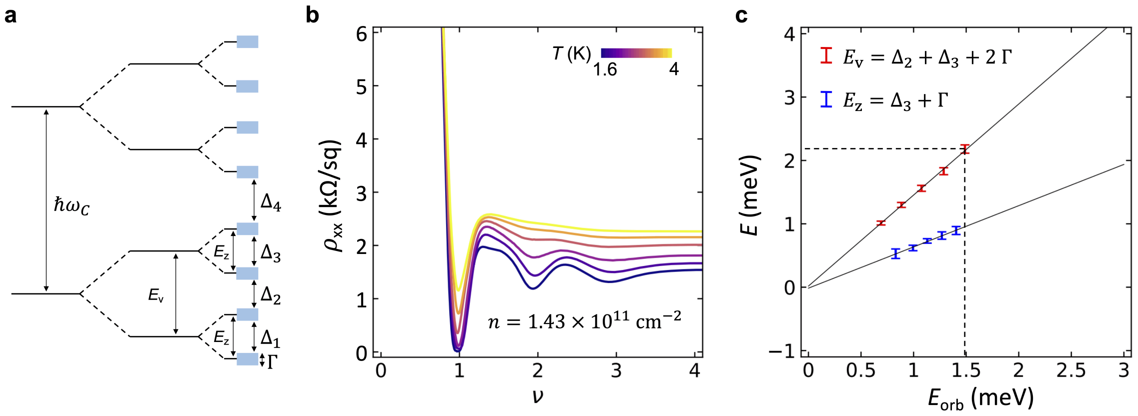

Supplementary Fig. S30. **Valley splitting estimation via the quantum Hall effect**. **a**, Landau level energy ladder in the quantum Hall regime with large valley splitting, including disorder broadening Γ. Landau, valley, and spin levels are split by the cyclotron energy $\hbar\omega_c$, valley splitting E_v, and Zeeman splitting $g\mu_B B$, respectively. **b**, Longitudinal resistivity (ρ_{xx}) as a function of integer filling factor ($\nu = nh/eB$) and temperature (T). **c**, Valley splitting energy ($E_v = \Delta_2 + \Delta_3 + 2\Gamma$) as a function of orbital energy ($E_{orb} = \hbar\omega_c/2 = \hbar eB/2m^*$).

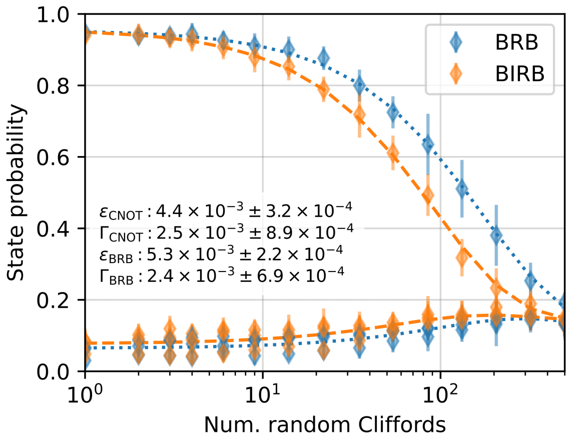

Supplementary Fig. S31. **Interleaved BRB characterizing CNOT error on a pair of qubits used in the [[4,2,2]] experiment.** Dark blue (orange) diamonds show average singlet probability for the two branches of BRB (BIRB), with the vertical lines indicating $\pm\sigma$ from shot and random circuit averaging. Dashed lines show the best fit to equations S5 and S6. Dotted lines show the equivalent fit for non-interleaved "reference" BRB.

example of an exceptionally low-error CNOT, where ε_{CNOT} is so small relative to ε_{BRB} that its uncertainty is greater than its estimated value.

BRB implementation and analysis

The N-qubit BRB experiment proceeds as follows:

1. *Initialize the system in* $|0\rangle^{\otimes N}$
2. *Apply n random 2-qubit Clifford operations,* $C_1, C_2, \ldots C_n$

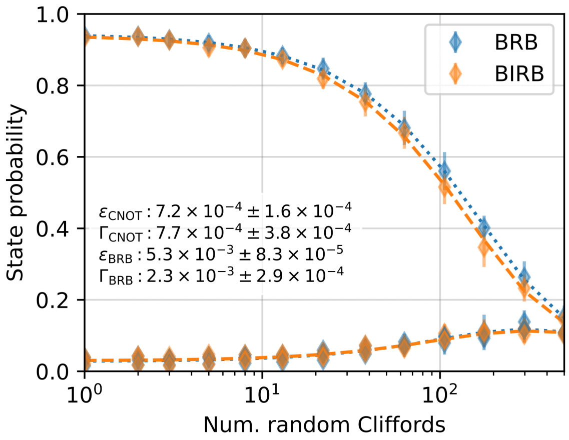

Supplementary Fig. S32. **Lowest-error BRB data to date.** Note that with $\varepsilon_{CNOT} < 0.2\varepsilon_{BRB}$, this CNOT error is not quantitatively reliable; we can also see that interleaved BIRB is failing because $\Gamma_{CNOT} > \varepsilon_{CNOT}$, which is not physical. Still, we can draw two significant conclusions. First, CNOT error is substantially smaller than the BRB error of approximately $5{\times}10^{-3}$. Second, this behavior is consistent with pulse-to-pulse crosstalk being a dominant error source. Because 1Q and 2Q gates have different pulsing cadences, interleaving high-fidelity CNOT operations will increase the pulsing regularity of the sequence as a whole and offset some of the error introduced from the CNOT itself.

3. *Choose a target Clifford C_{tot} from among all combinations of single-qubit identity or Pauli-X operations. E.g. a one-qubit C_{tot} could be either I or X, while a two-qubit C_{tot} could be any of II, IX, XI, or XX.*
4. *Apply an inverting Clifford C_{inv}, chosen such that $C_{inv} \cdot C_n \ldots \cdot C_2 \cdot C_1 = C_{tot}$*
5. *Measure the final singlet population for each qubit*
6. *Repeat across many different choices of random Clifford*

realization C_i, overall Clifford C_{tot}, and Clifford count n.

With no error, $P_{\text{singlet}} = 1$ for the $C_{\text{tot}} = I^{\otimes N}$ branch, and 0 for all other branches. In the limit of large encoded-space error, the system approaches a fully depolarized state, and $P_{\text{singlet}} \to \frac{1}{d}$ for all branches, where $d = 2^N$ is the dimensionality of the qubit space. By contrast, any leaked DFS qubit measures as a triplet, so in the limit of large *leakage* error $P_{\text{singlet}} \to 0$.

To analyze BRB data, we assume that the system undergoes two exponential decays as a function of n: one within the encoded space, and another into a larger, partially leaked space. This is only strictly accurate for a single qubit with a sufficiently strong external magnetic field, but numeric and empirical evidence suggests that it is a good approximation for a wide range of one- and two-qubit error models. Under this approximation and neglecting SPAM errors, the probability P_I of measuring all singlets in the $I^{\otimes N}$ branch is

$$P_I = \frac{1-L}{d} + \frac{d-1}{d}(1-p)^n + \frac{L}{d}(1-q)^n, \tag{S3}$$

where p and q are the encoded and leakage decay rates, respectively, and L is the asymptotic leakage probability. The remaining $d-1$ branches—collectively termed the "X-type" branches—behave very similarly to each other, starting at $P_{\text{singlet}} = 0$ and decaying up to some asymptote $\leq 1/d$. It is convenient to consider the mean singlet probability P_X of all such branches:

$$P_X = \frac{1-L}{d} - \frac{1}{d}(1-p)^n + \frac{L}{d}(1-q)^n, \tag{S4}$$

The sum and difference of these terms can be fit to single-exponential ansatz:

$$P_I - P_X = 2B(1-p)^n, \tag{S5}$$

$$\frac{1}{d}P_I + \frac{d-1}{d}P_X = A + C(1-q)^n. \tag{S6}$$

Finally, we derive error rates accounting for SPAM error following the algebra in [34]:

$$\Gamma = \frac{C}{2B}q, \tag{S7}$$

$$\varepsilon = \frac{d-1}{d}p + \frac{1}{d}\Gamma. \tag{S8}$$

Interleaved BRB

A BRB experiment on N given qubits tells us the average Γ and ε over all possible Cliffords on those qubits. To characterize a specific Clifford of interest U, we modify the BRB experiment to interleave U after each random Clifford, making the full sequence $[C_1, U, C_2, U, \ldots C_n, U, C_{\text{inv}}]$. If errors on U are not strongly correlated with those on the random Cliffords, then the errors will add incoherently in the interleaved BIRB experiment, and we can approximate the errors due to U alone by subtraction:

$$\varepsilon_U \approx \varepsilon_{\text{BIRB}} - \varepsilon_{\text{BRB}}, \tag{S9}$$

and likewise for Γ.

CNOT and XCNOT are Clifford operations with error rates comparable to the random Clifford error rate, so they are usually well-suited to characterization via interleaved BRB (although Fig. S32 shows a striking counterexample). RIL, by contrast, is not a Clifford gate or even an operation confined to the computational space: with general two-qubit inputs (including ancilla), it can produce leaked states. However, if the ancilla qubit is guaranteed to be in singlet $|0\rangle$, RIL acts as an identity on any (unleaked) data qubit state. The process of initializing the ancilla and then applying RIL thus forms a non-unitary, leakage-reducing gadget that can be interleaved into *one*-qubit RB, with random Cliffords applied to the data qubit.

Finally, although SWAP is also a critical component of our experiment sequences, its error rate is much lower than the random Clifford error rate, and its periodic structure can lead to destructive interference with random Clifford error channels. This means that BRB provides unreliable measurements of SWAP errors. While SWAP error is included in our spin-level simulations and therefore in our error budgeting for multi-qubit circuits, we do not rely on experimental interleaved BRB measurements of SWAP error for model calibration.

Random Clifford sampling

To efficiently represent long BRB sequences in limited cryo-controller memory, we rely on the on-chip pseudo-random number generator to sample random Cliffords, as described in Sec. S1 A. For one qubit BRB, we supply the controller with unique sequence definitions for all 24 one-qubit Cliffords and randomly choose among them. For two qubits, we add definitions for SWAP and an entangler (e.g. CNOT), then construct uniformly random two-qubit Cliffords with the following algorithm:

1. *With probability 0.5, apply a SWAP.*

2. *Apply random one-qubit Cliffords to both qubits.*

3. *With probability 0.9, apply the entangler.*

4. *If the entangler was applied in step 3, apply random one-qubit Cliffords to both qubits.*

For some datasets we have applied the entangler with unit probability (rather than 90%), which results in an overly pessimistic estimate for two-qubit Clifford error.

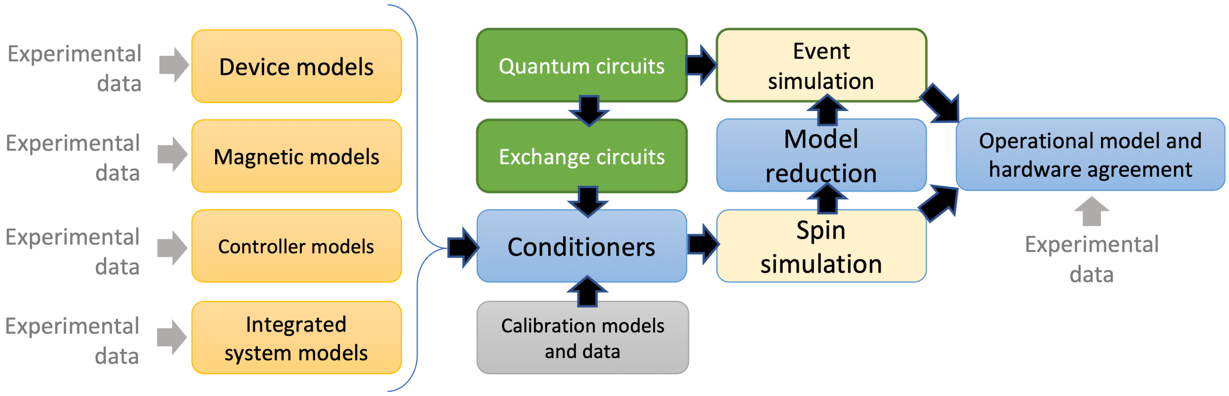

Supplementary Fig. S33. **Schematic summary of models and simulations.** Experimental data feeds semiconductor device models, including charge noise and uniformity; models of noisy magnetic fields including dephasing; models of cryogenic controllers including white and flicker noise; and models of the integrated system including interconnect and impacts on signal fidelity. These models are colored orange. The pulse sequences for experiments originate as quantum circuits, and become compiled into exchange circuits (green). Noise models, as well as models and data from calibration, feed into "conditioners", which apply those models to exchange-pulse sequences. These feed into coherent spin simulations tracking the effects of noisy exchange sequences and magnetic fields on individual spins. Results of spin simulations then populate reduced models, which track Pauli and leakage error rates at the encoded logic gate level. These may be compared with the original quantum circuit in event simulators. Finally, the expected measurement statistics of event simulators, spin simulators, and experimental data are compared to validate the model and indicate need for adjustment at previous steps.

S7. SIMULATION

The combination of qubit characterization data and qubit models with system-level simulations is summarized in Fig. S33. This figure depicts a flow by which data informs models of noisy exchange gates and magnetic fields, which ultimately become simulated via the coherent spin simulation methods to be described in Sec. S7 A. Although charge noise and magnetic noise are important parts of the model, the dominant error in the present demonstration arises from signal integrity issues which require integrated system models to capture; we describe these in Sec. S7 B. These models enable population of errors treated as events at gate-level, including due to contextual miscalibration from signal integrity imperfections; via the process of model reductions, these errors can be included in event simulators as described in Sec. S7 C. The basis for comparing the result of spin and event simulations with experiment is in detector error models (DEMs), methodologies for which we describe in Sec. S7 D. For the present demonstration, operational model and hardware agreement requires an ersatz parameter determination, in which the results of simpler experiments such as randomized benchmarking populate a reduced model for contextual miscalibration, and this parameter is subsequently fed to model multiqubit experiments, a process elaborated in Sec. S7 E. The actual results of such modeling and simulation for the experiments described in the main text are elaborated in Sec. S8.

A. Coherent spin simulation

Our qubit architecture can be modeled as a collection of spins subjected to magnetic fields and interacting with each other via the exchange interaction. This is described by the following Hamiltonian:

$$H(t) = \sum_j \gamma_j \mathbf{B}_j(t)\cdot\mathbf{S}_j + \sum_{\langle j,k\rangle} J_{jk}(t)\mathbf{S}_j\cdot\mathbf{S}_k. \qquad \text{(S10)}$$

Here, γ_j is the gyromagnetic ratio for the electron in dot j, $\mathbf{S}_j = \sigma_j/2$ the spin operator for the electron in dot j, and $\langle j,k\rangle$ denotes nearest neighbors. The magnetic fields $\mathbf{B}_j(t)$ for each dot are a sum of the global applied magnetic field, any static gradients present in the device, along with fluctuating effective fields of magnitudes consistent with those originating from hyperfine interactions with stray spinful nuclei [35]. The time-dependent exchange couplings $J_{jk}(t)$ encode both the applied pulses (which we approximate as square pulses), as well as fluctuations due to charge noise.

We solve the time-dependent Schrödinger equation under the Hamiltonian in Eq. S10 using a custom software package called the python Quantum Exchange Simulator Tool-suite (pyQUEST). Simulations of our qubit architecture are run in pyQUEST following these steps:

1. The user passes a *resource topology*, which provides the device graph, whose nodes are the spins and whose edges are the possible exchange couplings. The topology also may or may not contain an *encoding*, which describes how spins are grouped to form DFS qubits. We use the encoding for GAQQMap (Sec. S4 D) to track spin labels belonging to DFS, but in general circuit simulation we only rely on the collection of spin and their connectivity.

2. The user passes a *flatseq* object, which is a data structure that contains the sequence of pulse operations and

state preparation and measurement (SPAM), operations to apply to the spins.

a. This is typically encoded as a matrix, where each row is a timestep, each column a particular exchange axis, and each entry either zero or the desired pulse angle.

b. A non-zero pulse exchangle θ for an axis between i and j gives the time-integral of the exchange coupling $J_{ij}(t)$ over the duration of the pulse. Explicitly, we have:

$$\theta = \int_{t_1}^{t_2} J_{ij}\left(t'\right) dt'.$$

The start and end times t_2 and t_1 for each time step are inferred from user-supplied data.

c. State preparation (i.e., flushing to a singlet) and measurement are indicated by an additional list of flags specifying at which timesteps the operation(s) occur, and on which exchange axis.

3. The user specifies noise parameters for the system, such as N_{osc} for each axis or T_2^* for each dot, the (static) magnetic field experienced by each spin, and static pulse miscalibration. Note that in pyQUEST, T_2^* is a property of each *dot*, rather than each *exchange axis*. For a DFS qubit with three spins $\{z, t, n\}$, with pairwise T_2^* values $\left\{T_2^*(z,t), T_2^*\left(z,n\right), T_2^*\left(t,n\right)\right\}$, the effective T_2^* inputted to simulation for all three spins is

$$T_2^* = \left[\frac{1}{3}\left(\frac{1}{T_2^*\left(z,t\right)^2} + \frac{1}{T_2^*\left(z,n\right)^2} + \frac{1}{T_2^*\left(t,n\right)^2}\right)\right]^{-1/2}.$$

4. Pyquest then generates a *noise trajectory* (i.e., a particular sampling of fluctuating magnetic fields or fluctuating exchange couplings) and simulates application of the sequence with the sampled noise trajectory. This process can be repeated for a desired number of shots, with pyQUEST automatically re-sampling noise for each shot.

Fluctuating magnetic fields at each dot, along with fluctuating exchange couplings, are generated as $1/f$ noise via the Voss algorithm [36]. When dealing with time-dependent couplings $\mathbf{B}_j$ or J_{jk}, we trotterize the applied operators; e.g., for a single-spin magnetic field term we do:

$$\mathcal{T}\left\{\exp\left(-\frac{i\gamma_j}{\hbar}\int_{t_1}^{t_2}\mathbf{B}_j\left(t'\right)\cdot\mathbf{S}_j\,\mathrm{d}t'\right)\right\} \mapsto \prod_{n=0}^{N}\exp\left[-\frac{i\gamma_j}{\hbar}\mathbf{B}_j\left(t_1 + n\frac{t_2-t_1}{N}\right)\cdot\mathbf{S}_j\frac{t_2-t_1}{N}\right], \tag{S11}$$

where $\mathcal{T}$ is the time-ordering operator, $N = \lceil\left(t_2-t_1\right)/\tau\rceil$, and τ is the splitting time. Exchange, i.e. N_{osc}, noise is trotterized similarly. For the simulations presented in this manuscript, we take the splitting time to be $\tau = T_2^*/20$, where T_2^* is the *shortest* T_2^* across all spins involved in the operator being trotterized.

Pyquest has two different backends for performing simulations: one using dense state vector methods, and the other using tensor network methods (more specifically, matrix product state methods). We provide high-level overviews of these simulation backends below.

Spin state vector (SSV)

The spin state vector (SSV) backend stores the state of the N-spin system in a dense state vector with 2^N entries. State vectors in the computational basis are mapped on to indices in the state vector in the standard way: namely, the state $|\sigma_0\sigma_1\ldots\sigma_{N-1}\rangle$, with $\sigma_i \in \{0,1\}$, corresponds to index $\sum_{j=0}^{N-1}\sigma_{N-1-j}2^j$ in the state vector (see, e.g., Ref. [37] for details).

Single-spin magnetic field terms on dot j, $\mathbf{B}_j\cdot\mathbf{S}_j$, and exchange terms $\mathbf{S}_j\cdot\mathbf{S}_k$, are constructed respectively as 2×2 and 4×4 matrices in Pauli basis. Single-spin terms are exponentiated directly for stepwise integrated fields after trotterizing as described above. Matrices for multi-spin terms (e.g. exchange with fields or multiple overlapping exchanges) are either directly diagonalized and exponentiated (if involving three spins or fewer) or trotterized to consecutive single or two-site terms (if involving more than three spins). These operators are then applied to the state vector by iterating over all possible configurations of the spins not involved in the operation.

To implement a SPAM operation on a given spin pair, we first construct the projectors onto the four possible angular momentum states $\{S, T_0, T_+, T_-\}$ for the pair in question. In the computational basis, these read:

$$P_S = \frac{1}{2}\begin{pmatrix}0&0&0&0\\0&1&-1&0\\0&-1&1&0\\0&0&0&0\end{pmatrix}, \quad P_{T_+} = \begin{pmatrix}1&0&0&0\\0&0&0&0\\0&0&0&0\\0&0&0&0\end{pmatrix},$$
$$P_{T_0} = \frac{1}{2}\begin{pmatrix}0&0&0&0\\0&1&1&0\\0&1&1&0\\0&0&0&0\end{pmatrix}, \quad P_{T_-} = \begin{pmatrix}0&0&0&0\\0&0&0&0\\0&0&0&0\\0&0&0&1\end{pmatrix}. \tag{S12}$$

Next, the probabilities $p_i = \langle\psi|P_i|\psi\rangle$, for $i \in \{S, T_0, T_+, T_-\}$, of each angular momentum state are computed. Then one outcome is sampled (weighted by the probabilities p_i) and the appropriate projection operator is applied. For measurement, we apply either P_S (if the sampled result is S) or $P_{T_0}+P_{T_+}+P_{T_-}$ (if the result is one of the triplet states) to the state and renormalize. In either case, the measurement result is recorded. For initialization, we project to the same state that was sampled, and then apply a correction:

- For T_0, we apply a rotation by π about the z axis, $\exp\left(-i\pi\sigma_z/2\right)$, to the first spin.

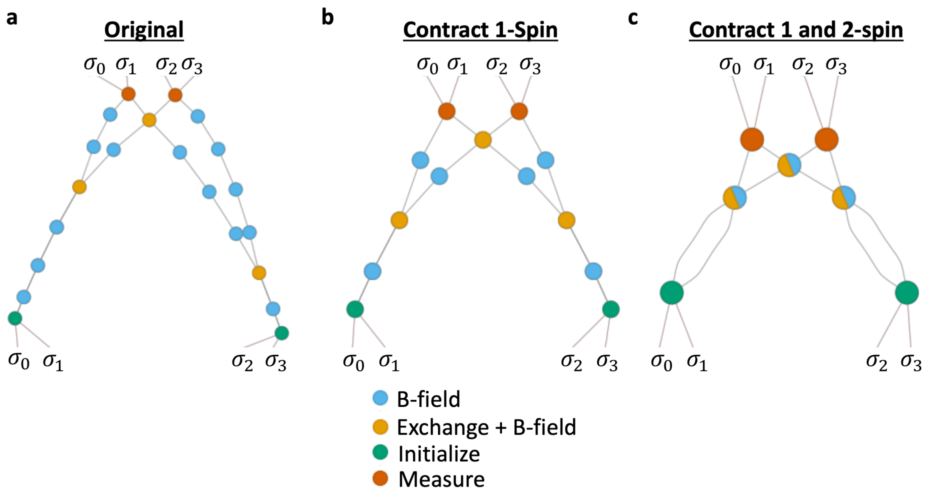

Supplementary Fig. S34. **Example of contracting the tensor network representing evolution under a given $\mathbf{H}(\mathbf{t})$.** In this case, we have a four-spin system, where two pairs of spins are initialized into singlet pairs, then exchange operations are performed on three pairs of spins, and then singlet/triplet measurements are performed. Between all these operations, the system idles in the presence of a magnetic field. **a,** Initial tensor network, with every single and two-spin operator represented by a single node. **b,** Tensor network after contracting all single-spin (i.e., magnetic field evolution) terms together. **c,** Tensor network after contracting all single-spin operators into nearby two-spin operators. Note the dramatic reduction in the number of nodes in the network; this contraction scheme represents a significant savings in memory compared to the initial network. At this point, one could also contract two-spin operators together as well, to reduce the number of SVDs required; this could be potentially useful for networks that have a deep network of nodes acting an isolated subset of the spins (as is the case, e.g., when performing a multi-qubit gate on a subset of spins in a large system).

- For T_+ and T_-, we apply a rotation by π about the x axis, $\exp\left(-i\pi\sigma_x/2\right)$, to the first spin, and then apply the operator P_S and re-normalize.

In either case, the result of these corrections is that the system is in the singlet (S) state; the purpose of doing initialization in this manner is to correctly simulate the back-action of the initialization on the rest of the system. For initialization, we can also simulate errors via an affine transformation of the probabilities

$$\begin{pmatrix} p_S \\ p_{T_0} \\ p_{T_+} \\ p_{T_-} \end{pmatrix} \mapsto A \begin{pmatrix} p_S \\ p_{T_0} \\ p_{T_+} \\ p_{T_-} \end{pmatrix} + |B\rangle\,, \tag{S13}$$

with A a 4×4 matrix and B a 4-element vector parameterizing the initialization error. The outputted state can then be sampled from these new probabilities, and constructed by reversing the corrections performed above. Measurement errors can be simulated after the fact by probabilistically altering the recorded measurement results.

In SSV, when there are no overlapping exchange operators applied at the same time, all the operators applied to the state are constant size (2×2 or 4×4). Applying gates or SPAM operations to the state is then $O\left(2^N\right)$ in both space and time, rather than $O\left(4^N\right)$ when constructing the full matrices. The looping over configurations of uninvolved spins can also be done very efficiently using bit-twiddling techniques and pointer arithmetic. Consequently, the SSV backend has very good performance for small ($\lesssim 7$ DFS qubits, i.e. < 21 spins) systems. This is particularly advantageous for simulating one and two qubit RB.

Matrix product state (MPS)

For larger simulations than SSV can easily handle, a matrix product states (MPS) backend is used. The MPS backend stores the state of the N-spin system as an MPS with a maximal bond dimension $\chi_{\max}$. Each spin can be assigned to a single MPS site, or multiple spins can be grouped onto a single MPS site. Operators for single and multi-spin terms are constructed analogously as in SSV and applied to the *physical* indices of the MPS. Singular value decompositions (SVDs) are then performed as needed, along with truncation of the bond dimension. SPAM operations are implemented analogously as in SSV.

We use a modified version of the open-source quimb package [38] which implements methods for tensor network operations (including contractions). Importantly, this package represents the evolution under $H(t)$ for the whole experiment as a tensor network, which allows the software to efficiently contract certain types of operations [39]. For example, consecutive operations occurring on the same MPS site (e.g., consecutive single-spin terms on the same spin, or exchanges acting on a pair of spins that are grouped in the same MPS site), and even operations occurring on neighboring MPS sites, can be contracted into a single operator. See Fig. S34 for an example. This latter case is important, as fusing together multiple operations between the same pair(s) of MPS sites can reduce

the number of SVDs required and improve performance.

Our MPS simulation backend can also be run on one or more GPUs via a user-passed argument. This is achieved by using the autoray [40] library to dispatch tensor operations to the appropriate backend. To run tensor operations on GPUs, we use cupy [41] as the backend. For large systems and/or deep networks, this allows for significant improvement in simulation wall time; indeed, the simulations of larger circuits ([5,1,5] and [[4,2,2]]) presented in this manuscript make use of this feature. The network contraction described above is a vital feature when running the MPS backend on GPUs, as it ensures the network does not exceed the amount of memory available to the GPU.

Due to the overhead involved with storing the state as an MPS and contracting the tensor network, MPS is generally slower than SSV for systems with $\lesssim$ 7 DFS qubits. Above this scale, the memory savings from truncating the bond dimension ($\chi_{\max}$) leads to better performance relative to SSV (especially when run on a GPU). Our MPS simulator tracks the norms of the MPS after SVD truncation, allowing us to assess the reliability of the MPS approximation at a given $\chi_{\max}$. A crucial consideration when setting up an MPS simulation is how to group the spins into MPS sites: putting more spins onto each MPS site can reduce the number of required SVDs, at the cost of larger MPS matrices (for the same max bond dimension). We find empirically that grouping the three spins of each DFS qubit into their own MPS site provides near-optimal performance, and so use this grouping for all MPS simulations presented here.

B. Interconnect simulation

In the previous section, we explained how the dot-level magnetic and charge noise parameters extracted from our tune-up and calibration experiments are added to a device simulation. However, there are also control errors that can occur in an experiment which are not caused by local noise near the qubits in the device. To that end, we have developed a framework to more accurately model how individual sources of control error propagate through our quantum simulation framework via "conditioners". In general, "conditioners" are software objects that consume a sequence of ideal exchange angles and return a modified sequence whose pulses have been altered in some way. For this specific use-case, we have designed a conditioner that captures how pulses generated by the controller propagate through the entire signal chain to the dot(s), which is implemented via linear & time-invariant (LTI) techniques in conjunction with direct time-domain simulations. Using this conditioner framework, we capture both voltage-domain pulse errors from realistically-generated pulse shapes and unintended overlap of subsequent pulses (inter-symbol interference or ISI) and cross-talk (XTK) between pulses on separate control lines.

Interconnect circuit model

We model voltage propagation through our entire analog signal chain assembly, consisting of a cryo-controller, various interconnect sub-assemblies and the qubit chip. Voltage pulses are simulated in the time-domain using industry-standard Simulation-Program-with-Integrated-Circuit-Emphasis (SPICE) simulation software. ISI and XTK, driven by component design as well as unavoidable impedance mismatches, result in complex and unpredictable pulse reflections whose errors can only be fully-accounted via SPICE simulation of the full-scale, end-to-end interconnect of the integrated system. Such a circuit model is constructed by chaining together a series of (sub)circuit multiconductor models corresponding to individual components of the assembly (Fig. S35). These components span several orders of magnitude in physical size and embody a range of electrical complexity that merits the application of a unique modeling approach for each element in the end-to-end model. We have developed methods for generating high-fidelity models based on cross-sectional 2D TEM analysis, 3D full-wave electromagnetic simulations as well as lumped element models where appropriate. The end-to-end circuit model is used to generate the multi-input multi-output (MIMO) impulse response by performing a series of transient SPICE simulations, which is then convolved into sampled controller waveforms and mapped into the singlet-triplet exchange domain, to add the expected angle rotation errors to the ideal exchange sequence.

V-J device model

Unwanted cross-capacitive coupling between metal gates and electrons is a significant source of control error. This type of "front-end-of-line" (FEOL) error is distinct from the "back-end-of-line" sources discussed in the previous section. It also will not be observed during single-axis procedures, and therefore is not accounted for in a local noise term like N_{osc}. To account for FEOL crosstalk, we require a voltage-to-exchange mapping that turns the applied voltage V on each gate into an effective exchange coupling J on each pair of dot-confined electrons. We generate this V-J map by combining a spatial lever-arm function and individual axis fingerprints from a single simulated device tune-up. Time-domain voltage signals obtained via impulse response convolution are projected into a transformed basis ("detuning," "anti-detuning," and "symmetric" axes) which more accurately captures the operational modes of an axis between two dot-confined spins. Then, time-domain exchange energies per axis are estimated using rectangular spline interpolation of transformed-basis points on a log-normalized J map. An example of this procedure is shown in Fig. S36a. In summary, this procedure converts a complicated, time-dependent voltage trajectory on multiple gates into a single number which predicts the total exchange applied to two dot-confined spins.

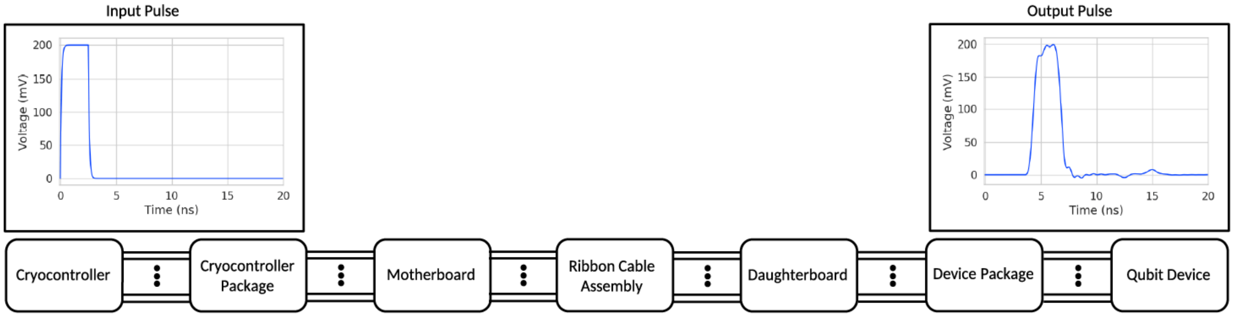

Supplementary Fig. S35. **A high-level block diagram of the end-to-end SPICE circuit model of the system interconnect.** Each block in the signal chain represents a discrete SPICE component model that has been developed to model the specific electrical characteristics of that component. These component models are obtained by applying a variety computational electromagnetic techniques and lumped-element equivalent models. A custom-developed python interface allows the users to easily specific the nets of interest, generate SPICE netlists for each component, and chain the components together to simulate the end-to-end integrated system.

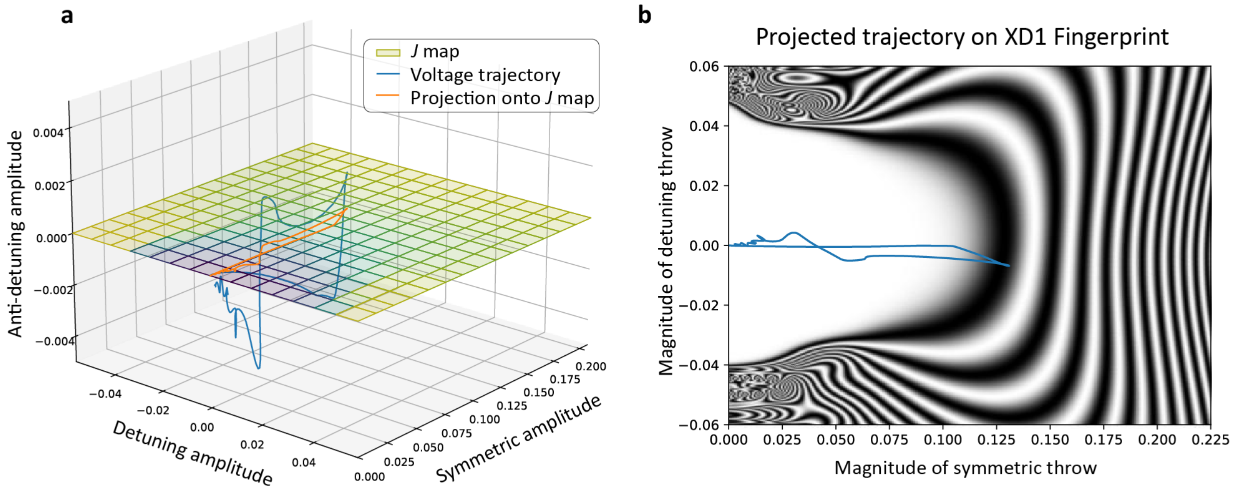

Supplementary Fig. S36. **Pulse trajectory simulation. a,** Projection of 3-dimensional voltage trajectory of a 2.5 ns pulse onto a transformed-basis plane. **b,** Detuning and symmetric axes are defined by the detuning and exchange throws of the simulated fingerprint, respectively.

Gate error predictions for ISI and crosstalk

Using the interconnect circuit model and the V-J model, we can now make predictions of how the electrostatics acting on the control lines and FEOL affect qubit performance. Figure S37 compares a simulation of a two-qubit BIRB sequence with only microscopic noise (“noise”), one with only control error (“miscal”), and one with both (“miscal+noise”). In Fig. S38 we also compare a noise-free (“ideal”) gate to one with control errors (“miscal”) along specific Pauli-error channels, as computed by GAQQmap (see Sec. S4 D). By looking at the differences between each model’s total gate error, we can isolate the contribution of ISI and XTK to CNOT infidelity (see Sec. S6 H). It is informative to study how the relative amount of control error changes as one modifies the pulse cadence, or “pulse-to-pulse time” t_{pp}. We find that at a fast cadence ($t_{\mathrm{pp}} = 8$ ns), the control error is almost five times larger than the microscopic noise sources. However, at the cadence used in our multiqubit demonstrations ($t_{\mathrm{pp}} = 20$ ns), the control error is almost ten times *smaller* than the microscopic noise source.

The strong dependence of gate error on pulse cadence is not surprising. At short t_{pp} (< 16 ns), Same-Wire Intersymbol Interference (SWISI) from pulse tails is large, and pulses that are nearby spatially and/or temporally will experience a large integrated angle error. As we move toward the standard cadence of $t_{\mathrm{pp}} = 20$ ns, differences in the estimated CNOT error with and without miscal become much smaller, within the uncertainty of Clifford and noise sampling in the simulation. This indicates that SWISI and electronic crosstalk are not large contributors to the total error seen in experiments with typical operating parameters (calibration, pulse cadence, voltage operating points, etc.) and with well-behaved sequence structure (no same-axis consecutive pulses, etc.).

It is important to note that these simulations do not include internal cryo-controller non-idealities, and the device model

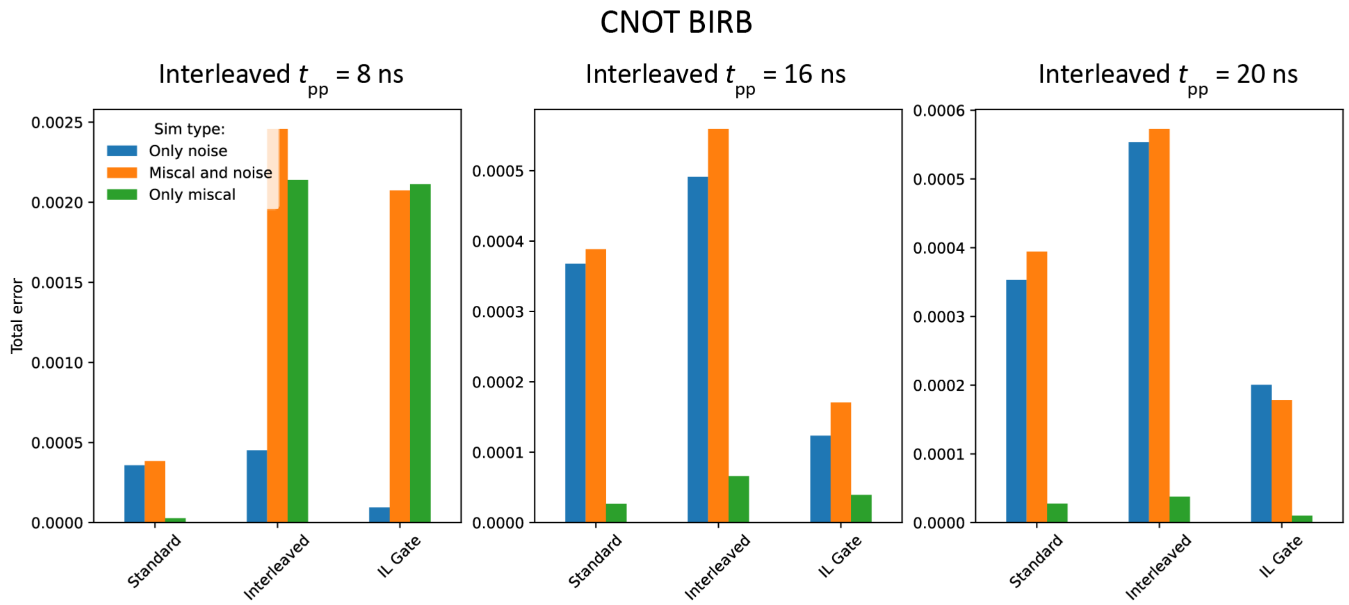

Supplementary Fig. S37. **Coherent spin-simulations of a two-qubit BIRB sequence with an interleaved CNOT, varying the amount of time between the rise of adjacent pulses (pulse-to-pulse time, or t_{pp}).** The three groups of bars represent errors from standard RB error (left), total interleaved RB error (middle), and the inferred interleaved gate error (right). Bar colors represent simulations under an ideal sequence without noise (blue), a sequence with both control error and microscopic noise (orange), and a sequence with control error but without microscopic noise (green). The random Clifford gates used in the BIRB sequence have t_{pp} = 20 ns, and single-axis exchanges were calibrated with t_{pp} = 24 ns.

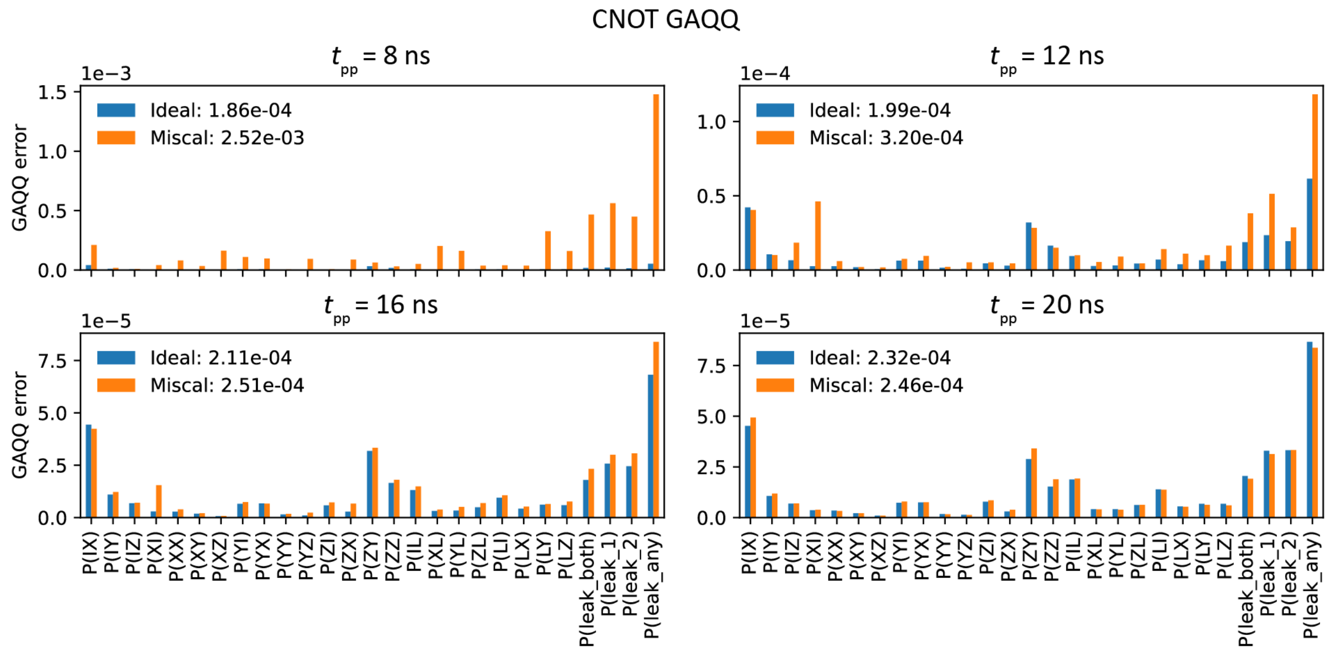

Supplementary Fig. S38. **Error channels, as computed by GAQQMap (Sec. S4 D) of the CNOT gate errors, with and without control error, at varying pulse cadences t_{pp}.** Single-axis exchanges were calibrated with t_{pp} = 24 ns, and we used microscopic noise parameters of N_{osc} = 100, T_2^* = 50 µs.

does not account for differences between real device tune ups and those generated by our simulation. In a following section (Sec. S7 E), we use this finding to argue that cryo-controller errors are the likely source for a majority of the observed gate and circuit infidelity.

C. Event simulation

Coherent simulation of each electron captures many microscopic noise phenomena, but the computational cost of the simulation grows exponentially with the number of spins. For this reason, we turn to qubit-level event simulation, which is a common approach for evaluating quantum circuits in the presence of noise-induced errors. All sources of microscopic noise are combined into an effective probability of a single encoded qubit error (explained in the GAQQmap procedure in Sec. S4 C), and the quantum device is simulated as a series of expected (logical) and unexpected (noise) gates.

Our internal software package which handles event simulation of circuits is called Squeaky. Squeaky simulates quantum programs acting on DFS qubits made of three spins, and relies on an abstract syntax tree (AST) data structure. To more reliably simulate our exchange-only qubit architecture, leakage out of the encoded subspace is tracked by a leakage bit assigned to each qubit. When a leakage error occurs, this leakage bit is flipped to true, and the leakage is then propagated through the circuit according to the leakage properties of the applied gates. Leakage is only removed in this simulator when a qubit is reset to the encoded $|0\rangle$ state.

Noise is injected into an ideal circuit via the circuit's AST representation, which enables efficient calculation of when potential faults can occur and what their error distributions are. After this analysis, potential faults are inserted into the tree. Figure S39 provides an example of a simple program before and after noise injection. After this injection of potential faults, one then samples the circuit many times (usually in parallel, with use of MPI), stochastically replacing potential faults with realized faults before each shot.

We have used two different methods to evaluate the noisy circuits described above. The first is a coherent-qubit evaluator, where each qubit is represented by a single spin-1/2 degree of freedom (used for the [3,1,3] simulations). Note that this is distinct from the coherent-spin simulator of Sec. S7 A, where each qubit is represented by *three* spin-1/2 electrons. The other evaluator is a Pauli frame tracker, with an additional bit attached to each qubit to indicate whether it has leaked or not (used for the [[4,2,2]] simulations).

D. Detector error models (DEMs)

Detector error models (DEM) can be estimated from experimental and simulated data to better understand both the qubit systems and simulators. A DEM is a list of detector error events E_i and their associated probabilities p_i for occurring. The E_i can be represented as length-N bitstrings that add together modulo 2. A sample x can be generated from a DEM of L events of size N via the equation $x = E \cdot q$ where E is the $N \times L$ matrix whose ith column is E_i and q is an L-bit binary vector generated from the probabilities whose ith entry is 1 with probability p_i and 0 otherwise.

A benefit of DEMs is that they allow for insights into higher weight events that objects like p_{ij} matrices are ill-equipped to provide. Recent progress summarized by Young and Blume-Kohout [42] describe how to estimate a DEM that fits the detector data from many shots of a quantum error correcting code. Their codebase is available in pyGSTi [43], and we have used that to perform our DEM analysis. However, the cost of estimating DEMs scales exponentially with the number of detectors N and so is computationally intensive for $N > 16$. This limits us to estimating DEMs for small and short codes. Nonetheless, this still provides a way to probe high weight error events for such cases and diagnose the ways in which our simulations agree and disagree with experiment.

Our workflow for doing this DEM comparison is as follows. First, we run the experiment or simulation and save the detector data to a universal format. Then, we use pyGSTi to estimate a DEM from each dataset. Finally, we compare between experiment and simulation results in two ways. First, we compare probabilities event-by-event to probe how specific errors behave. Second, we coarse-grain an event into a weight class and compare the total probability for each weight class of error events. This second approach allows us to evaluate whether the probability of high-weight events are similar between two datasets even when the probability of each individual event is small compared to the total shot count.

E. Quasi-static miscalibration model

A quantitative model of the pulse-angle contextual miscalibration errors (e.g. pulse interference effects) from the cryo-controller, interconnect, and package is difficult to achieve *a priori*. The system models discussed in Sec. S7 B have good qualitative and generic quantitative capture of such errors, and they certainly occur: when comparing to experimental results (see Sec. S8), we see that both the two-qubit gate errors and circuit errors are higher than would be expected when considering only noise sources described up to this point (e.g. T_2^*, N_{osc}, SPAM, and interconnect). Futhermore, the nature of the "speckle" in random sequences suggests that this error is fundamentally coherent (i.e. a result of repeatable miscalibration). We therefore claim that calibration error is a *dominant* error source in the performed multi-qubit experiments, and have added a single-parameter miscalibration ("miscal") error term to our model.

Arguably the simplest miscalibration model would be to add white noise on to each exchange pulse. However, we know from the pulsed exchange oscillation decay experiments (and the associated noise parameter N_{osc}) that the white noise of our pulses is very low. Instead, we introduce a quasi-static

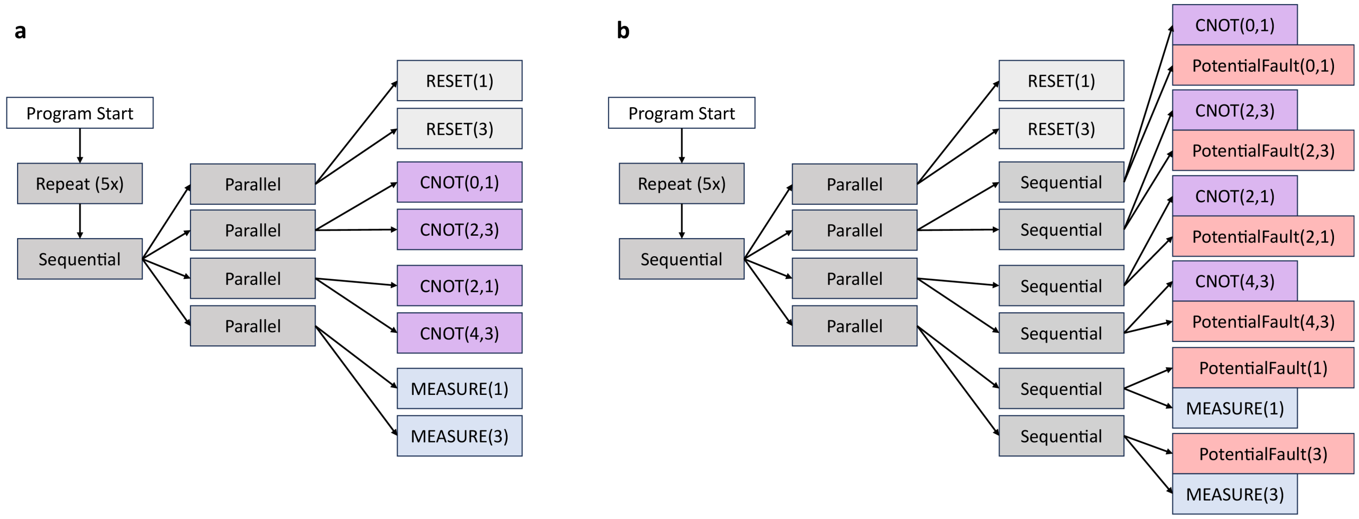

Supplementary Fig. S39. **Simple program example. a,** Example program represented in Squeaky as an AST before errors are inserted. **b,** The same program as in a but with potential faults inserted due to noise injection.

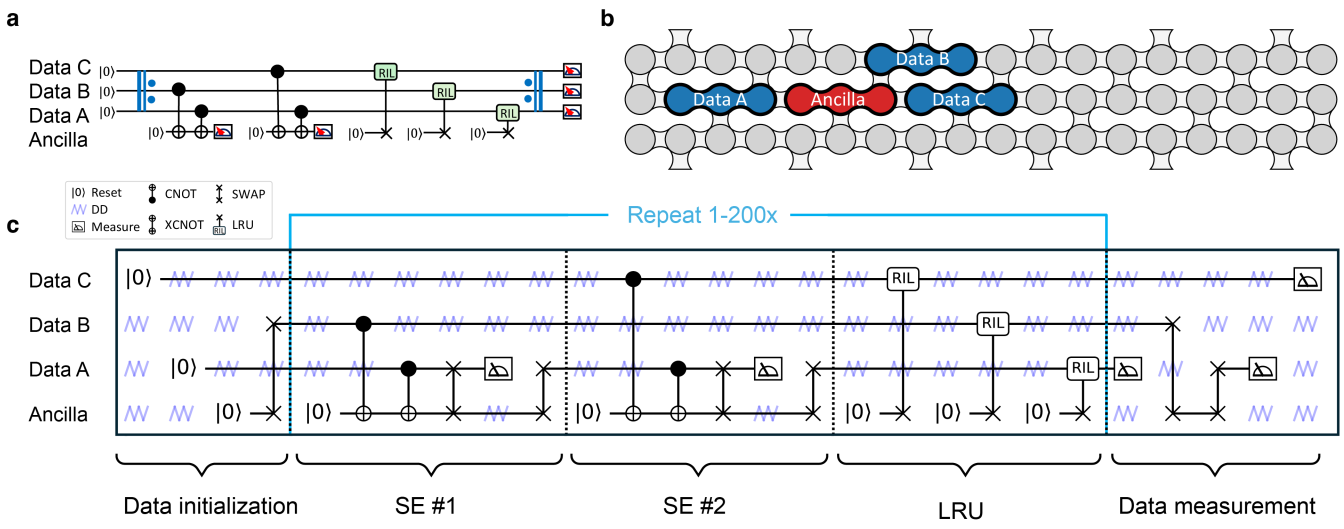

Supplementary Fig. S40. **Full [3,1,3] circuit and qubit layout a**, An idealized [3,1,3] circuit. **b**, A cartoon schematic of the distance-3 repetition code device with the qubit layout. **c**, The full circuit used specifying all SWAP (drawn as ×–×) and dynamical decoupling (drawn as purple resistor-like symbols) operations.

model of miscalibration. To implement it, we pick a global value miscalibration magnitude, parameterized as "$\Delta\theta/\theta$", a fractional error for each exchange. For every shot of the coherent spin simulation, we sample a random value from a Gaussian distribution centered at zero with standard deviation given by $\Delta\theta/\theta$ for each exchange axis a_i, i.e. a unitless number $\delta_m(i) = \mathcal{N}(0, \Delta\theta/\theta)$. This collection of random numbers is then used to linearly transform every exchange angle in the circuit:

$$\theta_j(i) \rightarrow [1 + \delta_m(i)] \times \theta_j(i), \tag{S14}$$

where j indexes time. In other words, to generate a single shot of the quasi-static miscalibration model, every pulse along a given axis will be scaled by the same relative magnitude, with that magnitude varying axis-to-axis. The quasi-static miscalibration model is time-agnostic and strongly axis-dependent.

In the following section, we will see the quasi-static miscal model is able to reproduce the experimentally observed error rates of two-qubit gates, syndrome detectors, and logical codes. However, it leads to larger probabilities of high-weight correlations in DEMs when compared to experiment. Discrepancies at high weight are not surprising; the physical processes originating from the cryo-controller that lead to miscalibration are contextual, whereas this error model is quasistatic. If these flavors of contextual miscalibration errors persist in future hardware, more elaborate miscal models may be warranted.

S8. MULTIQUBIT EXPERIMENTS

A. Device layout and qubit orientation

Alternative readout configuration

For the demonstrations in this work, an alternative readout configuration referred to as "forward DCS" is used. The readout dot charge sensors (DCS) are formed using the first row of plunger dots (PA3, PB3, etc., labeled in dark green), rather than the nominal measurement dots (MA1, MB1, etc.), because the shorter P-to-P pitch between rows improves charge sensitivity over the P-to-M pitch, and thus readout fidelity. To enable this style of measurement, intervening gates between the nominal bath gates (BA, BB, etc.) and the operating DCS measure dot are biased to a highly over-coupled regime, effectively extending the electron bath on both source and drain pathways to reach the DCS. Dots "flooded" in this manner are highlighted in yellow in Fig. S24. In the forward DCS configuration, as in the nominal configuration, the DCS operates in a many-electron regime. Modifications to the gate geometry and improvements to the measurement chain will render this technique obsolete going forward.

Qubit orientations

Exchange-only qubits are initialized by preparing a two-electron singlet and a third, uninitialized spin on three consecutive quantum dots. The exchange "axes" of a single EO qubit, corresponding to a pair of spins undergoing exchange, are referred to by the geometric axes which would result from a qubit Bloch sphere on the EO qubit. (We emphasize that this Bloch sphere is not that corresponding to the total spin of the EO qubit; that total-spin Bloch sphere would track the irrelevant gauge-degree of freedom). For the EO qubit, the Z axis refers to the pair of spins over which the singlet is initially prepared. The N axis refers to the axis between the middle spin and the initially uninitialized third spin. Quantum angular momentum recoupling rules fix this axis as $N = Z\cos(\phi) + X\sin(\phi)$, with $\phi = 2\pi/3$. We also refer to N and Z as Bloch-sphere π pulses about their corresponding axes, which in turn corresponds to complete swaps of corresponding spin pairs. These axis names also correspond, for any given EO qubit, with two exchange gates; for example XA5XA6 refers to a qubit in which the pair of dots surrounded by exchange gate XA5 defines the Z axis, and the pair of dots surrounded by exchange gate XA6 defines the N axis. With this notation established, a given set of three dots can equivalently be prepared in the ZN or NZ orientation, and two-qubit gates can be performed between any combination of two adjacent qubit orientations, given appropriate modifications to the pulse sequence. It is straightforward to tile our 18-qubit device such that each qubit's Z and N axes only interact with the Z and N axes, respectively, of adjacent qubits. For simplicity, this is our preferred configuration, and is used for all demonstrations in this work.

B. [3,1,3] demonstration

For the distance-3 code, four qubits are used. Qubit XB6XB5 is used as the ancilla, while XA5XA6, XC7XC8, and XC5XC6 are used as data qubits A, B, and C respectively. The full circuit for this experiment is displayed in Fig. S40c. In this configuration we only use "Data A" as our native measurement site during the bulk of the sequence, and perform the necessary swaps to initialize or measure the data qubits.

[3,1,3] noise characterization

The microscopic device parameters for the distance-3 repetition code were characterized by various experiments, and are summarized in Fig. S41. Singlet decay experiments were performed across all dot-pairs by performing series of swaps to and from the respective dot-sites and SPAM site, and measuring the oscillation frequency, ω and envelope decay T_2^*. We assume the dot-swap error is negligible with respect to magnetic error. For spin simulation, we use an average T_2^* for qubits of the constituent dot T_2^* (see Sec. S7 A). To derive a local magnetic field, we solve the linear system of equations $|A \cdot \mathbf{B}| = |\mathbf{W}|$ for B, where $\mathbf{W}$ is a vectorized list of all spin-pair oscillations (ω/γ_e), A is a matrix that tracks the relevant spins in each vectorized spin-pair, and (in this case) $\mathbf{B}$ is a vectorized list of the magnitude of the local magnetic field at each dot. The derived local magnetic field is treated as a static field throughout the spin simulation. Time-domain exchange oscillation experiments estimate the microscopic charge noise parameter N_{osc}. This value integrates all sources of system control noise and is a distinct quantity from that reported in the main text, which was dominated by intrinsic device noise. For budgeting purposes, we use this more inclusive *in situ* parameter. With envelope T_2^*, a static magnetic gradient, and system charge noise, we perform GAQQMap attribution (Sec. S4 D) and a complete randomized benchmarking simulation. Both methods estimated similar CNOT gate errors, but were lower than RB error rates reported from experiment. We include an ersatz quasi-static miscalibration error of 1.5% to account for contextual pulse miscalibration. This excess noise, referred to as "extrinsic" in the main text, is applied uniformly across the repetition code simulation and is sampled per-shot. For SPAM errors, we evenly split 1.1×10^{-3} between both initialization and measurement error.

[3,1,3] error attribution

In Fig. S42 we compare simulated and experimental performance of the distance-3 repetition code, using both the event simulator and the spin simulator. A first-order (linear) attribution is performed via the spin simulator by simulating

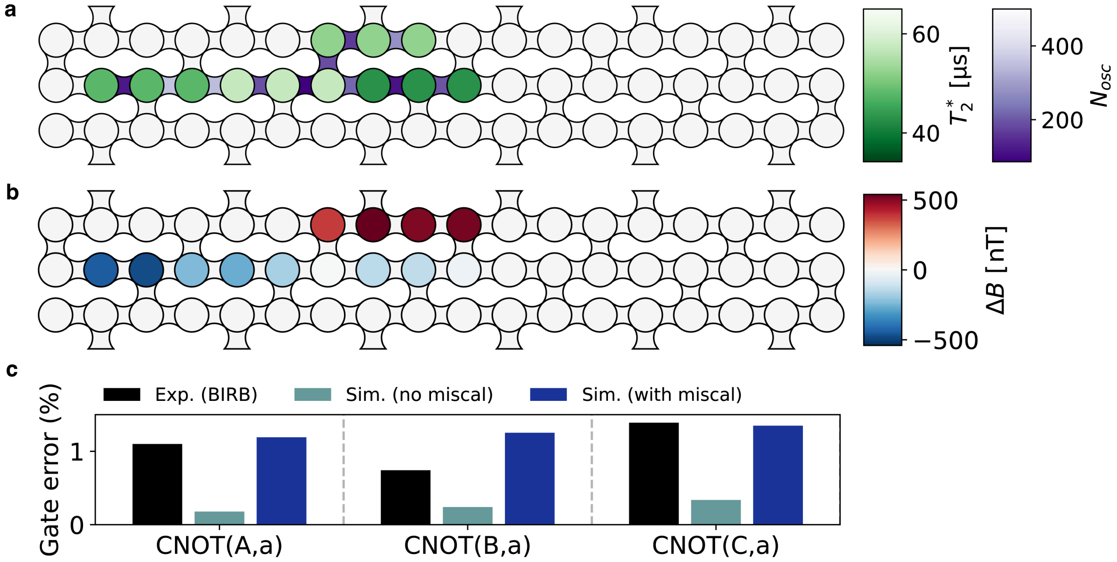

Supplementary Fig. S41. **Device noise characterization of the QPU used for the distance-3 repetition code. a,** Envelope T_2^* decay rates averaged by qubit and inverted per dot and charge noise parameter N_{osc} for each exchange axis. **b,** Estimated static magnetic gradient across dots with a global magnitude of 18 µT. Note this is measured with the integrated QPU and includes all associated system noise, as opposed to the data shown in the main text. **c,** Comparison of CNOT gate errors with ersatz miscalibration between simulation and experiment. CNOT(x,a) refers to the CNOT between Data x =A,B, or C (see Fig. S40**b**) and the single ancilla qubit (a).

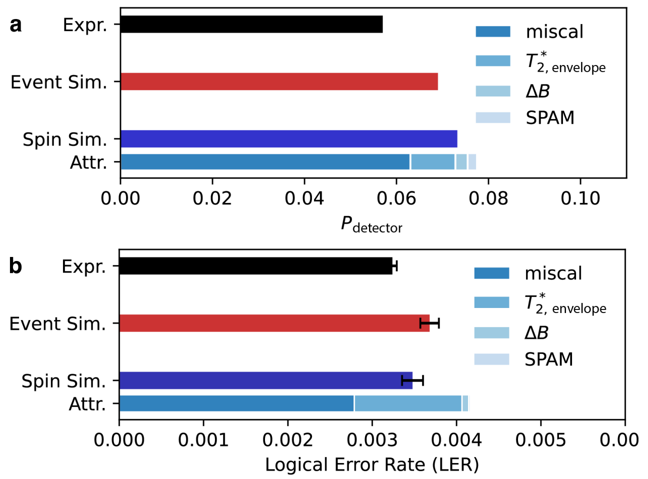

Supplementary Fig. S42. **Comparison between experiment (black), event simulator (red), and spin simulator (dark blue) for the distance-3 repetition code.** Also included is a linear error-attribution for the spin simulations (light blues). Attribution of N_{osc} error was evaluated but not plotted because it was negligible. **a,** A comparison of the average detector rate, P_{detector}, for [3,1,3] circuits with 10 rounds. **b,** A comparison of the logical error rate by way of an exponential fit to the round-dependence of the logical error probability. Decoding was performed with the software package pymatching [44].

the repetition code under the influence of only a single noise source at a time. We individually evaluate the impact of T_2^*, ΔB, SPAM, and quasi-static miscal. Charge noise, characterized by N_{osc}, is also included but negligible and therefore not plotted. To get a full picture of how these noise sources contribute to circuit error, one must also compute the non-linear (quadratic, cubic, etc.) relationships, but for simplicity we do not fully explore that here. We find that this first-order attribution model slightly overestimates the total error rate produced by a holistic simulation but still provides a reasonable guide. Figure S42 applies this analysis to two metrics of circuit performance: the first metric is the average probability of a syndrome detection of a local error, P_{detector} (also referred to as the detector event fraction, or DEF); the second metric is the logical error rate ϵ_L which is fitted to $[1-(1-2\epsilon_L)^n]/2$ when observing the decoded error probability per round n. In the distance-3 repetition code, we use pymatching [44] to construct a minimum-weight perfect matching decoder, assuming a uniform depolarizing error channel given the error rates of CNOTs, dynamic decoupling exchanges, etc. In both metrics, the most dominant first-order noise contribution is the ersatz quasi-static miscalibration, followed by the nuclear magnetic noise term T_2^*.

[3,1,3] DEM analysis

A DEM analysis of the distance-3 repetition code with five rounds of syndrome measurement is shown in Fig. S43. The excellent agreement between DEMs generated from simulation and experiment for weight-1 and weight-2 events shows that our modeling workflow is doing a good job of predicting simple qubit event errors. Indeed, in panel c, disagreements only

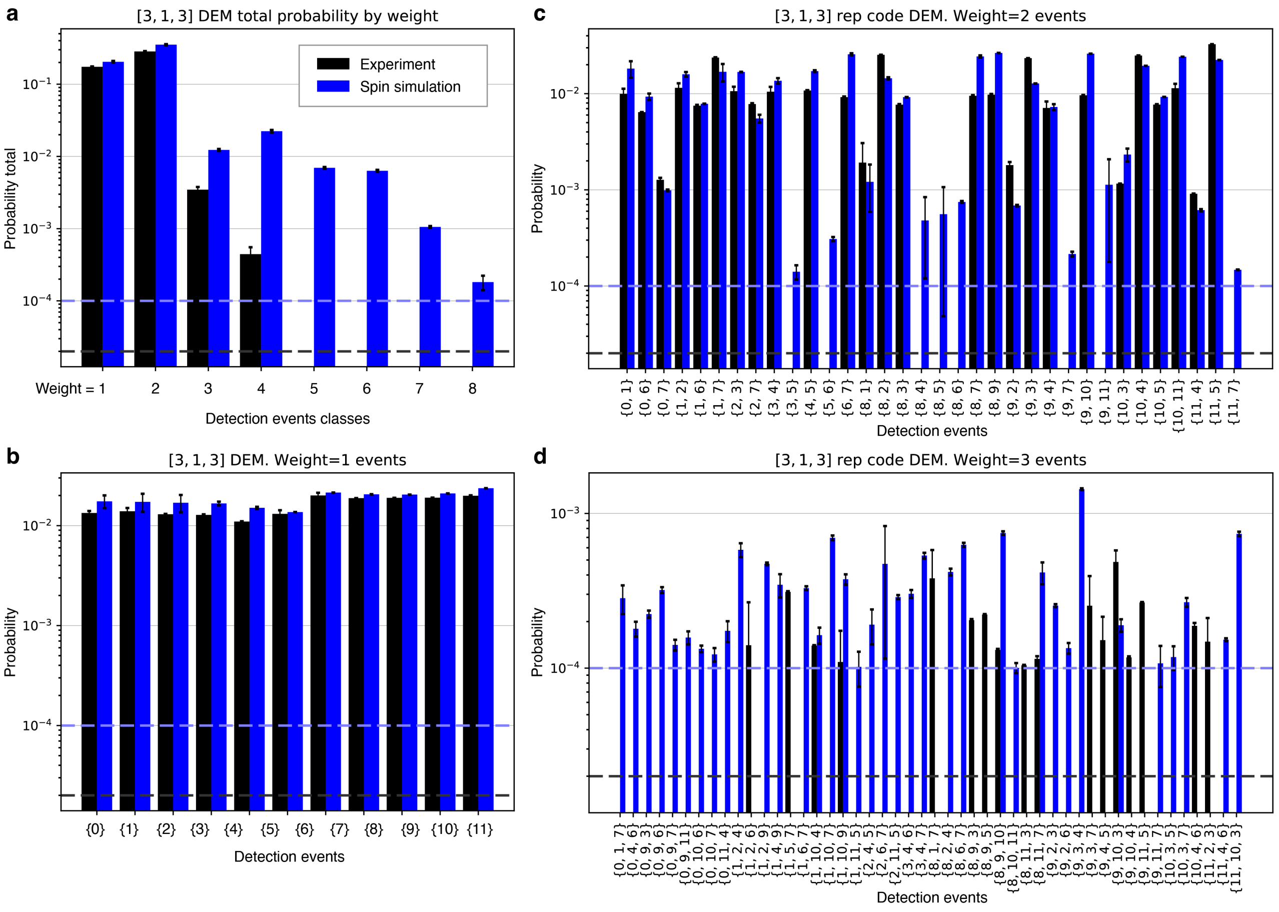

Supplementary Fig. S43. **DEMs constructed from experiment (black) and pyQUEST simulation (blue) data for the distance-3 repetition code with 5 rounds of syndrome measurement.**. Error bars represent each probability's σ from shot-noise uncertainty. This is a DEM of size $N = 2 \times 6 = 12$. **a,** Comparison of total probabilities for each weight-class of error events. **b,** Comparison of error probabilities event-by-event for weight-1 errors. **c,** Comparison of error probabilities event-by-event for weight-2 errors. **d,** Comparison of error probabilities event-by-event for weight-3 errors. Dashed lines correspond to $1/N_{\text{shot}}$ with $N_{\text{shot}} = [5\times10^4, 1\times10^4]$ for experiment and pyQUEST. Overall, experiment and simulation track well when comparing events in aggregate, especially for lower weight events with pyQUEST overestimating high weight errors.

occur when the simulated or experimental probabilities are near their shot-limit (blue and black dashed lines, respectively).

The simulator overestimates high weight errors compared to experiment. This is not surprising: the ersatz miscalibration used in simulation is an axis-distinct, uniform-in-time error, and as such is capable of creating high-weight events. For example, if axis XA5 is very noisy in one simulated shot, then one would expect many repeated errors on data qubit A. In experiment, the contextual pulse miscalibration in the real cryo-controller is a non-uniform-in-time in error, and is likely less axis-dependent (e.g. XA5 and XC5 may experience similar errors in similar contexts), leading to mostly low-weight errors.

C. [5,1,5] demonstration

Seven qubits are used in the distance-5 demonstration, as shown in the simplified version of the circuit in Fig. S44a. The layout of the device is depicted in Fig. S44b: Qubits XA6XA5 (corresponds to the gate names of Z/N axes) and XC6XC5 are used as ancilla 1 and 2, while XA9XA8, XB5XB6, XB8XB9, XC9XC8, and XD5XD6 are used as data qubits A-E respectively. All qubits are coupled to their neighbors through the X and Y gates shaded in light blue, except Data A and C due to a known issue with XB7 due to a daughterboard flaw. We can perform native SPAM at Ancilla 1, 2, and Data B (not used, however), whereas we must add swaps to initialize and measure Data A, C, D, and E.

The detailed circuit used in the repetition code is shown in Fig. S44c, where we are now showing all of the qubit-level SWAPs needed for initialization, certain entangling operations, measurement, and dynamical decoupling that is present throughout the entire circuit.

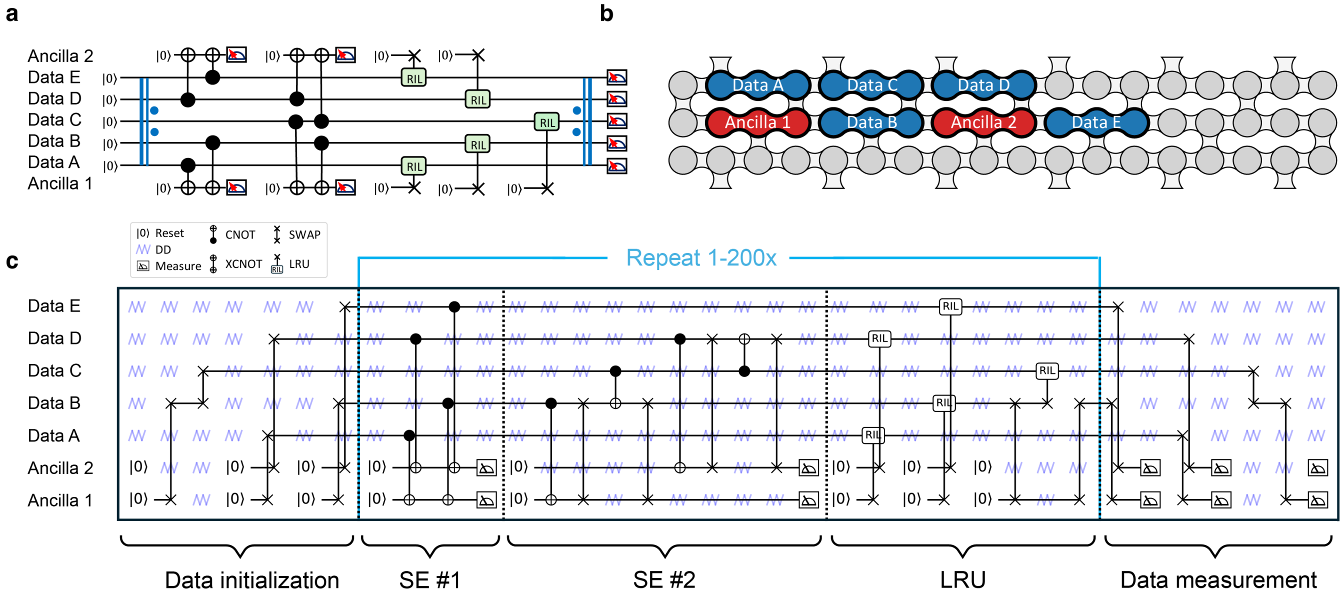

Supplementary Fig. S44. **Full [5,1,5] circuit and qubit layout. a,** The idealized distance-5 repetition code circuit shown in the main text, reproduced here. **b,** A cartoon schematic of the device used for the distance-5 repetition code with the qubit layout overlaid. **c,** The full circuit used specifying all SWAP (drawn as ×–×) and dynamical decoupling (drawn as resistor-like symbols) operations.

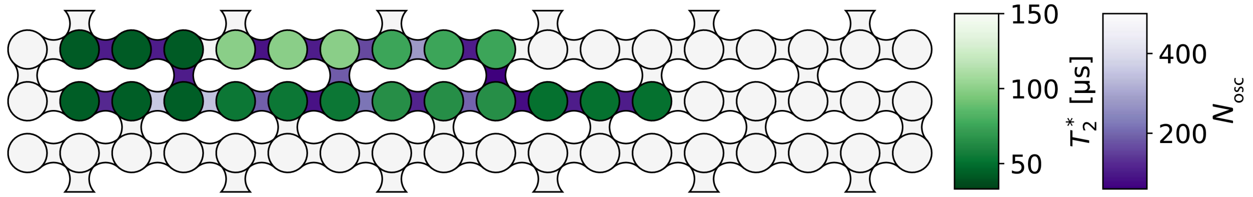

Supplementary Fig. S45. **Device noise characterization for distance-5 repetition code.** Effective T_2^* decay rates (accounting for static magnetic gradients) are averaged by qubit and inverted per dot. Charge noise parameters N_{osc} are measured for each exchange axis. Note this is measured with the integrated QPU and includes all associated system noise, as opposed to the data shown in the main text.

[5,1,5] noise characterization

The dot-level device parameters for the distance-5 repetition code were characterized in a similar fashion as the distance-3 repetition code measurement, and are summarized in Fig. S45. Instead of measuring singlet return probability of all possible pairs, as was done in the distance-3 setup, we only measure local qubit-defined singlet return probabilities of the three possible dot combinations. Additionally, due to low frequency oscillations that we attribute to static magnetic field gradients, we combine the envelope and oscillation into one effective first-order parameter given by $(T_2^*)^{-2} = (T_{2,\,\mathrm{env}}^*)^{-2} + \omega^2/2$. A global static magnetic field of 160 μT was measured via dynamical decoupling spectroscopy [35]. Time-domain exchange oscillation experiments estimate the microscopic charge noise parameter N_{osc}. We include an ersatz quasi-static miscalibration error of 1.5% to account for contextual pulse miscalibration, as described in Sec. S7 E. As we will discuss in the next section, one of the two SPAM sites provided unreliable and sometimes correlated measurements. Given this uncertainty, we assume a SPAM error of 1.2×10^{-3} for both sites, which we evenly split between both initialization and measurement error.

Spikes in detection events

One particular oddity we observe in the detector error fraction vs. time data (Fig. S46a, running the experiment continuously rather than for a fixed number of syndrome rounds) is the presence of spikes in detection event fraction that appear to last for several rounds. Focusing on a single spike and separating by detector (Fig. S46b), we see that detectors 1 to 3 show highly elevated errors, whereas detector 4 appears largely unaffected. We estimate the length of a spike event by computing when the event fraction deviates by 1.4826×MAD (median absolute

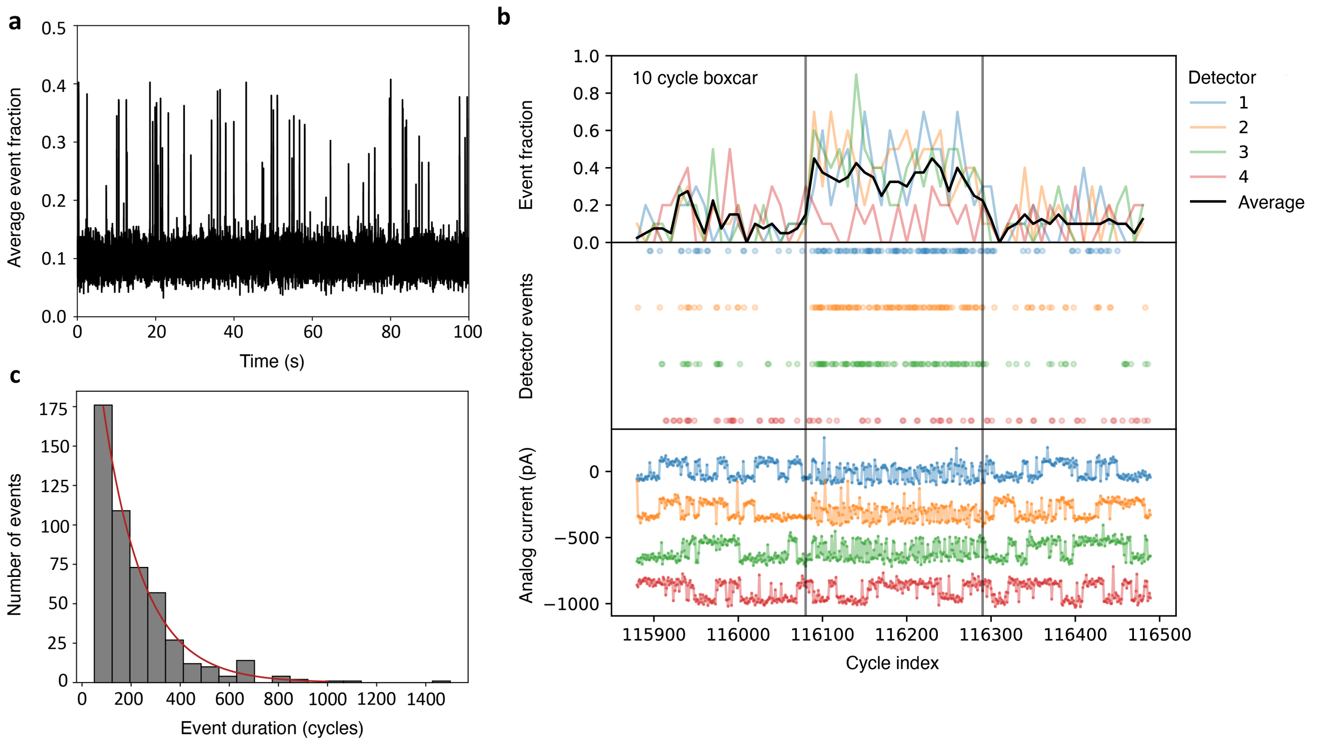

Supplementary Fig. S46. **[5,1,5] event fraction spikes. a,** Continuously running distance-5 syndrome extraction. The detector event fraction averaged over all detectors with a 100-cycle boxcar filter. **b,** A zoomed in view of about 600 cycles. The top subfigure displays a 10-cycle boxcar filter of the individual detector events per cycle (middle subfigure). The bottom subfigure shows the raw current values measured for each detector, which are ultimately converted to a 0 or 1 with a predefined threshold and finally a detection event if the measurement outcome flips between rounds. **c,** Histogram of number of spike events binned by duration, well fit by an exponential with a 164 cycle (14 ms) time constant.

deviation) to mark the start and end of a particular spike event. We can then plot the distribution of durations, and we see that it is well described by an exponential with a 164 cycle (14 ms) timescale.

So-called p_{ij} plots can give insight into what type of errors these spike events caused [45]. In Fig. S47, we see that by filtering out shots containing an identified spike event, the large red background covering detectors 1-3 is also removed. This is consistent with some event causing all three of those detectors simultaneously having elevated event fractions due to SPAM errors. However, we do notice that there exists a space-like correlation between detector 1 and 3 that remains. We suspect the same microscopic event that caused the SPAM errors is also the source of these correlations, but further investigation is required to understand the origin.

We did not observe this phenomenon in the [3,1,3] or [[4,2,2]] demonstrations.

[5,1,5] DEM analysis

We can use DEMs to further analyze the effects of filtering out spurious or anomalous data. As described above, the distance-5 repetition code consistently saw measurement spikes that indicated highly correlated errors (as seen in the p_{ij} matrix). Using a DEM analysis, we see that filtering out those measurement spikes suppresses the high-weight error probabilities in aggregate (Fig. S48). Similarly to the [3,1,3] DEM analysis, the simulator's use of the ersatz miscalibration model leads to an overestimation of events of weight-3 and higher. The overall similarities between the [3,1,3] and [5,1,5] DEM analyses is also a good indication that our intrinsic and extrinsic error sources are well characterized.

D. [[4,2,2]] demonstration

For the [[4,2,2]] demonstration, six qubits were used, from the top and middle rows of an 18 qubit device (Fig. S49b) Qubits XB6XB5 and XC5XC6 are used as the ancilla and flag qubits, while XA5XA6, XB9XB8, XA8XA9, and XD6XD5 are used as data qubits A-D respectively. Data qubits A, C, and the flag qubit are oriented with axes ZN from left to right, while data qubits B, D and the ancilla qubit are oriented with axes NZ from left to right. For better performance on this particular device, the circuit is compiled to use a single measurement site without consequence to error propagation.

Compared to the repetition code demonstrations, the

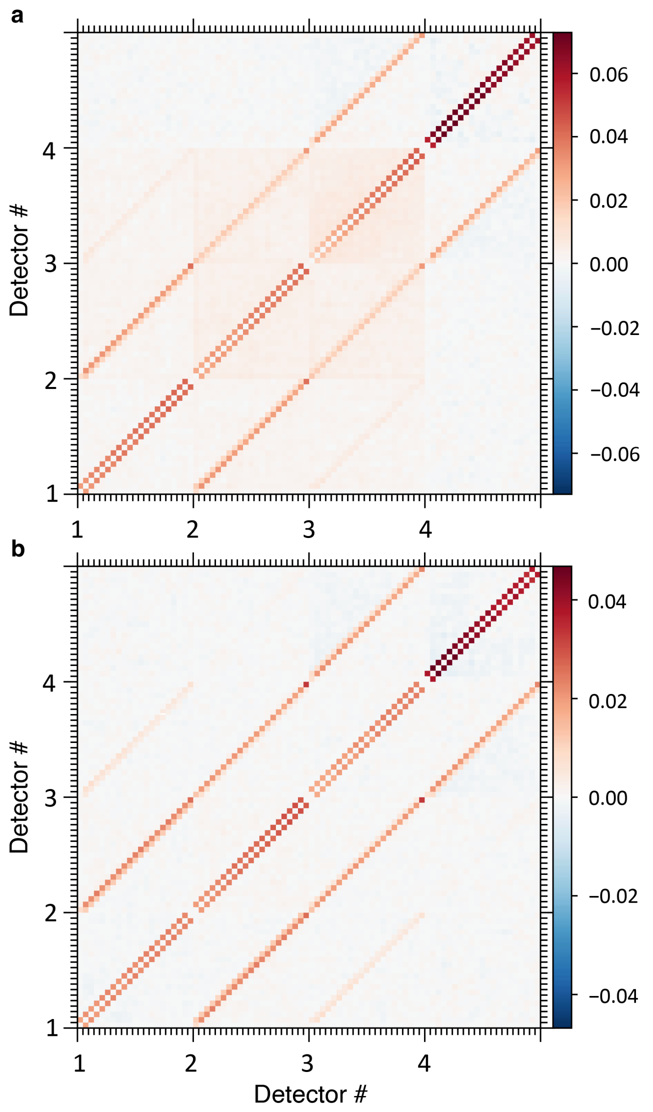

Supplementary Fig. S47. **[5,1,5] correlations with and without spike events. a,** p_{ij} of the 20-round distance-5 circuit. **b,** p_{ij} plot after filtering out shots for which a spike event occurred.

[[4,2,2]] demonstration was performed in a system using a later version of the cryo-controller but with comparable electronic performance, a later revision of the daughterboard with improved signal integrity, and custom transimpedance amplifiers (Sec. S3 D). With respect to quantum performance, this system exhibited better one-qubit and two-qubit error rates and similar SPAM errors.

The [[4,2,2]] quantum circuit is shown in Fig. S49a and c. After all four data qubits are initialized, successive rounds of syndrome extraction and leakage removal are applied. Each round consists of an *XXXX* parity check, a *ZZZZ* parity check, and leakage reduction units (LRUs). The parity check measurements of the ancilla are each immediately preceded by a measurement of the flag qubit, which validates the ancilla outcome. When the data qubits are initialized to logical states in the X basis, the order of the Z and X parity checks is reversed to ensure that the first parity check efficiently projects the prepared state into the code space. The quantum operations are performed sequentially, with dynamical decoupling pulses [46] on all idle qubits interleaved with operational pulses.

[[4,2,2]] noise characterization

For the [[4,2,2]] experiment, an unexpected temperature cycle of the device occurred before full qubit characterization data could be obtained. The microscopic magnetic noise parameters T_2^* were characterized by experiment, and are summarized in Fig. S50. Dynamical decoupling spectroscopy, used as an *in situ* measure of the local magnetic field for each qubit, showed an average global magnetic field of 20 µT and minimal magnetic gradients. To capture any small magnetic gradients not visible to this technique, we used effective T_2^* values instead of the envelope of the fit in our simulations.

For the microscopic charge noise parameter N_{osc}, we used a placeholder value of 200 for every axis. This is a pessimistic number (see main text Fig. 3), which likely overestimates the amount of charge noise in the device. But it is a necessary precaution, as no N_{osc} measurements were taken in the final tuned-up state of the device before the fridge was temperature cycled.

For SPAM errors, based on singlet/triplet readout histograms we assigned a low initialization error of $1.2{\times}10^{-5}$ in the coherent spin simulations. Assignment fidelity experiments were run multiple times on the [[4,2,2]] readout site and the best performance was about 1.2% infidelity, with triplet readout often better than singlet readout. However, there was a slow drift in the optimal SPAM threshold current, and some assignment fidelity experiments showed closer to 1% error for triplet readout and >2% error for singlet readout. Due to the long duration of the [[4,2,2]] experiment and the slow drift, we used this low-water mark as the measurement fidelity in the spin and event simulations.

Unlike the [3,1,3] and [5,1,5] experiments, we did not calibrate the ersatz miscal parameter to the one-qubit and two-qubit BIRB values. Instead, we ran full circuit simulations with the above magnetic, charge, and SPAM noise parameters, and added a quasi-static miscalibration value (0.9%) that reproduced experimental detector probability rates. We then performed GAQQmap attribution (Sec. S4 D) of the two-qubit gates in our spin simulator, and used those error rates in the event simulator. This led to excellent agreement in circuit throughput and fidelity predictions, and improved the agreement in two-qubit gate error rates (in three out of the four gates in which that comparison could be performed, see Fig. S51). Note that the simulated error rates without any miscalibration added are all below the experimentally observed two-qubit BIRB errors. This highlights that, just like in the system used for the repetition code experiments, the intrinsic magnetic and charge noise of the device are not dominant error sources for

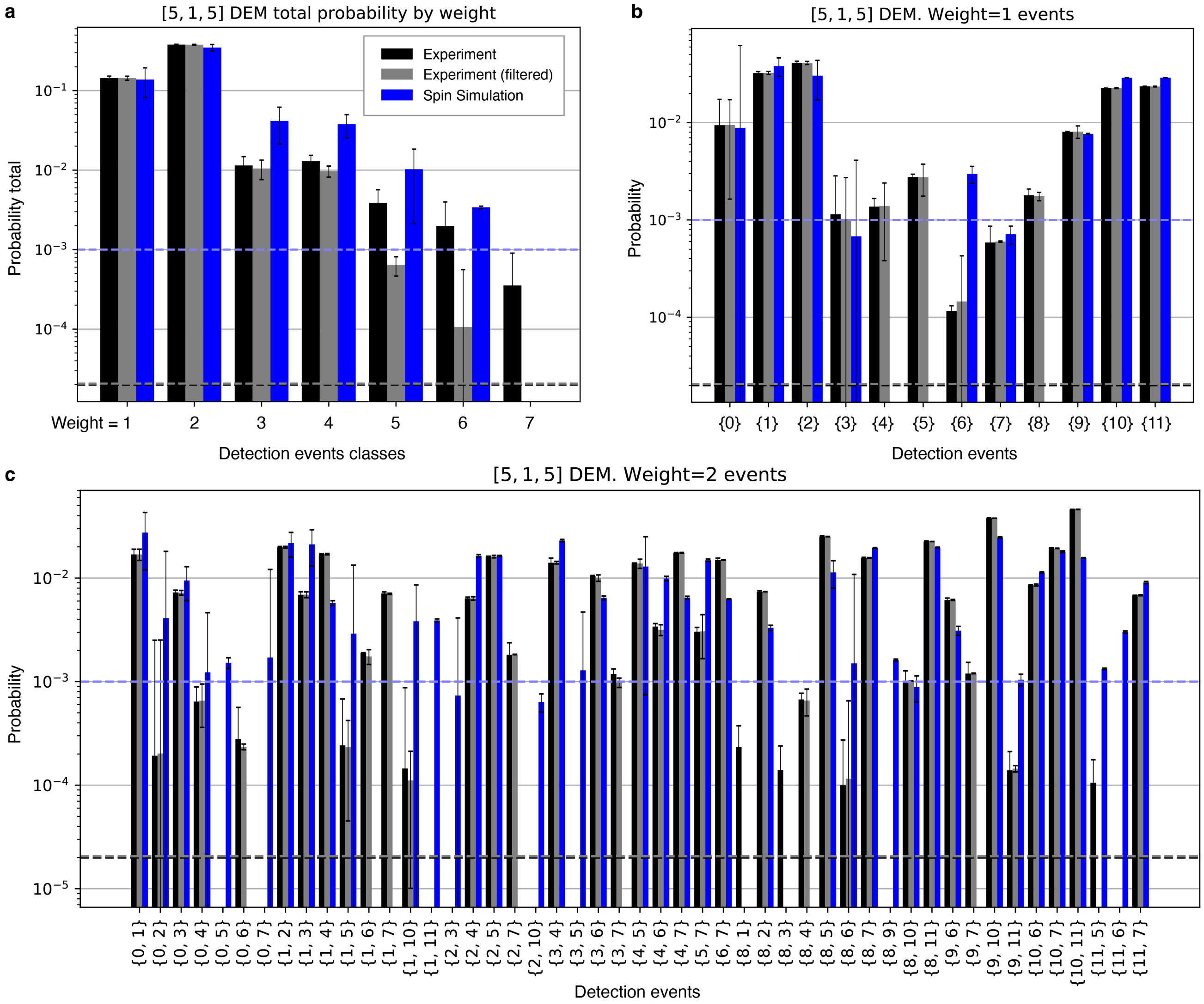

Supplementary Fig. S48. **DEMs constructed from experiment (black), filtered experiment (grey), and pyQUEST simulation (blue) data for the distance-5 repetition code with two rounds of syndrome measurement.** This is a DEM of size $N = 4 \times 3 = 12$. **a,** Comparison of total probabilities for each weight-class of error events. **b,** Comparison of error probabilities event-by-event for weight-1 errors. **c,** Comparison of error probabilities event-by-event for weight-2 errors. Dashed lines correspond to $1/N_{\text{shot}}$ with $N_{\text{shot}} = [5\times10^4, 4.851\times10^4, 1\times10^3]$ for experiment, filtered experiment, and pyQUEST. Overall, we observe that filtering out the measurement spikes in the experimental data suppresses high-weight errors and that the pyQUEST simulations on aggregate overestimate high weight errors.

TABLE S1. **Codespaces and codewords of the [[4,2,2]] code.** The sixteen (unnormalized) basis states of the four data-qubits are listed in parenthesis from (1) to (16), and are grouped into sets of four codewords for the four codespaces [A] through [D]. The four codespaces are distinguished by stabilizer eigenvalues (Z_{1234} and X_{1234}, i.e. Z-check and X-check ancilla outputs in the absence of errors).

	**Codespace A**	**Codespace B**	**Codespace C**	**Codespace D**
(Z_{1234}, X_{1234})	$(1, 1)$	$(1, -1)$	$(-1, 1)$	$(-1, -1)$
$\lvert 00\rangle_L$	(1) $\lvert 0000\rangle + \lvert 1111\rangle$	(5) $\lvert 0000\rangle - \lvert 1111\rangle$	(9) $\lvert 0001\rangle + \lvert 1110\rangle$	(13) $\lvert 0001\rangle - \lvert 1110\rangle$
$\lvert 01\rangle_L$	(2) $\lvert 0011\rangle + \lvert 1100\rangle$	(6) $\lvert 0011\rangle - \lvert 1100\rangle$	(10) $\lvert 0010\rangle + \lvert 1101\rangle$	(14) $\lvert 0010\rangle - \lvert 1101\rangle$
$\lvert 10\rangle_L$	(3) $\lvert 0101\rangle + \lvert 1010\rangle$	(7) $\lvert 0101\rangle - \lvert 1010\rangle$	(11) $\lvert 0100\rangle + \lvert 1011\rangle$	(15) $\lvert 0100\rangle - \lvert 1011\rangle$
$\lvert 11\rangle_L$	(4) $\lvert 0110\rangle + \lvert 1001\rangle$	(8) $\lvert 0110\rangle - \lvert 1001\rangle$	(12) $\lvert 1000\rangle + \lvert 0111\rangle$	(16) $\lvert 1000\rangle - \lvert 0111\rangle$

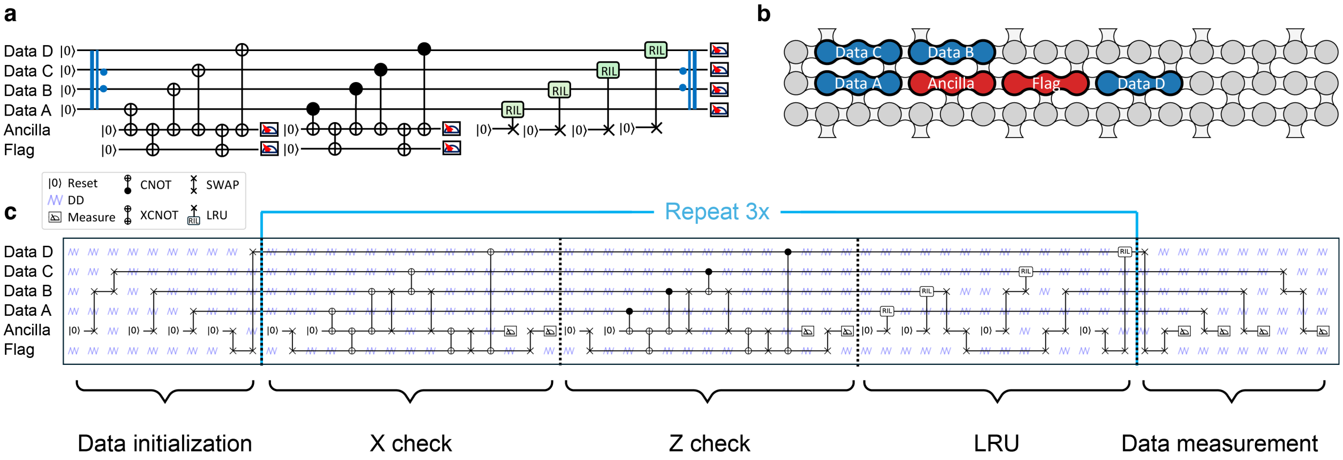

Supplementary Fig. S49. **Full [[4,2,2]] circuit and qubit layout. a,** The idealized [[4,2,2]] circuit. **b,** A cartoon schematic of the [[4,2,2]] device with the qubit layout. **c,** The full [[4,2,2]] circuit used to specify all SWAP (drawn as ×–×) and dynamical decoupling (drawn as resistor-like symbols) operations.

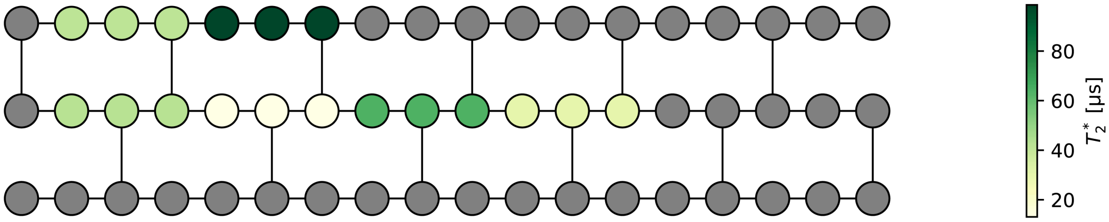

Supplementary Fig. S50. **Map of the effective T_2^* values used in simulation of the [[4,2,2]] circuit.** For each software-defined qubit, the three unique dot-pair T_2^* values are averaged into a single qubit-wide value.

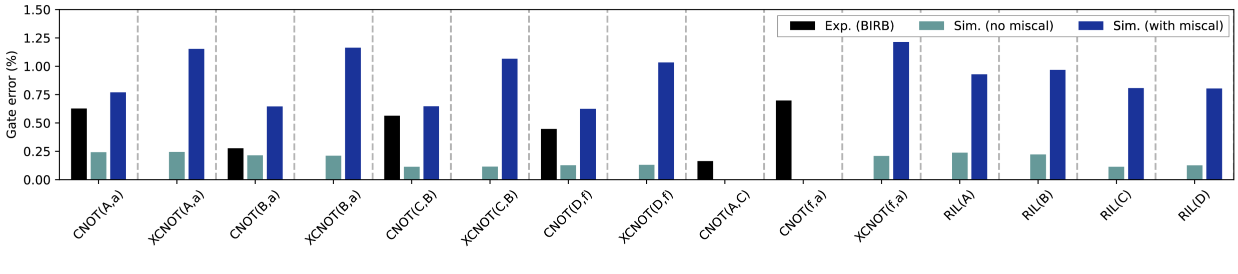

Supplementary Fig. S51. **Comparison of experimental two-qubit gate errors (obtained via BIRB) and simulated two-qubit gate errors (obtained via GAQQmap, Sec. S4 D) in the [[4,2,2]] demonstration**. The data qubits are listed as A, B, C, D, the ancilla qubit as "a" and the flag qubit as "f" (as defined in Fig. S49). Not all gates used in the circuit were experimentally characterized (i.e. missing black bars), and two CNOTs were experimentally characterized even though they did not occur in the circuit [CNOT(A,C) and CNOT(f,a)].

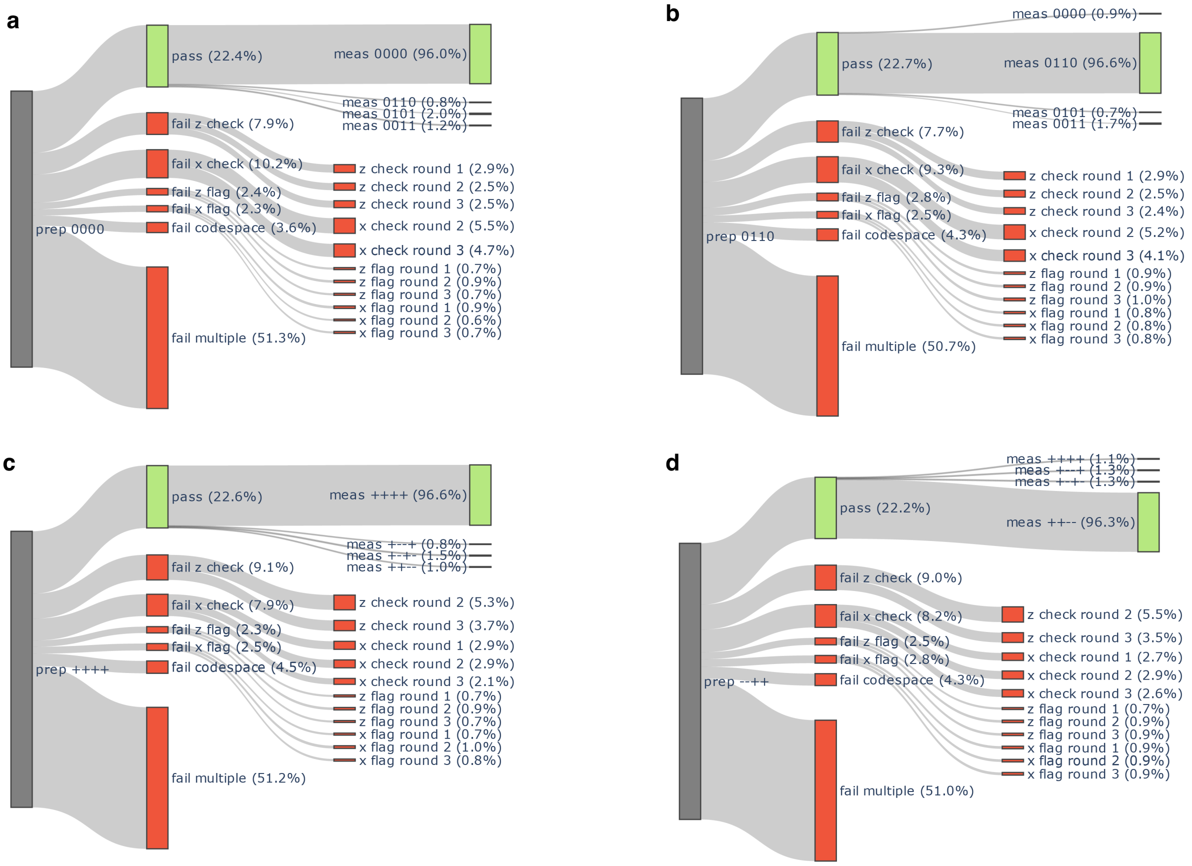

Supplementary Fig. S52. **Classification of the 10,000 experimental shots of the [[4,2,2]] circuit for each prepared state**. The percentages associated with the "pass" state of the circuit throughput check (green) are normalized with respect to the number of passing shots, while all other rates (red) are normalized with respect to 10,000 shots. **a,** Prepare $|0000\rangle$. **b,** Prepare $|0110\rangle$. **c,** Prepare $|{+}{+}{+}{+}\rangle$. **d,** Prepare $|{-}{-}{+}{+}\rangle$.

two-qubit gates.

[[4,2,2]] initialization and post-selection

We prepared four different initial states on the data qubits: $|0000\rangle$, $|0110\rangle$, $|{+}{+}{+}{+}\rangle$, and $|{-}{-}{+}{+}\rangle$. The first two states use a circuit which first performs an X-stabilizer, then measures the final data qubits in the Z-basis (native to our architecture). The last two states use a circuit which first performs a Z-stabilizer, then measures the final data qubits in the X-basis (by using a single-qubit H gate before the measurement). These states do not begin in a specific codespace, but rather a superposition of A and B (C) for the Z-basis (X-basis) initial states, see Table S1. Therefore, the first stabilizer round will project the initial state into one of *two* possible states, one in each of the possible codespaces. For example, the initial state $|0110\rangle$ will project either into state 4 or into state 8, depending on the "sign" assigned by the X-check (with an equal chance of either outcome).

Without a flag qubit, the [[4,2,2]] code can detect any weight-1 event (i.e. single-qubit error) on the data qubits. However, when a single Z fault on the ancilla qubit occurs between the second and third entangler of either stabilizer, it propagates an error (X or Z, depending on the stabilizer) onto two data qubits. This changes the logical state, but keeps the total Z or X parity the same and is therefore an undetectable weight-1 error in our circuit. To make our circuit robust to *all* single qubit errors, we therefore add an additional ancilla, called the "flag", that entangles our original ancilla qubit around this dangerous location. When a Z error occurs on the ancilla halfway through a stabilizer round, the flag qubit will detect it, improving the logical fidelity of the circuit.

To quantify the error-detecting capability of the [[4,2,2]] code, we post-select only the experiment shots where no error was detected. Each such shot must meet four requirements:

1. *All flag measurements are* $|0\rangle$.

2. *All X syndrome measurements are identical and consistent with the targeted codespace.*

3. *All Z syndrome measurements are identical and consistent with the targeted codespace.*

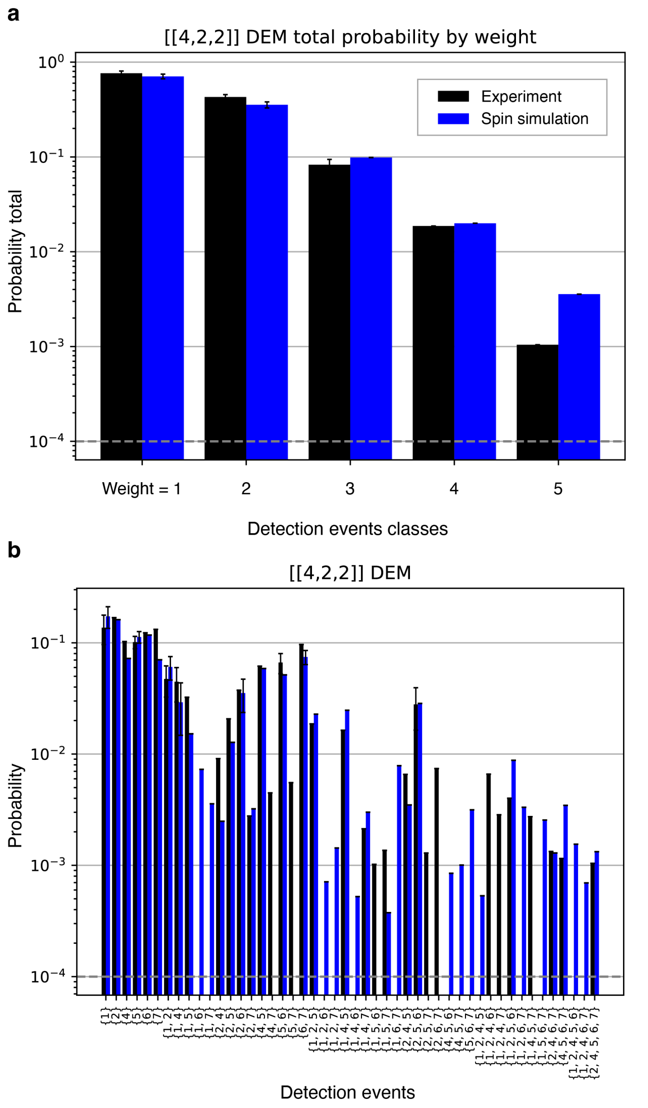

Supplementary Fig. S53. **DEMs constructed from experimental (black) and simulated (blue) data for the [[4,2,2]] code with 3-rounds of syndrome detection.** This is a DEM of size $N = 2{\times}4 = 8$. **a,** Comparison of total error probabilities for each weight-class of error events. **b,** Comparison of error probabilities event-by-event. Grey dashed line corresponds to $1/N_{\text{shot}}$, N_{shot}=10,000. Overall, experiment and simulation track well when comparing events in aggregate, especially for lower weight events with pyQUEST overestimating high weight errors.

4. *The parity of the measured data qubits is consistent with the targeted codespace.*

For requirement 4, we use parities computed from data-qubit measurements to determine if an error occurred. This is analogous to using the measurement of the data qubits in a repetition code to infer errors that occur during or after the last round of syndrome extraction.

If any of the above checks fail, an error is considered to have occurred. For example, if we started with the state $|0000\rangle$, the four requirements might be violated in the following ways:

1. *The flag measurement during the second X stabilizer is triggered.*

2. *The first X stabilizer is* -1 *and the second X stabilizer is 1.*

3. *The first Z stabilizer is* -1 *(*$|0000\rangle$ *must live in a* $Z_{1234} = 1$ *codespace).*

4. *The final measured state is* $|0001\rangle$.

The "circuit throughput" is defined as the probability that a shot passes all of the post-selection rules.

From these post-selected shots, we then check if the final measured state is in agreement with the state that was prepared, represented by two correct states. For example, if we prepared the state $|{-}{-}{+}{+}\rangle$ both the state $|{-}{-}{+}{+}\rangle$ and $|{+}{+}{-}{-}\rangle$ are considered a success. The "circuit fidelity" is then defined as the probability that a post-selected shot agrees with the prepared state. Figure S52 summarizes the throughput and fidelity outcomes of all 10,000 shots of each prepared state. Shots in which multiple post-selection rules were violated were only coarsely categorized, for figure simplicity.

[[4,2,2]] DEM analysis

Directly comparing DEMs constructed from experimental data and simulation data event-by-event we find discrepancies, especially as the weight increases. For example, in Fig. S53**a** the weight-1 [[4,2,2]] events between experiment and simulation match up decently well but diverge as the weight increases above weight-3 because of the ersatz miscalibration model used by the simulator (as discussed in the [3,1,3] and [5,1,5] DEM analysis sections as well). This uniform DEM behavior between the classical codes ([3,1,3] and [5,1,5]) and a quantum-error detecting code ([[4,2,2]]) further reinforces our confidence in the quality of our qubit error characterization approach.

One topic not directly addressed in our DEM analysis is a description of what types of events leakage creates. This is a rich theoretical topic and is critical to understanding the scalability of the exchange-only architecture. We have found no evidence that leakage (fully present in both simulation and experiment) is adversely affecting the DEM analysis. This is likely thanks to our generous use of RIL gates. Future work studying leakage in a quantum code would be a valuable effort. For example, specific RIL gates can be removed from a circuit and any clear DEM changes could be analyzed from a leakage-spreading perspective. Initial experiments doing just this were performed, but further analysis is needed.

Members of HRL Quantum Team and Collaborators

Michael Abraham[1], Edwin Acuna[1], Tower S. Adams[1], Moonmoon Akmal[1], Matthew R. Alfaro[1], I. Alvarado[1], Jacob Amontree[1], Carter Andrews[1], Reed W. Andrews[1], Michael Antcliffe[1], André R. Asencio[1], Ryan M. Avila Batres[1], Cynthia D. Baringer[1], David W. Barnes[1], Katherine M. Beech[1], Russell G. Blakey[1], Zachery T. Bloom[1], Aaron J. Bluestone[1], Jacob Z. Blumoff[1], Matthew G. Borselli[1], Koel A. Bose[2], Brydon Boyd[1], Jacob T. Boyer[1], Teresa L. Brecht[1], Christopher C. Brough[1], Rex A. Brown[1], Steven L. Brown[1], Tyler A. Cain[1], John B. Carpenter[1], Stephen Carr[1], Faustin W. Carter[1], Mitchell Casanova[1], Jacob L. Chambers[1], Matthew D. Chambers[1], Khamsorn L. Chanthavong[1], James M. Chappell[1], Rhian Chavez[1], Kevin C. Chen[1], Peter S. Chen[1], Maxwell D. Choi[1], Krishna Choudhary[1], Matthew N. H. Chow[1], Justin E. Christensen[1], Aaron M. Chronister[1], Andrew M. Clapper[1], Abigail A. Coker[1], Michael D. Cornelius[1], Albert E. Cosand[1], Ian T. Counts[1], Edward T. Croke[1], Gregory M. Crosswhite[1], Adam Dally[1], Erik S. Daniel[1], Tuan A. Dao[2], Dominic Daprano[1], Tiffany Davis[1], Neha Deshpande[2], Rachel S. Dey[1], D. Scott Diamond[1], Claire E. Dickerson[1], J. P. Dodson[1], James B. Dragan[1], Marc Dvorak[1], Lisa F. Edge[1], Charles R. Elliott[1], Kenneth R. Elliott[1], Kevin Eng[1], Jacob Fast[1], Colin P. Feeney[1], David J. Fialkow[1], Dylan H. Finestone[1], Micha N. Fireman[1], Bryan H. Fong[1], Trevor M. Fowler[1], Sean Frazier[1], Kiera L. Fuller[1], Christina A. C. Garcia[1], Kacy L. Garstka[1], Kara C. Garvey[1], Zachary A. Geiger[1], Galen R. Gledhill[1], Caleigh M. Goodwin-Schoen[1], Joseph L. Goralka[2], Bradley W. Greene[2], Hrayr K. Gurgenian[1], Sieu D. Ha[1], Wonill Ha[1], Nathanial R. Hapeman[1], Brooke M. Hardesty[1], Jim W. Harrington[1], Patrick M. Harrington[1], Thomas R. B. Harris[1], Ben M. Harrison[1], Anthony T. Hatke[1], Robert R. Hayes[1], Kevin He[1], Raul Hernandez Garcia[1], Ryan M. Hickey[1], Jocelyn Hicks-Garner[1], Alex Hirman[1], Donald A. Hitko[1], David Ho[1], Holland Y. Ho[2], Vinh S. Ho[1], nathan holman[1], Adam Holmes[1], Nerys Huffman[1], Daniel R. Hulbert[1], Eric B. Isaacs[1], Clayton A. C. Jackson[1], Logan Jaeger[1], Ian Jenkins[1], Cameron Jennings[1], Paul C. Jerger[1], B. Johnson[1], Aaron M. Jones[1], Michael P. Jura[1], Adour V. Kabakian[1], Raj M. Katti[1], Tyler Keating[1], Joseph Kerckhoff[1], Joseph D. Kern[1], Isaac Khalaf[1], Aditya Kher[1], Jake J. Kim[2], Erich W. Kinder[1], Andrey A. Kiselev[1], William F. Koehl[1], Patrick W. Krantz[1], Thaddeus D. Ladd[1], Pierce G. Laing[1], Sanaaya Lakdawala[1], Nathan J. Lang[1], Robert Lanza[1], Elias Lawson-Fox[1], Dustin Le[1], Kangmu Lee[1], Nathan R. A. Lee[1], Jaime Lerma[2], Mark P. Levendorf[1], Alwina R. Liu[1], Henry Lizarraga[2], Aurelio Lopez[1], Hoa C. Ly[1], Torrey T. Lyons[1], Theodore K. Macioce[1], Matthew M. Mackey[1], John K. Maeda[1], Ryan M. Martin[1], Daniel S. Matic[1], Justine W. Matten[1], Gavin C. Mazur[1], Max S. McCready[1], Olivia Means[1], Kevin E. Millner[1], Ivan Milosavljevic[1], Matthew Morris[1], Susan L. Morton[1], Samuel Mumford[1], Bryce D. Murley[1], Robert G. Nagele[1], Taro A. Naoi[1], Cameron R. Nelson[1], Georgia A. Newman[1], David B. Nguyen[1], Tina Niknejad[1], Rebecca N. Nishide[1], Liam C. O'Brien[1], Colin B. E. O'Keefe[1], Riley P. O'Neil[1], Andrew E. Oriani[1], Anthony F. Ortiz[1], John J. Ottusch[1], Andrew Pan[1], Pamela R. Patterson[1], Uttam Paudel[1], Julius C. Perez[1], Christi A. Peterson[1], Vu T. Phan[1], Nickolas H. Pilgram[1], Clifford E. Plesha[1], Winston Pouse[1], Eric M. Prophet[1], Daniel R. Queen[1], Nicholas Quirk[1], Kate Raach[1], Matthew T. Rakher[1], Matthew D. Reed[1], Brandon D. Reynolds[1], Luke D. Robertson[1], Zechariah Rogers[1], Yakov Royter[1], Matthew J. Ruiz[1], Golam Sabbir[1], Roshan Sajjad[1], Christopher D. Sanborn[1], Rachel H. Sarmiento[1], Christian J. Schnaible[1], Cole Scott[1], Nicholas M. Sebastiani[1], Eric M. Segall[2], Alen Senanian[1], Adalberto Sicairos[1], Shariq Siddiqui[2], Kartik Singh[1], Aaron Smith[1], Daniel E. Smith[1], Robert S. Smith[1], Sarah F. Sontag[2], Emilio A. Sovero[1], Kevin C. Staley[1], Andrea Su[1], June Suh[1], Bo Sun[1], Danny Sun[1], Christopher M. Swank[1], Noah Swimmer[1], Mariano J. Taboada[1], Bryan J. Thomas[1], Yessica Torres[1], Jeremy W. Touve[1], Alan Tran[1], Ivan Tran[1], Chantang Tsen[1], Skylar Turner[1], Miguel Valencia[1], Irma Valles[1], James R. van Meter[1], Nicholas D. VanRensselaer[2], Franklin Vartanian[1], Daniel Volya[1], Zachary J. Vrba[1], Phuong Hong Vu[1], Annette L. Wagner[1], John Wallner[1], Michael P. Walsh[1], Shuoqin Wang[1], Tong Wang[1], Daniel R. Ward[1], Aaron J. Weinstein[1], Terry B. Welch[1], Thomas V. Westrick[1], Evan T. White[1], Randall M. White[1], Samuel J. Whiteley[1], Gananath Wijeratne[1], Parker Williams[1], Jack T. Wilson[1], Courtney P. Wilt[1], Deborah E. Winklea[1], Onnik Yaglioglu[1], Daniel Yap[1], Clifford S. YoungSciortino[1], Daniel Zehnder[1], Andrew Ziegler[1]

[1]HRL Laboratories, LLC
[2]The Boeing Company

REFERENCES

[1] Reed, M. D. *et al.* Reduced sensitivity to charge noise in semiconductor spin qubits via symmetric operation. *Phys. Rev. Lett.* **116**, 110402 (2016).

[2] DiCarlo, L. *et al.* Differential charge sensing and charge delocalization in a tunable double quantum dot. *Phys. Rev. Lett.* **92**, 226801 (2004).

[3] Blumoff, J. Z. *et al.* Fast and high-fidelity state preparation and measurement in triple-quantum-dot spin qubits. *PRX Quantum* **3**, 010352 (2022).

[4] Fong, B. H. & Wandzura, S. M. Universal quantum computation and leakage reduction in the 3-qubit decoherence free subsystem. *Quantum Inf. Comput.* **11**, 1003–1018 (2011).

[5] Weinstein, A. J. *et al.* Universal logic with encoded spin qubits in silicon. *Nature* **615**, 817–822 (2023).

[6] Reichardt, B. W. *et al.* Fault-tolerant quantum computation with a neutral atom processor. arXiv:2411.11822.

[7] Hukushima, K. & Nemoto, K. Exchange Monte Carlo method and application to spin glass simulations. *J. Phys. Soc. Jpn.* **65**, 1604–1608 (1996).

[8] Khaneja, N., Reiss, T., Kehlet, C., Schulte-Herbrüggen, T. & Glaser, S. Optimal control of coupled spin dynamics: Design of NMR pulse sequences by gradient ascent algorithms. *J. Magn. Reson.* **172**, 296–305 (2005).

[9] Rao, A. S. *et al.* Modular autonomous virtualization system for two-dimensional semiconductor quantum dot arrays. *Phys. Rev. X* **15**, 021034 (2025).

[10] Samaha, P. *et al.* Automatic charge state tuning of 300 mm FDSOI quantum dots using neural network segmentation of charge stability diagram. arXiv:2604.13662.

[11] Taylor, J. R. & Das Sarma, S. Neural network based deep learning analysis of semiconductor quantum dot qubits for automated control. *Phys. Rev. B* **111**, 035301 (2025).

[12] Kalantre, S. S. *et al.* Machine learning techniques for state recognition and auto-tuning in quantum dots. *npj Quantum Inf.* **5** (2019).

[13] Moon, H. *et al.* Machine learning enables completely automatic tuning of a quantum device faster than human experts. *Nat. Commun.* **11**, 4161 (2020).

[14] Zwolak, J. P. *et al.* Autotuning of double-dot devices in situ with machine learning. *Phys. Rev. Appl.* **13**, 034075 (2020).

[15] Darulová, J., Troyer, M. & Cassidy, M. C. Evaluation of synthetic and experimental training data in supervised machine learning applied to charge-state detection of quantum dots. *Mach. Learn.: Sci. Technol.* **2**, 045023 (2021).

[16] Durrer, R. *et al.* Automated tuning of double quantum dots into specific charge states using neural networks. *Phys. Rev. Appl.* **13**, 054019 (2020).

[17] Zwolak, J. P. *et al.* Data needs and challenges for quantum dot devices automation. *npj Quantum Inf.* **10** (2024).

[18] Paquelet Wuetz, B. *et al.* Multiplexed quantum transport using commercial off-the-shelf CMOS at sub-kelvin temperatures. *npj Quantum Inf.* **6**, 43 (2020).

[19] Pauka, S. *et al.* Characterizing quantum devices at scale with custom cryo-CMOS. *Phys. Rev. Appl.* **13**, 054072 (2020).

[20] Eastoe, J. *et al.* Method for efficient large-scale cryogenic characterization of CMOS technologies. *IEEE Trans. Instrum. Meas.* **74**, 1–10 (2025).

[21] Thomas, E. J. *et al.* Rapid cryogenic characterization of 1,024 integrated silicon quantum dot devices. *Nat. Electron.* **8**, 75–83 (2025).

[22] Tosato, A. *et al.* A crossbar chip for benchmarking semiconductor spin qubits. *Nat. Electron.* **9**, 324–333 (2026).

[23] Wolfe, M. A. *et al.* On-chip cryogenic multiplexing of Si/SiGe quantum devices. arXiv:2410.13721.

[24] Puddy, R. K. *et al.* Multiplexed charge-locking device for large arrays of quantum devices. *Appl. Phys. Lett.* **107**, 143501 (2015).

[25] Chen, E. H. *et al.* Detuning axis pulsed spectroscopy of valley-orbital states in Si/SiGe quantum dots. *Phys. Rev. Appl.* **15**, 044033 (2021).

[26] Jones, A. *et al.* Spin-blockade spectroscopy of Si/Si-Ge quantum dots. *Phys. Rev. Appl.* **12**, 014026 (2019).

[27] Hollmann, A. *et al.* Large, tunable valley splitting and single-spin relaxation mechanisms in a Si/Si_xGe_{1-x} quantum dot. *Phys. Rev. Appl.* **13**, 034068 (2020).

[28] Marcks, J. C. *et al.* Valley splitting correlations across a silicon quantum well containing germanium. *Nat. Commun.* **16**, 11381 (2025).

[29] Lodari, M. *et al.* Low disorder and high valley splitting in silicon. *npj Quantum Inf.* **10**, 26 (2024).

[30] Volmer, M. *et al.* Mapping of valley splitting by conveyor-mode spin-coherent electron shuttling. *npj Quantum Inf.* **10**, 61 (2024).

[31] Wuetz, B. P. *et al.* Effect of quantum Hall edge strips on valley splitting in silicon quantum wells. *Phys. Rev. Lett.* **125**, 186801 (2020).

[32] Stehouwer, L. E. A. *et al.* Engineering Ge profiles in Si/SiGe heterostructures for increased valley splitting. *Nano Letters* **25**, 12892–12898 (2025).

[33] Ando, T., Fowler, A. B. & Stern, F. Electronic properties of two-dimensional systems. *Rev. Mod. Phys.* **54**, 437 (1982).

[34] Andrews, R. W. *et al.* Quantifying error and leakage in an encoded Si/SiGe triple-dot qubit. *Nat. Nanotechnol.* **14**, 747–750 (2019).

[35] Kerckhoff, J. *et al.* Magnetic gradient fluctuations from quadrupolar ^{73}Ge in Si/SiGe exchange-only qubits. *PRX Quantum* **2**, 010347 (2021).

[36] Voss, R. F. Efficient incremental simulation of "1/f" noises. *Fractals* **30**, 2250020 (2022).

[37] Lin, H., Gubernatis, J., Gould, H. & Tobochnik, J. Exact diagonalization methods for quantum systems. *Comput. Phys.* **7**, 400–407 (1993).

[38] Gray, J. quimb: A python package for quantum information and many-body calculations. *J. Open Source Softw.* **3**, 819 (2018).

[39] Gray, J. & Kourtis, S. Hyper-optimized tensor network contraction. *Quantum* **5**, 410 (2021).

[40] Gray, J. Autoray: a python automatic-array library (2026). URL `https://github.com/jcmgray/autoray`.

[41] Okuta, R., Unno, Y., Nishino, D., Hido, S. & Loomis, C. *Cupy: A numpy-compatible library for NVIDIA GPU calculations*, NIPS 2017 Workshop on Machine Learning Systems (2017).

[42] Blume-Kohout, R. & Young, K. Estimating detector error models from syndrome data. arXiv:2504.14643.

[43] Nielsen, E. *et al.* Probing quantum processor performance with pyGSTi. *Quantum Sci. Technol.* **5**, 044002 (2020). For DEM analysis, we use `pygsti.extras.sparsedems`.

[44] Higgott, O. & Gidney, C. Sparse Blossom: correcting a million errors per core second with minimum-weight matching. *Quantum* **9**, 1600 (2025).

[45] Chen, Z. *et al.* Exponential suppression of bit or phase errors with cyclic error correction. *Nature* **595**, 383–387 (2021).

[46] Sun, B. *et al.* Full-permutation dynamical decoupling in triple-quantum-dot spin qubits. *PRX Quantum* **5**, 020356 (2024).
