# Supplementary material for: A digitally controlled silicon quantum processing unit
Source: Nature. 2026 Jul 29;655(8125):1154–9. doi: 10.1038/s41586-026-10754-7 (PMC13421337; doi:10.1038/s41586-026-10754-7)
Supplement: Supplementary file 2 — Peer Review File [file 41586_2026_10754_MOESM2_ESM.pdf]

nature portfolio

Peer Review File

A digitally controlled silicon quantum processing unit

Corresponding Authors: Dr Thaddeus Ladd, Dr Matthew Reed, Dr Jacob Blumoff

Any redactions in this file are there to maintain patient confidentiality, the confidentiality of unpublished data, or to remove third-party material.

This file contains all reviewer reports in order by version, followed by all author rebuttals in order by version.

Version 0:

Reviewer comments:

Referee #1

(Remarks to the Author)
Indeed, the manuscript “A digitally controlled silicon quantum computer processing unit” by members of the HRL Quantum Team and collaborators marks a significant milestone in the technological maturity of semiconductor spin qubits. My congratulations to all the authors for this remarkable progress! All key building blocks of a quantum computer such as the cryogenic controller, a highly isotopically purified Si/SiGe quantum chip with environmental control, heat isolation, automated tuning and qubit characterization, as well as high-performance qubit operations and multi-qubit experiments explained by simulations are all working together and are integrated into a single platform. The result is the operation of a quantum processing unit that outperforms other semiconductor spin qubit platforms by far. The work demonstrates that encoded spin qubits here exchange-only qubits, despite their complexity in pulse sequences and susceptibility to leakage states, can be effectively combined with a cryogenic silicon control chip, provided that pulse control is sufficiently precise and reset-if-leaked protocols are implemented. Compared to other platforms, the total qubit count is still relatively low; however, the processor is fast, exhibits low sensitivity to quantum chip noise, and remains compact due to the integrated cryogenic control.

The manuscript is well written, and the authors provide more than 60 pages of well-organized supplementary information (SI) covering all system components. However, the claim that valley splitting is no longer a limiting factor (line 238) is not clearly explained or convincingly demonstrated within the manuscript. Furthermore, the assertion that the system design provides a practical foundation for utility-scale quantum computing (line 249) would benefit from additional supporting arguments.
I recommend publication in Nature, provided that the authors address these points by supplying the missing information to support their claims and clarifying the relevant sections of the text.

1) The authors state (line 261) that “the scale and cost … position semiconductor spin qubits as a leading technology for realizing commercially relevant quantum computing.” Please provide a more detailed discussion of the associated costs of your processor components to substantiate this claim.

2) The authors state (line 237) that “conventional concerns such as … valley splitting … are no longer limiting,” attributing this to a “quantum well which is engineered to increase valley splitting” (line 50), and further describing an “internally conceived and optimized quantum well design” (SI, p. 31). They claim that the valley splitting (VS) is “comparable to or larger than single-electron orbital excitations, which are usually around 1–2 meV” (SI, p. 31).
If confirmed, this would represent a significant advance for electron spin qubits in Si/SiGe. However, caution is warranted, as orbital and valley splittings can be difficult to distinguish experimentally, and several established methods lack the resolution to reliably detect small valley splittings <10 µeV, which have previously been reported in Si/SiGe systems.
A) The authors argue that VS can be measured using DAPS (Fig. S29) and that orbital and valley splittings can be distinguished by tuning a neighboring exchange gate, since “the orbital energy will change more strongly than the VS” (Fig. S29 caption). However, the opposite behavior can occur: VS may vary significantly when the quantum dot is displaced or its shape is modified. This effect has been reported in multiple prior studies (not cited in SI). Furthermore, Fig. S29 raises several questions: Why is only limited data shown? Why is the exchange gate bias “arbitrary”? What degree of dot displacement or deformation is expected under these conditions?
B) The authors further argue that VS must be large because the qubit initialization and measurement window is limited by

the two-electron orbital energy (Fig. S23), reported to lie between 120 µeV and 480 µeV (based on a small subset of data points from nine 54-dot devices). However, it is possible that this limitation instead reflects valley splitting that is misidentified as orbital excitation energy.
C) The use of the quantum Hall effect to extract VS (Fig. S30) involves operation at large out-of-plane magnetic fields. It has been shown in prior work that such measurements can overestimate VS. Additional clarification and justification of the analysis in Fig. S30 are needed.
D) At first glance, the high qubit yield (Fig. S28b) might suggest large VS across the whole wafer. However, the yield metric is based on the operability of gate electrodes (Fig. S28a), and only the number of "anticipated operable qubits" is reported, which does not directly provide evidence for large VS.
In my view, the current evidence is insufficient to support the strong claim that the valley splitting limitation in Si/SiGe has been overcome. I would expect, for example, a statistical analysis of VS across devices, a structural characterization of the Si/SiGe heterostructure, and a comparison with existing SiGe alloy/strain theoretical models of valley splitting. I therefore request that the authors either provide more compelling evidence or revise the strength of their statements regarding VS in the main text.
Importantly, the work represents a significant milestone even without this strong claim regarding valley splitting.

3) Regarding the scalability of the system design (line 249): Please clearly define what is meant by "utility-scale quantum computing". If this refers to a system comprising on the order of millions of electron-spin (EO) qubits, I have significant concerns about the scalability of the presented approach:
A) The reported power dissipation of the controller chip is approximately 3 W. Do the authors anticipate the need for megawatt-scale cooling power at 4 K, or do they foresee opportunities to reduce the power consumption by orders of magnitude?
B) While the cryogenic controller and superconducting interconnects may alleviate the room-temperature bottleneck, the land grid array of the quantum chip itself appears to become a limiting factor at the scale of millions of qubits. Can the authors clarify how this constraint is addressed, and whether a more scalable interconnect or packaging strategy is envisioned?
C) The presented qudot architecture appears to be effectively 1D (3 × 18). Do the authors envision scaling this approach to a truly two-dimensional architecture (e.g., a square lattice with ~1E6 qubits and corresponding on-chip wiring fan-out), or is scalability primarily achieved by increasing the number of such sectors in 1D?
Overall, I encourage the authors to provide a more concrete and quantitative discussion of the scaling roadmap and associated engineering challenges.

3) While the device is reported to host up to 18 EO qubits, Fig. 3a presents performance data for only 7 qubits (formed towards upper left of chip). Does this indicate that the yield of high-performance qubits (as opposed to merely operable gate electrodes, cf. Fig. S28) is relatively limited? Please provide quantitative statistics on qubit performance and yield across the full device.

4) Line 55: How is the charge noise improved to previous SLEDGE technology?

5) Line 83: Please explain how "circuit designs are optimized at the transistor level for performance at 4 K".

6) Fig. 1: The device requires 35 static voltage biases. For which specific gates are these biases needed? Why are they not supplied by the cryogenic controller? Do the authors foresee that future iterations of the controller will be capable of providing these static voltages?

7) Fig. 2c: I understand that DACs are ramped for tune-up and SPAM. Are DACs voltages constant during exchange operations? Why does P/M/Z required 4 voltage levels and X/Y/T only two or does that depend on the sector? Where are the tunable capacitors of Fig. S4 in Fig. 2c?

8) Fig. 3a: How long are the single qubit gates (4 pulses) and two qubit gates (37) pulses in lab time? (some values would be helpful in the main text) Are all "timesteps" equally long? How long are initialization, readout and RIL?

9) Line 148: Is the scaling factor meaningful despite the "unexplained fluctuations" (Are these fluctuations related to the correlated spikes in detector 1-3?, maybe directly refer to the SI). The LER of [3,1,1] in Fig. 4d is lower than the one of [5,1,1] in Fig. 4c.

10) line 316: What is meant by "alloy composition range"? Is there a composition gradient in the barrier or a composition super-lattice or just composition variations from wafer to wafer quoted here?

11) Line 317: What is the degree of isotopically reduction of 29Si and 73Ge?

12) Line 318: What is the quantum well thickness? What is the thickness of the upper SiGe barrier?

13) Line 318: The quantum well heterostructure, at least the nominal Ge concentration profile, should be specified.

14) Line 325: Specify the Ar process or give references. What is the lateral distance of Ar to quantum dots? Does the Ar process replace a mesa?

15) Fig. 4c appears before Fig. 4b in the text. Consider swap.

16) Extended Data Fig. 1: Please show a photograph of the hardware used in the experiment including 4 K TIA.

17) SI Fig. S20: Are all EO operated in (1,1,1) or do you use other dot fillings sometimes?

18) SI Fig. S23: Why are only 14 values of two-electron orbitals shown here, although nine 54-dot devices have been measured? Are these measurements done at specific positions on the chip only?

19) SI Fig. S24: Please mark the position of the 7 EO qubits and reservoirs in the presented bias constellation. Please refer to the “alternative readout configuration” (SI page 48), which seems to be used here (or refer to Fig. S44).

20) It is not clear what factors limited the number of EO qubits utilized on the chip. Is this primarily a matter of measurement time, qubit yield, controller limitations, or other constraints? Please clarify the dominant bottlenecks and whether higher qubit counts have been used but are not shown.

Referee #2

(Remarks to the Author)

This manuscript summarizes an 18 qubit chip with improved performance over previous state of the art spin qubit chips operated in the exchange only encoding. Additionally, the authors control the qubit with a cryogenic CMOS based control chip. They perform characterization algorithms of their device.

The paper is of high quality and should be accepted.

Two requested revisions:

1) The paper highlights an advancement of spin qubit performance in several places. Excellent. However, the paper largely ignores the performance of spin qubits compared to other qubit modalities. Are spin qubits now competitive with leading superconducting, ion trap, and neutral atom quantum systems. I believe that the authors would say "yes." Including a cursory comparison would amplify their messages.

2) The authors do a thorough job of discussing their error pareto throughout. They mention in the Qubit Chip Methods section that they are using superconducting on chip wires. Context here is requested. Does the prescence (or absence) contribute to the qubit performance at this scale or is it necessary only for larger systems. This is largely the only qubit feature that is not discussed in more detail.

No obvious typos or grammar issues are present. This reviewer notes the term LRU is used but not defined in line 157. It is only defined in the text of Figure 4.

Referee #3

(Remarks to the Author)
The manuscript reports a significant advance: an integrated silicon exchange-only quantum processing unit combining a 4 kelvin cryogenic CMOS controller and a high-density superconducting interconnect interfacing with a silicon qubit chip hosting one of the largest quantum dot arrays reported to date. The work addresses a central bottleneck in scaling semiconductor spin qubits, namely the generation and delivery of many control signals. The demonstrated one-qubit and CNOT performance, distance-5 repetition-code experiment, and [[4,2,2]] quantum error-detection experiment make the paper of interest to the quantum information, cryogenic electronics, and quantum hardware communities. The development of cryo-CMOS qubit controllers is rapidly gaining importance, especially given the stringent power constraints associated with large-scale quantum control systems and the broader goal of enabling commercially viable quantum computing.

The conclusions are original due to the unique integration of key components. Several of these components build on important precedents, which the manuscript mostly cites appropriately. Relevant literature that helps put this work in context includes, for instance, the demonstrations of single-qubit fidelities of 99.99% across five qubits in Si/SiGe architectures (arXiv:2507.11918). While the present work extends the number of spin qubits to seven, six-qubit quantum circuits on spin-based processors have been reported (PRX Quantum 7, 010308 (2026)). There is also relevant literature on some of the methodologies employed, including automated qubit tuning (e.g. Physical Review X 15, 021034 (2025), Nature Electronics 9, 304 (2026)), and the use of superconducting ribbon cables (e.g. arXiv:2306.13574).

The paper is technically sound, and the data analysis is clear. One point to note is that the caption of Fig. 3 suggests that a different device was used to obtain the results shown in that figure. It would be interesting to know how many devices were measured. This is relevant given the discussion about commercial relevance in the conclusion. In the abstract, the authors state that the 54-quantum-dot architecture could support up to 18 exchange-only qubits. While the number of quantum dots in this chip is in principle sufficient to encode 18 exchange-only qubits, the demonstrated algorithms involve no more than seven qubits in the largest implementation. It would be helpful to clarify whether these demonstrations always used the same seven qubits, and how representative these qubits are of the full array.

The outstanding feature of the work is the system-level integration. The manuscript goes beyond demonstrating qubit array

performances and instead shows a plausible architecture for scaled control.

A few questions and comments:

- As shown in Fig. 1, the device includes only a single row of dot charge sensors located at the bottom of the array. In the Supplementary Information, the authors describe an “alternative readout configuration” in which dots within the qubit array are repurposed to enable readout of a smaller subset of qubits with improved charge sensitivity. It is therefore unclear how high-fidelity readout would be achieved when the array is fully populated.

- Fig. 4 describes a large number of operations required for a single instance of the 200-round experiment, including over 1,800 initialisations, 800 measurements, and 330,000 exchange operations. What is the laboratory time required for a single instance? This information would allow useful comparison with other platforms and with other spin-qubit processors.

- In the closing statements, the authors claim that conventional concerns such as charge noise, valley splitting, and leakage out of the computational space are no longer limiting. This statement may be questioned. The shot rejection in the [[4,2,2]] post-selected analysis seems high. Also, work on valley-splitting engineering suggests that improvements can come at the cost of reduced mobility. Providing the range of mobilities achieved would help clarify the extent to which the hardware advances presented in this paper solve a long-standing problem in silicon qubit devices.

- Finally, how would this wiring strategy scale, given the size constraints associated with indium bumps/bond pads?

Minor points:

- In Fig. 3a, the legend and vertical reference lines do not appear to match the mean error values reported in the figure caption?

- In Figs. 3b-c, the reported number of observed oscillations and the median T_2^* values appear to imply characteristic frequencies that can differ from the exchange-frequency values quoted in the Supplementary Materials.

- In Fig. 4c, light-shaded triangular markers are shown alongside solid data points. Their meaning could be explained in the figure caption or the main text, including why no analogous square markers are shown. Similarly, the meaning of the error bars associated with fidelity and throughput in Fig. 4e could be specified in the caption.

- For the temperature estimates shown in Fig. S14, it would be helpful to provide fits and gate lever arms used to extract the electron temperatures.

Open Access This Peer Review File is licensed under a Creative Commons Attribution 4.0 International License, which permits use, sharing, adaptation, distribution and reproduction in any medium or format, as long as you give appropriate credit to the original author(s) and the source, provide a link to the Creative Commons license, and indicate if changes were made.

In cases where reviewers are anonymous, credit should be given to 'Anonymous Referee' and the source.
The images or other third party material in this Peer Review File are included in the article's Creative Commons license, unless indicated otherwise in a credit line to the material. If material is not included in the article's Creative Commons license and your intended use is not permitted by statutory regulation or exceeds the permitted use, you will need to obtain permission directly from the copyright holder.
To view a copy of this license, visit https://creativecommons.org/licenses/by/4.0/

A digitally controlled silicon quantum processing unit: referee response

Members of the HRL Quantum Team and Collaborators

We thank the referees and editors for their gracious and thoughtful attention to our work. Here are item-by-item responses to each of the questions and issues raised.

Response to Referee #1

We open our response to Referee #1 with an acknowledgment of the evident care and expertise of their review. The questions raised reflect close engagement with both the manuscript and the broader literature. Where the referee has identified overreach, we have softened the language, most notably in assertions about uniformly large valley splitting that is not directly supported by available data. There, we have instead substantiated our claim that valley splitting is not an impediment to performance with more circumstantial evidence like the lack of multiple frequencies in exchange oscillations and the reliability of finding spin-to-charge. On several technical inquiries, particularly those relating to our epitaxial stack and fabrication process and to broader scaling-roadmap questions, we regret that we cannot be as forthcoming as the referee might prefer. As has become common practice for commercial entities engaging with the scientific community, certain technical details must remain undisclosed. Other questions about demonstrations or quantum architecture at larger scale will be best addressed by forthcoming work with differentiated scientific focus. Where these constraints apply, we have noted them explicitly in the individual responses below and, where possible, directed the referee to data already available in the manuscript and Supplementary Information that bears on each question.

Note also, due to a clerical issue, we renumbered two of Ref. 1's comments as "3.1" and "3.2" below.

> *1) The authors state (line 261) that "the scale and cost … position semiconductor spin qubits as a leading technology for realizing commercially relevant quantum computing." Please provide a more detailed discussion of the associated costs of your processor components to substantiate this claim.*

Our claim rests on the structural cost economics of wafer-scale semiconductor manufacturing

rather than on a quantitative cost projection. At the qubit counts required for commercial relevance ($\sim 10^5$ to 10^6 physical qubits [Beverland et al., arXiv:2211.07629; Gidney & Ekerå, *Quantum* 2021]), per-system cost will be dominated by manufacturing and control overhead replicated per qubit. As shown in the history of conventional computing, wafer-scale lithographic manufacturing and integration is the most economical method available for producing the millions of identical features needed for this scale. Architectures whose per-qubit overhead inherits this manufacturing path will diverge structurally in cost from those that rely on bespoke techniques. It has been long established that semiconductor spin qubits can be produced on the same industrial fabrication lines as conventional integrated circuits [Zwerver et al. *Nature* 2022, Ref. [11]; Vandersypen et al. *npjQI* 2017; Boter et al. *Phys. Rev. Appl.* 2022]. The QPU demonstrated here further shows that the control and interconnection hardware required to control the spin qubits can also be manufactured at wafer scale and integrated in a way compatible with conventional cryogenic infrastructure. We believe the references already cited in the main text adequately ground the structural argument, and respectfully decline to add quantitative cost figures, which would depend on yield, volume, and roadmap assumptions that the present prototype cannot substantiate.

2) The authors state (line 237) that "conventional concerns such as … valley splitting … are no longer limiting," attributing this to a "quantum well which is engineered to increase valley splitting" (line 50), and further describing an "internally conceived and optimized quantum well design" (SI, p. 31). They claim that the valley splitting (VS) is "comparable to or larger than single-electron orbital excitations, which are usually around 1–2 meV" (SI, p. 31).

If confirmed, this would represent a significant advance for electron spin qubits in Si/SiGe. However, caution is warranted, as orbital and valley splittings can be difficult to distinguish experimentally, and several established methods lack the resolution to reliably detect small valley splittings <10 μeV, which have previously been reported in Si/SiGe systems.

A) The authors argue that VS can be measured using DAPS (Fig. S29) and that orbital and valley splittings can be distinguished by tuning a neighboring exchange gate, since "the orbital energy will change more strongly than the VS" (Fig. S29 caption). However, the opposite behavior can occur: VS may vary significantly when the quantum dot is displaced or its shape is modified. This effect has been reported in multiple prior studies (not cited in SI). Furthermore, Fig. S29 raises several questions: Why is only limited data shown? Why is the exchange gate bias "arbitrary"? What degree of dot displacement or deformation is expected under these conditions?

B) The authors further argue that VS must be large because the qubit initialization and measurement window is limited by the two-electron orbital energy (Fig. S23), reported to lie between 120 μeV and 480 μeV (based on a small subset of data points from nine 54-dot

devices). However, it is possible that this limitation instead reflects valley splitting that is misidentified as orbital excitation energy.

C) The use of the quantum Hall effect to extract VS (Fig. S30) involves operation at large out-of-plane magnetic fields. It has been shown in prior work that such measurements can overestimate VS. Additional clarification and justification of the analysis in Fig. S30 are needed.

D) At first glance, the high qubit yield (Fig. S28b) might suggest large VS across the whole wafer. However, the yield metric is based on the operability of gate electrodes (Fig. S28a), and only the number of "anticipated operable qubits" is reported, which does not directly provide evidence for large VS.

In my view, the current evidence is insufficient to support the strong claim that the valley splitting limitation in Si/SiGe has been overcome. I would expect, for example, a statistical analysis of VS across devices, a structural characterization of the Si/SiGe heterostructure, and a comparison with existing SiGe alloy/strain theoretical models of valley splitting. I therefore request that the authors either provide more compelling evidence or revise the strength of their statements regarding VS in the main text. Importantly, the work represents a significant milestone even without this strong claim regarding valley splitting.

We appreciate the referee's thoughtful consideration regarding these points. There are two separate but related claims in the original manuscript: first, that valley splitting does not operationally limit performance in these devices (the primary statement in the main text), and second, that our process yields meV-scale valley splittings across wafers (which the VS section in the SI focuses on). The first claim relates to the question of qubit yield, as the referee points out in their point (D): how often do we encounter qubits whose behavior or performance shows signs of being limited by a small valley splitting? This typically manifests as small singlet-triplet splitting at SPAM sites [Jones et al., *Phys. Rev. Appl.* 2019] or as multiple exchange frequencies in fingerprints due to qubit population in excited states [Reed et al., *PRL* 2016, Ref. [38]]. These signatures are absent in devices with our quantum well design, as illustrated by the distribution of initialization and measurement windows in Fig. S23 and the fingerprint sampling in Fig. S26. The lack of valley-related obstacles in device tuneup and performance is our key empirical observation. We have rewritten the SI to emphasize these points.

In terms of the DAPS and quantum Hall measurements indicating meV-scale valley splittings in our devices, we agree that objections could be raised to each individual data point presented in the SI. To address each question in turn, regarding point (A), we agree that VS can also change due to quantum dot displacement and deformation, and we have added several references to dot tuning and shuttling experiments showing this. However, when we perform these experiments, we consistently observe that the lowest orbital-attributed excited state is reduced with increasing

aggressor gate voltage (which reduces the confinement) while the other lowest valley-attributed excited state generally changes little or not at all. This *direction* of change agrees with both intuition and simulation expectations for orbital deformation. While valley splitting can also change due to dot displacement in a quantum well with disorder, whether it increases or decreases would be expected to vary randomly from dot to dot. Regarding the limited DAPS data, we have chosen representative measurements from our devices to illustrate the magnitude of splittings we typically observe. Single-electron DAPS measurements on wafers using our quantum well process consistently show only features starting around the meV scale, requiring additional bias experiments and measurements to try to resolve the nature of the states. Detailed sweeps and analysis for many dots was not available at the time of preparation of this manuscript.

Regarding point (B), we agree that the (two-electron) splittings of each initialization window presented in Fig. S23 could in principle also be limited by valley splitting. We have revised the SI discussion to reflect this. However, we note that we typically observe consistent directional dependence on bias for these energies versus aggressor voltage, similar to the DAPS measurements discussed, which are indicative of orbital reshaping of the excited state energy. Those observations together with the single-electron DAPS data lead us to believe the singlet-triplet splittings are generally orbital-limited. We also note that this is strong evidence against the possibility that the valley splitting is simply too small ($< 10\ \mu$eV) to be resolved in DAPS or other spectroscopy methods.

With respect to the quantum Hall measurements, we acknowledge the referee's point (C) that the extracted VS from those measurements can differ from a quantum dot valley splitting due to a number of reasons. We see the quantum Hall data as supporting evidence for large valley splittings in the quantum well, as both the extracted splittings, and the temperature to which we are able to observe them, are significantly higher than reported before in the literature using this technique. We have revised the discussion of the quantum Hall results in the SI to reflect both these points.

We also completely agree with the referee's general point that a full analysis of valley splitting would ideally entail more exhaustive statistical studies, detailed structural characterization, and close comparison with microscopic models. Unfortunately, the proprietary

nature of that information prevents a complete discussion of those aspects of our work. Instead, we have revised the SI to address the individual caveats raised by the referee, emphasizing the consistency of each type of measurement with the presence of elevated valley splitting across the device. We have also revised the main text to read "...nor do we see signs of valley splitting inhibiting performance," reflecting our empirical observations.

> *3.1) Regarding the scalability of the system design (line 249): Please clearly define what is meant by "utility-scale quantum computing". If this refers to a system comprising on the order of millions of electron-spin (EO) qubits, I have significant concerns about the scalability of the presented approach.*

"Utility-scale" does not have a clear or concise definition, as the "scale" changes with advances in fault-tolerance and the "utility" changes with discoveries in algorithms. Therefore it is only a rough estimate, often deemed of order 10^3 logical qubits housed in order of 10^6 physical qubits, enabling computations that are (1) beyond the reach of classical supercomputers running for about a month and (2) useful for some application *beyond* benchmarking quantum computers. Even those rough estimates would be hotly debated if declared in the text of a publication like this, and we certainly have introduced nothing to support them. As such this question is best left out of scope of the manuscript. Many interested readers will have in mind a range of what "utility" means to them, which will be sufficient for the forward-looking, qualitative discussion of this part of the manuscript.

> *A) The reported power dissipation of the controller chip is approximately 3 W. Do the authors anticipate the need for megawatt-scale cooling power at 4 K, or do they foresee opportunities to reduce the power consumption by orders of magnitude?*
>
> *B) While the cryogenic controller and superconducting interconnects may alleviate the room-temperature bottleneck, the land grid array of the quantum chip itself appears to become a limiting factor at the scale of millions of qubits. Can the authors clarify how this constraint is addressed, and whether a more scalable interconnect or packaging strategy is envisioned?*
>
> *C) The presented qudot architecture appears to be effectively 1D (3 × 18). Do the authors envision scaling this approach to a truly two-dimensional architecture (e.g., a square lattice with 1E6 qubits and corresponding on-chip wiring fan-out), or is scalability primarily achieved by increasing the number of such sectors in 1D?*
>
> *Overall, I encourage the authors to provide a more concrete and quantitative discussion of the scaling roadmap and associated engineering challenges.*

These questions are the big ones: indeed, we acknowledged them in our concluding paragraph saying, "Necessary advances include a manufacturable interconnect and qubit back-end routing solution, controllers with even lower power consumption per qubit, and improved device

uniformity to reduce tune-up overhead. A system-level quantum architecture tailored to the connectivity, noise environment, and other properties of EO qubits must also be elaborated." We agree with Referee #1 that significant innovations in per-qubit control power, interconnect density, and quantum architecture are necessary and it will be incumbent on us (and the community at large) to provide solutions to these problems. A complete elaboration of our intended roadmap is beyond the scope of this manuscript, however, which focuses on demonstrating the central integrated elements at sufficient scale and performance to invite that broader effort.

> *3.2) While the device is reported to host up to 18 EO qubits, Fig. 3a presents performance data for only 7 qubits (formed towards upper left of chip). Does this indicate that the yield of high-performance qubits (as opposed to merely operable gate electrodes, cf. Fig. S28) is relatively limited? Please provide quantitative statistics on qubit performance and yield across the full device.*

As mentioned in our response to question #20 below, the selected multiqubit circuits reflect the intended focus of this manuscript to be on system integration. Demonstrations utilizing a larger fraction of QPU resources are forthcoming (principally enabled not by improvements in yield but simply more measurement time), but the scientific narrative and focus of those works will be different. Publication-quality characterization data beyond what was used to parameterize and model the circuits demonstrated here are not currently available, but will be included in future work.

> *4) Line 55: How is the charge noise improved to previous SLEDGE technology?*

In brief, as we indicate in the text, using a "proprietary" gate-stack different from SLEDGE. As is common practice with commercial entities engaging with the scientific community, we are intentionally not disclosing some of the details of our qubit fabrication process. Citations 10-12 in the main text, all recent publications in *Nature*, provide more examples of demonstrations using semiconductor quantum dots produced with undisclosed processes.

> *5) Line 83: Please explain how "circuit designs are optimized at the transistor level for performance at 4 K".*

The cryo-controller is a full-custom mixed-signal design that affords the ability to optimize designs at the transistor level. The "transistor level" descriptor in the text has been expanded to make reference to design practices and circuit techniques. Namely, we added the phrase "using a combination of design practices and circuit techniques specifically developed for cryogenic

operation". A detailed description of the specific circuit design techniques is not an intended focus of this work, but may be included in future, more specialized publications.

6) Fig. 1: The device requires 35 static voltage biases. For which specific gates are these biases needed? Why are they not supplied by the cryogenic controller? Do the authors foresee that future iterations of the controller will be capable of providing these static voltages?

Those 35 signals are voltages for 24 inner field gates (IFG), 8 bath gates (B), 1 supply gate (SG), 1 outer field gate (OFG), and 1 depleting field gate (DFG). Many of these biases are, in practice, held to the same value and could be easily consolidated. They are not presently sourced from the controller for simplicity, but we anticipate revisiting that choice in a future system revision. We thank Ref. 1 for pointing out this deficiency and have added a sentence to the supplementary information (section S6.B) rectifying it.

7) Fig. 2c: I understand that DACs are ramped for tune-up and SPAM. Are DACs voltages constant during exchange operations? Why does P/M/Z required 4 voltage levels and X/Y/T only two or does that depend on the sector? Where are the tunable capacitors of Fig. S4 in Fig. 2c?

Yes, DAC voltages are constant during exchange operations. The P gates require more voltage levels than X/Y gates because they must provide compensating "alignment" pulses to up to three nearby X/Y gates. These alignment pulses ensure that the system remains close to the symmetric operating point [38] to minimize the effect of charge noise. The required amplitude of these alignment pulses is a function of the X gate throw it must compensate, which may be different for each neighbor. Not all P gates have three neighboring exchange gates (some have only two), but we keep the CMOS block fixed for simplicity (and also recycle those blocks for M and Z, again, to minimize design variations). X and Y gates never need more than 2 voltages since they are only ever fully off ("idle") or fully on (setting $J/h \sim$ 100s MHz). The variable capacitors connected by the switch S3 instances in Fig. S4 are not explicitly shown for simplicity but are contained within the red switch network blocks in Fig 2c. They serve to optionally reduce the slew rate of fast pulses and are referenced with the word "filtering" in the caption of Fig 2. It is important to note that neither figure is intended as a literal circuit diagram; rather, both serve as conceptual abstractions designed to illustrate the controller's function rather than its physical implementation.

Regarding this functionality, we originally wrote: "The first two functions are achieved by dynamically changing DAC output voltages and the third with fast, time-modulated, gate-

synchronized switching between fixed DAC voltages." In order to make this more clear, we've changed the end of that sentence to say "switching between DAC voltages that are held constant."

8) Fig. 3a: How long are the single qubit gates (4 pulses) and two qubit gates (37) pulses in lab time? (some values would be helpful in the main text) Are all "timesteps" equally long? How long are initialization, readout and RIL?

We have added information about the pulse-to-pulse cadence (varying between 12 and 28 ns) to the caption of figure 3. We have also noted that within an operation, all "time steps" are equally long, though some opportunities to pulse are intentionally skipped, leading to some effective "doubled" pulse durations. As noted in the text, the RIL gate sequences consist of 30 time steps. We have added additional information to the Supplementary Information (end of section S3.D) about the duration of initialization and measurement operations. We thank the referee for calling our attention to this, as the previous text left the impression that SPAM durations were equivalent to that of Blumoff et al. *PRXQ* 2022, Ref. [35], and this is not the case for the data taken here.

9) Line 148: Is the scaling factor meaningful despite the "unexplained fluctuations" (Are these fluctuations related to the correlated spikes in detector 1-3?, maybe directly refer to the SI). The LER of [3,1,1] in Fig. 4d is lower than the one of [5,1,1] in Fig. 4c.

The scaling factor is still meaningful. The unexplained fluctuations are a performance hit to LER when they occur, as they cause random syndrome outcomes, but they occur in a minority of syndromes. If we were to post-process the [5,1,5] data and the [3,1,3] subsets data to remove these fluctuation events, we expect Λ would improve. We did not perform any data pruning in the main text figures, as that creates misleading measures of quantum circuit performance. The referee is correct that these are the same fluctuations described in the SI section S8.C.2, and we have updated the SI reference on line 151 to point to that specific section.

Concerning the lower LER for [3,1,3] in Fig. 4d, indeed the LER for the [3,1,3] circuit reported in the caption of Fig. 4d is lower than the LER of the [3,1,3]-subset data drawn from the [5,1,5] experiment, shown in Fig. 4b (formerly 4c). This is because the four qubits used in the experiment for Fig. 4d were in a different (superior) calibration state than the 7-qubits used for the [5,1,5] experiment. Simultaneous calibration of additional qubits lowered the average performance of SPAM and two-qubit gates, so the overall LER for $d = 5$ was worse than that of

the independent $d = 3$ circuit, despite the larger code distance. We have added an extra description of this confounding point to the caption for 4d.

10) line 316: What is meant by "alloy composition range"? Is there a composition gradient in the barrier or a composition super-lattice or just composition variations from wafer to wafer quoted here?

Composition variations from wafer to wafer. We changed this line to "wafer-to-wafer alloy compositions ranging from $x = 0.25$ to 0.35" to clarify.

11) Line 317: What is the degree of isotopically reduction of 29Si and 73Ge?

12) Line 318: What is the quantum well thickness? What is the thickness of the upper SiGe barrier?

13) Line 318: The quantum well heterostructure, at least the nominal Ge concentration profile, should be specified.

We are regrettably unable to provide additional technical information about our epitaxy. We are hopeful that our ECDF of T_2^* values in Fig. 3, which originates from residual ^{29}Si and ^{73}Ge nuclei, in conjunction with Kerckhoff et al., *PRXQ* (2021) [Ref. 39] and the references provided in the methods section, will prove an adequate substitute.

14) Line 325: Specify the Ar process or give references. What is the lateral distance of Ar to quantum dots? Does the Ar process replace a mesa?

We have included a reference to a previous publication using these implants and also clarify in the main text that the Ar isolation implant replaces any mesa etch. As discussed in our answer to question #4, we are regrettably unable to provide additional technical information about our proprietary epitaxial stack and manufacturing processes.

15) Fig. 4c appears before Fig. 4b in the text. Consider swap.

We have reordered Figure 4 subpanels as suggested.

16) Extended Data Fig. 1: Please show a photograph of the hardware used in the experiment including 4 K TIA.

We have updated this figure to one that includes the TIAs. We have also added a new extended data figure to more clearly show the integration of the ribbon cable and replaced the original ribbon photo with one depicting the correct generation of part used in this work.

17) SI Fig. S20: Are all EO operated in (1,1,1) or do you use other dot fillings sometimes?

Using more than one electron is a trick sometimes used to mitigate low-lying excited states, which fortunately is not an issue with our devices due in part to epitaxy engineering. All dots used as a part of a DFS qubit in the data presented in this manuscript were tuned to an occupation of 1 electron. We have added a note to the Supplemental Information section S6.B specifying this.

18) SI Fig. S23: Why are only 14 values of two-electron orbitals shown here, although nine 54-dot devices have been measured? Are these measurements done at specific positions on the chip only?

We typically only measure this value at SPAM sites (since that's where the 2e orbital energy is most operationally relevant), of which each device only has six. Further, since the purpose of a given experiment varies, not all devices were fully characterized, leading to the smaller number of 14. However, it is worth clarifying that this data set is comprehensive—values smaller than these were neither observed nor excluded from the published dataset.

19) SI Fig. S24: Please mark the position of the 7 EO qubits and reservoirs in the presented bias constellation. Please refer to the "alternative readout configuration" (SI page 48), which seems to be used here (or refer to Fig. S44).

We have updated Fig S24 to indicate which regions were "flooded forward" and which host qubits. The voltages listed in this figure were also updated (slightly) to reflect the actual device state when the "final" $d = 5$ data was taken.

20) It is not clear what factors limited the number of EO qubits utilized on the chip. Is this primarily a matter of measurement time, qubit yield, controller limitations, or other constraints? Please clarify the dominant bottlenecks and whether higher qubit counts have been used but are not shown.

This work is principally focused on the system components and their integration and validation. We demonstrate the capability of the integrated cryo-controller and interconnect to control our qubit device, up to and including mid-circuit measurement and initialization sufficient for many repeated rounds of error correction. We expect that an integrated system of the type we present here is capable of entangling all 54 spins in 54 dots with sufficient control and measurement fidelity to be used in a computation, though that is not a claim of this paper. We do acknowledge here that higher qubit counts with improved performance have been utilized in the lab (largely a result of more measurement time), but those data will be synthesized into a separate publication

in order to keep the present work focused on system integration.

Response to Referee #2

> *1) The paper highlights an advancement of spin qubit performance in several places. Excellent. However, the paper largely ignores the performance of spin qubits compared to other qubit modalities. Are spin qubits now competitive with leading superconducting, ion trap, and neutral atom quantum systems. I believe that the authors would say "yes." Including a cursory comparison would amplify their messages.*

Referee #2 correctly guesses our feelings on this matter, but we do not think it is appropriate to use this scientific paper as a venue to explicitly argue the point beyond the concluding sentence of "the scale and cost of a system anticipated by the prototype reported here position semiconductor spin qubits as a leading technology to realize commercially-relevant quantum computing". We hope that over time, and in no small part due to this publication, the broader quantum information community will come to agree with our view on the competitive status of spin qubits.

> *2) The authors do a thorough job of discussing their error pareto throughout. They mention in the Qubit Chip Methods section that they are using superconducting on-chip wires. Context here is requested. Does the presence (or absence) contribute to the qubit performance at this scale or is it necessary only for larger systems. This is largely the only qubit feature that is not discussed in more detail.*

Superconducting on-chip wires (in the large-pitch back end of line, large-pitch BEOL) are important for signal fidelity. Simulations suggest that at the scale, speed and fidelity of the present demonstration, non-superconducting large-pitch BEOL would be "on the fuzzy border" of viability. Had the large-pitch BEOL used normal metal, the resulting additional pulse distortions and crosstalk would be less than those arising elsewhere in the signal chain, but would likely have pushed us to slower operation speed. However, as we improve fidelity related to contextual miscalibration in the future, the improved signal fidelity from superconducting large-pitch BEOL will become increasingly necessary. Note that the superconducting large-pitch BEOL also complicates having an external magnetic field, as we mention in the methods; as such we operate at very close to zero magnetic field.

We believe further detail in the manuscript about this "present but not critical" aspect of the prototype would distract from the many, more performance-relevant aspects, but appreciate the opportunity to clarify this facet of the technology here.

This referee notes the term LRU is used but not defined in line 157. It is only defined in the text of Figure 4.

We thank Referee #2 for catching this and now spell this out in main text as well as in the caption.

Response to Referee #3

One point to note is that the caption of Fig. 3 suggests that a different device was used to obtain the results shown in that figure. It would be interesting to know how many devices were measured. This is relevant given the discussion about commercial relevance in the conclusion.

The data presented in the main text of this manuscript come from three different qubit chips. One for the ECDFs of T_2^* and N_{osc} measurements, one for the repetition code data, and one for the ECDFs of randomized benchmarking error and the [[4,2,2]] data. Additionally, Supplemental Information Fig. S23 shows initialization window widths from nine separate devices and Fig. S28 provides room temperature wafer probe data on a full device lot. We apologize for this complication, but believe the text provided adequately explains it. As to the total number of qubit chips of this type measured, we are unfortunately not able to provide the exact number, but it is larger than those specifically delineated here.

In the abstract, the authors state that the 54-quantum-dot architecture could support up to 18 exchange-only qubits. While the number of quantum dots in this chip is in principle sufficient to encode 18 exchange-only qubits, the demonstrated algorithms involve no more than seven qubits in the largest implementation. It would be helpful to clarify whether these demonstrations always used the same seven qubits, and how representative these qubits are of the full array.

Three different configurations of qubits were used for the [5,1,5], optimized [3,1,3], and [[4,2,2]] data. These are identified precisely in the Supplementary Information in figures S44, S40, and S49 respectively. Notably, all of these qubits use the "alternative readout configuration" described in the supplementary information in section S8.A, which trades improved readout performance for reduction in the maximum number of qubits available. While early iterations of the integrated system were affected by yield issues in all components, later versions have had essentially perfect wire functional yield (as shown in Fig. S28), and the maximum number of qubits used was principally limited by available time. We expect to clearly demonstrate use of the entire chip in future work. In terms of performant yield, we also note that the ECDFs of charge and magnetic noise, shown in figure 3, represent 14 of 18 possible qubits (see also Fig. S26), which was also limited by time constraints rather than device properties.

As shown in Fig. 1, the device includes only a single row of dot charge sensors located at the bottom of the array. In the Supplementary Information, the authors describe an "alternative readout configuration" in which dots within the qubit array are repurposed to enable readout of a smaller subset of qubits with improved charge sensitivity. It is therefore unclear how high-fidelity readout would be achieved when the array is fully populated.

In the prototype QPU used in this work, two factors led us to use this "flood forward" technique. First, the device design did not jointly optimize DCS sensitivity and charge stability; we have since learned improvements to this optimization, and so this is revised in newer gate designs. Second, the sample daughterboard was not originally designed with sufficient care with respect to readout signal integrity. These issues are what we are referring to in the text when saying "modifications to the gate geometry and improvements to the measurement chain will render this technique obsolete going forward". In experimental work subsequent to what we report on here, we have found that a revised daughterboard by itself is sufficient to obviate the "flood forward" technique.

Fig. 4 describes a large number of operations required for a single instance of the 200-round experiment, including over 1,800 initialisations, 800 measurements, and 330,000 exchange operations. What is the laboratory time required for a single instance? This information would allow useful comparison with other platforms and with other spin-qubit processors.

We have added information about the pulse-to-pulse cadence (varying between 12 and 28 ns) to the caption of figure 3. In the text we note that single qubit operations take 4 timesteps, SWAPs take 15, RILs 30, and CNOTs 45. We have added additional information to the Supplementary Information about the duration of initialization and measurement operations. We thank the referee for calling our attention to this, as the previous text left the impression that SPAM durations were equivalent to that of Blumoff et al., and this is not the case for the data taken here. The duration of a full round of syndrome extraction is roughly 90 μs, dominated by the SPAM operations.

In the closing statements, the authors claim that conventional concerns such as charge noise, valley splitting, and leakage out of the computational space are no longer limiting. This statement may be questioned. The shot rejection in the [[4,2,2]] post-selected analysis seems high.

The experimentally measured shot rejection for [[4,2,2]], inclusive of the effects of "low charge noise, high valley splitting," and all "leakage out of the computational space" as handled by LRU circuits, is consistent with our error modelling. This is why we find it important in Fig. 4e to compare the experimental throughput (black points) which those simulated with both spin and

event simulators; see also supplementary figures S42, S51, and S52 and accompanying text. The throughput is of course limited by error, and error is, as we have argued more significantly in the supplement than the main text, limited by contextual pulse miscalibration errors occurring in the integrated system. The reviewer's question can be rephrased as the following true statement: if our error were limited by the "conventional concerns", then the shot rejection in [[4,2,2]] would be "too high." A key observation of our work is that we are limited now by extrinsic concerns, less conventionally modeled in spin qubits, arising from the integration of our cryo-controlled system, and which we are now engaged in reducing via engineering techniques employed in integration rather than in epitaxial or other fabrication techniques.

> *Also, work on valley-splitting engineering suggests that improvements can come at the cost of reduced mobility. Providing the range of mobilities achieved would help clarify the extent to which the hardware advances presented in this paper solve a long-standing problem in silicon qubit devices.*

We have revised the discussion of valley splitting in the main text and SI to clarify several points, as further detailed in our response to reviewer 1. Unfortunately, characterization details of our epitaxial stack are proprietary, but we will note that the mobility in our structures is not an operational concern.

> *Finally, how would this wiring strategy scale, given the size constraints associated with indium bumps/bond pads?*

As we discussed in the response to Referee #1's question #3.1, we agree that the packaging and interconnect density used in this work is not sufficient for utility scale without modification. We anticipate that a combination of straightforward engineering solutions to increase the density of the qubit-interconnect interface as well as replacing the ribbon cable with a larger structure still utilizing superconducting wires could achieve necessary scale. These discussions are out of scope for the present manuscript, however.

> *In Fig. 3a, the legend and vertical reference lines do not appear to match the mean error values reported in the figure caption?*

We thank Referee #3 for catching this discrepancy. The vertical reference lines in the original figure are the *median* values but the error values in the figure caption are the *mean*. We have changed the figure to use the *mean*, as appears to be standard in the literature (e.g. the Google "Willow" paper). We have also updated the caption to use the same numbers (e.g. two significant figures) to avoid confusion. Note that the "quartile" markings in sub-figures b and c are still

based on median distribution values.

> *In Figs. 3b-c, the reported number of observed oscillations and the median T_2^* values appear to imply characteristic frequencies that can differ from the exchange-frequency values quoted in the Supplementary Materials.*

We believe the miscommunication leading to this question is the assumption that T_2^* represents a decay timescale for which N_{osc} is the corresponding number of oscillations, and as such the ratio of the two would be an "exchange frequency." This is not a correct assumption, and so the resulting exchange frequencies from that ratio are unsurprisingly very different from actual exchange-frequencies under use. We have received similar questions in the past conflating these two metrics. In fact they correspond to different experiments and different underlying physical sources. N_{osc} describes the number of coherent oscillations the spin-spin exchange interaction can support until it dephases, while T_2^* defines a characteristic dephasing time observed when spins are isolated, i.e. undergoing no exchange at all. N_{osc} is ultimately a metric for charge noise, and T_2^* is ultimately a metric for magnetic noise. N_{osc} is in fact the *better* metric for charge noise relative to a decay timescale, because the absolute timescale of decay of exchange oscillations scales inversely with frequency for exponentially activated exchange, and hence their product (or "Q") is (roughly) a constant that is more important than either the exchange frequency or its decay timescale alone. For magnetic decay, the timescale is the better metric, as (ideally) there are no singlet-triplet oscillations at all when spins are isolated. The separation of sources for these two experiments is not always clean (T_2^* can be polluted by exchange noise; see Maune et al., *Nature* 2012 for an early example from us, and N_{osc} can be polluted by magnetic noise; see Ladd *PRB* 2012 for the theory and Medford et al., *Nature Nanotech.* 2013 for a dramatic example in GaAs) but in the present generation of exchange-only qubit device, the two distinct metrics almost fully distinguish the two distinct noise sources.

We thank the referee for highlighting this instance of insufficient clarity. Rehashing this decades-old story at the level above in the text does not warrant the space it would take in the present release, but to help some readers, we have added one extra sentence to the main text near line 122 to address this common point of confusion; more clarity would surely require a deep dive into the many cited references using these metrics. In a new sentence on line 122, after "... from a qubit chip of the same types as those used for the multiqubit experiments," we add "The metric N_{osc} characterizes error due to intrinsic charge noise for spins undergoing exchange, while

the metric T_2^* characterizes error due to magnetic noise for idling spins." The addition of this sentence allows a more precise revision of the following two sentences, which now say "The intrinsic charge noise reflected by these N_{osc} measurements improve on the prior state of the art by approximately a factor of ten. Errors resulting from the mean values of N_{osc} and T_2^* would collectively contribute only 0.02% to absolute CNOT error."

> *In Fig. 4c [now changed to 4b], light-shaded triangular markers are shown alongside solid data points. Their meaning could be explained in the figure caption or the main text, including why no analogous square markers are shown. Similarly, the meaning of the error bars associated with fidelity and throughput in Fig. 4e could be specified in the caption.*

These are now elaborated in the caption. It does take a close look to note that there are multiple light-red triangles on Fig. 4b (formerly 4c), which correspond to different [3,1,3] subsets of data from the [5,1,5] experiment, that average together to form the dark red triangle.

> *For the temperature estimates shown in Fig. S14, it would be helpful to provide fits and gate lever arms used to extract the electron temperatures.*

We have added some example fits into Fig. S14, with associated explanation in the caption. The fits behave well and give reliable values of lever arms α and electron temperatures T_{e}. Across a single device, and verified on multiple devices, lever arms extracted from this procedure vary at most by 7% from 0.1 eV/V.
